# Supplementary material for: Access to primary care for children and young people (CYP) in the UK: a scoping review of CYP’s, caregivers’ and healthcare professionals’ views and experiences of facilitators and barriers
Source: BMJ Open. 2024 May 30;14(5):e081620. doi: 10.1136/bmjopen-2023-081620 (PMC11141190; doi:10.1136/bmjopen-2023-081620)
Supplement: Supplementary data [file bmjopen-2023-081620supp002.pdf]

Additional file 2 – Search terms

Cinahl Plus search

(MH “Health Services Accessibility”) or (MH “Quality of Health Care”) or (MH “Help Seeking Behavior”) or (TI(“access to health service*” or “access to care” or “access to health care” or “health equity” or inequal* or equality or disparit* or unequal or gap* or gradient* or disadvantage*)) or (AB(“access to health service*” or “access to care” or “access to health care” or “health equity” or inequal* or equality or disparit* or unequal or gap* or gradient* or disadvantage*)) AND (MH “Primary Health Care”) or (MH “Primary Nursing”) or (MH “Physicians, Family”) or (MH “Family Practice”) or (TI(“primary health care” or “primary care” or “general practice*”) or “GP surger*” or “dentist*” or “general practitioner*” or “community pharmac*”) or (AB(“primary health care” or “primary care” or “general practice*” or “community pharmac*”) AND (MH Child) or (MH Adolescence) or (MH Infant) or (MH “Parent-Child Relations”) or (TI(child* or adolescen* or infant* or “young people” or youth or juvenile* or teenager* or student* or pupil* or “young adult*” or preschool*)) or (AB(child* or adolescen* or infant* or “young people” or youth or juvenile* or teenager* or student* or pupil* or “young adult*” or preschool*)) AND (MH "United Kingdom") or (MH England) or (MH Wales) or (MH Scotland) or (MH “Northern Ireland”) or (MH “Great Britain”) or (TX(UK or “United Kingdom" or England or Wales or Scotland or “Northern Ireland” or “N. Ireland” or “Great Britain”) AND (MH “Patient Satisfaction”) or (MH “Patient Preference”) or (MH “Health Knowledge”) or (TI(perceived or experience* or “attitude* to health” or facilitator* or enabler* or barrier* or promot* or inhibit* or view* or perspective*) or (AB(perceived or experience* or “attitude* to health” or facilitator* or enabler* or barrier* or promot* or inhibit* or view* or perspective*)

Filter: 2012-2022

Psycinfo search

((Health Care Access).sh. or (Health Care Utilization).sh. or (Health Disparities).sh. or (Help Seeking Behavior).sh. or (Health Care Seeking Behavior).sh. or (Quality of Care).sh. or (access to health service*).ti,ab. or (access to care).ti,ab. or (access to health care).ti,ab. or (health equity).ti,ab. or (inequal*).ti,ab. or (equality).ti,ab. or (disparit*).ti,ab. or (unequal).ti,ab. or (gap*).ti,ab. or (gradient*).ti,ab. or (disadvantage*).ti,ab.) AND ((Primary Health Care).sh. or (General Practitioners).sh. or (Family Physicians).sh. or (Pharmacy).sh. or (Dentists).sh. or (primary health care).ti,ab. or (primary care).ti,ab. or (general practice*).ti,ab. or (GP surger*).ti,ab. or (dentist*).ti,ab. or (general practitioner*).ti,ab. or (community pharmac*).ti,ab.) AND ((Child Behavior).sh. or (Early Adolescence).sh. or (Adolescent Psychology).sh. or (Parent-Child Relations).sh. or (child*).ti,ab. or (adolescen*).ti,ab. or (infant*).ti,ab. or (young people).ti,ab. or (youth).ti,ab. or (juvenile*).ti,ab. or (teenager*).ti,ab. or (student*).ti,ab. or (pupil*).ti,ab. or (young adult*) or (preschool).ti,ab.) AND ((United Kingdom).af. or (England).af. or (Wales).af. or (Scotland).af. or (Northern Ireland).af. or (Great Britain).af.) AND ((Client Satisfaction).sh. or (Client Attitudes).sh. or (Health Knowledge).sh. or (Treatment Barriers).sh. or (perceived).ti,ab. or (experience*).ti,ab. or (attitude* to health).ti,ab. or (facilitator*).ti,ab. or (enabler*).ti,ab. or (barrier*).ti,ab. or (promot*).ti,ab. or (inhibit*).ti,ab. or (view*).ti,ab. or (perspective*).ti,ab.)

Filter: 2012-2022

Web of Science Core Collection Social Sciences Citation Index (SSCI)

(TS=(“health care access” OR “help seeking behavior” OR “help seeking behaviour” OR “quality of care” OR “access to health service*” OR “access to care” OR “access to health care” OR “health equity” or inequal* or equality OR disparit* OR unequal OR gap* OR gradient* OR disadvantage*)) AND (TS=(“primary health care” OR “general practitioner*” OR “family physician*” OR “primary care” OR “general practice*” OR “GP surger*” OR dentist* OR “dental care” OR “community pharmac*”)) AND (TS=(“child behavior” OR “early adolescence” OR “adolescent psychology” OR “parent-child relation*” OR child* OR adolescen* OR infant* OR “young people” OR youth OR juvenile* OR teenage* OR student* OR pupil* OR “young adult*” OR preschool)) AND (ALL=(“United Kingdom” OR England OR Wales OR Scotland OR “Northern Ireland” OR “Great Britain”)) AND (TS=(“patient satisfaction” OR “patient preference*” OR “health knowledge” OR perceived OR experience* OR “attitude* to health” OR facilitator* OR enabler* OR barrier* OR promot* OR inhib* OR view* OR perspective*))

Filter: 2012-2022

SCOPUS

ALL((Health Services Accessibility OR "access to health services" OR "access to care" OR Health Equity OR "health equity" OR inequality OR inequalities OR equality OR disparity OR disparities OR unequal OR gap OR gaps OR gradients OR disadvantage OR health service utilisation OR health service utilisation OR "health resource utilisation" OR "health resource utilisation" OR health care seeking behaviour OR health care seeking behavior OR Health Care Quality OR Health Care Evaluation) AND (Primary Health Care OR "primary health care" OR "primary care" OR Primary Care Nursing OR Physicians Primary Care OR General Practice OR "general practice*" OR "GP surger*" OR General Practice Dental OR "dentist*" OR General Practitioners OR "general practitioner*" OR Community Pharmacy Services OR "community pharmac*" or "health visitor" OR pediatric care OR paediatric care) AND (Child or child* or Adolescent or adolescen* or Infant or infant* or "young people" or youth or juvenile* or teenager* or "young adult*" OR child* pre-school OR child* health) AND ("United Kingdom" OR England OR Wales OR Scotland OR "Northern Ireland" OR "N. Ireland") AND (Perception* OR perceived OR experience* OR Patient Satisfaction OR Patient Preference OR Attitude to Health OR Facilitator* OR enabler* OR barrier* OR Patient Acceptance of Health Care)) AND PUBYEAR > 2011 AND PUBYEAR < 2023 AND (LIMIT-TO (LANGUAGE,"English"))

Pubmed

Search: (("health services accessibility"[MeSH Terms] OR ("health"[All Fields] AND "services"[All Fields] AND "accessibility"[All Fields]) OR "health services accessibility"[All Fields] OR "access to health services"[All Fields] OR "access to care"[All Fields] OR ("health equity"[MeSH Terms] OR ("health"[All Fields] AND "equity"[All Fields]) OR "health equity"[All Fields]) OR "health equity"[All Fields] OR ("inequalities"[All Fields] OR "inequality"[All Fields] OR "inequities"[All Fields] OR "inequity"[All Fields]) OR ("inequalities"[All Fields] OR "inequality"[All Fields] OR "inequities"[All Fields] OR "inequity"[All Fields]) OR ("equal"[All Fields] OR "equaled"[All Fields] OR "equaling"[All Fields] OR "equalisation"[All Fields] OR "equalise"[All Fields] OR "equalised"[All Fields] OR "equalises"[All Fields] OR "equalising"[All Fields] OR "equalities"[All Fields] OR "equality"[All Fields] OR "equalization"[All Fields] OR "equalizations"[All Fields] OR "equalize"[All Fields] OR "equalized"[All Fields] OR "equalizer"[All Fields] OR "equalizers"[All Fields] OR "equalizes"[All Fields] OR "equalizing"[All Fields] OR "equalled"[All Fields] OR "equalling"[All Fields] OR "equally"[All Fields] OR "equals"[All Fields]) OR ("disparate"[All Fields] OR "disparately"[All Fields] OR "disparities"[All Fields] OR "disparity"[All Fields]) OR

("disparate"[All Fields] OR "disparately"[All Fields] OR "disparities"[All Fields] OR "disparity"[All Fields]) OR ("unequal"[All Fields] OR "unequally"[All Fields] OR "unequals"[All Fields]) OR "gap"[All Fields] OR "gaps"[All Fields] OR ("gradient"[All Fields] OR "gradient s"[All Fields] OR "gradients"[All Fields]) OR ("disadvantage"[All Fields] OR "disadvantageous"[All Fields] OR "disadvantageously"[All Fields] OR "disadvantages"[All Fields] OR "disadvantaging"[All Fields] OR "vulnerable populations"[MeSH Terms] OR ("vulnerable"[All Fields] AND "populations"[All Fields]) OR "vulnerable populations"[All Fields] OR "disadvantaged"[All Fields]) OR (("health services"[MeSH Terms] OR ("health"[All Fields] AND "services"[All Fields]) OR "health services"[All Fields] OR ("health"[All Fields] AND "service"[All Fields]) OR "health service"[All Fields]) AND ("statistics and numerical data"[MeSH Subheading] OR ("statistics"[All Fields] AND "numerical"[All Fields] AND "data"[All Fields]) OR "statistics and numerical data"[All Fields] OR "utilization"[All Fields] OR "utilisation"[All Fields] OR "utilisations"[All Fields] OR "utilise"[All Fields] OR "utilised"[All Fields] OR "utilises"[All Fields] OR "utilising"[All Fields] OR "utilities"[All Fields] OR "utility"[All Fields] OR "utilizations"[All Fields] OR "utilize"[All Fields] OR "utilized"[All Fields] OR "utilizer"[All Fields] OR "utilizers"[All Fields] OR "utilizes"[All Fields] OR "utilizing"[All Fields])) OR (("health services"[MeSH Terms] OR ("health"[All Fields] AND "services"[All Fields]) OR "health services"[All Fields] OR ("health"[All Fields] AND "service"[All Fields]) OR "health service"[All Fields]) AND ("statistics and numerical data"[MeSH Subheading] OR ("statistics"[All Fields] AND "numerical"[All Fields] AND "data"[All Fields]) OR "statistics and numerical data"[All Fields] OR "utilization"[All Fields] OR "utilisation"[All Fields] OR "utilisations"[All Fields] OR "utilise"[All Fields] OR "utilised"[All Fields] OR "utilises"[All Fields] OR "utilising"[All Fields] OR "utilities"[All Fields] OR "utility"[All Fields] OR "utilizations"[All Fields] OR "utilize"[All Fields] OR "utilized"[All Fields] OR "utilizer"[All Fields] OR "utilizers"[All Fields] OR "utilizes"[All Fields] OR "utilizing"[All Fields])) OR "health resource utilisation"[All Fields] OR "health resource utilisation"[All Fields] OR ("health care seeking behaviour"[All Fields] OR "patient acceptance of health care"[MeSH Terms] OR ("patient"[All Fields] AND "acceptance"[All Fields] AND "health"[All Fields] AND "care"[All Fields]) OR "patient acceptance of health care"[All Fields] OR ("health"[All Fields] AND "care"[All Fields] AND "seeking"[All Fields] AND "behavior"[All Fields]) OR "health care seeking behavior"[All Fields]) OR ("health care seeking behaviour"[All Fields] OR "patient acceptance of health care"[MeSH Terms] OR ("patient"[All Fields] AND "acceptance"[All Fields] AND "health"[All Fields] AND "care"[All Fields]) OR "patient acceptance of health care"[All Fields] OR ("health"[All Fields] AND "care"[All Fields] AND "seeking"[All Fields] AND "behavior"[All Fields]) OR "health care seeking behavior"[All Fields]) OR ("quality of health care"[MeSH Terms] OR ("quality"[All Fields] AND "health"[All Fields] AND "care"[All Fields]) OR "quality of health care"[All Fields] OR ("health"[All Fields] AND "care"[All Fields] AND "quality"[All Fields]) OR "health care quality"[All Fields]) OR (("delivery of health care"[MeSH Terms] OR ("delivery"[All Fields] AND "health"[All Fields] AND "care"[All Fields]) OR "delivery of health care"[All Fields] OR ("health"[All Fields] AND "care"[All Fields]) OR "health care"[All Fields]) AND ("evaluability"[All Fields] OR "evaluate"[All Fields] OR "evaluated"[All Fields] OR "evaluates"[All Fields] OR "evaluating"[All Fields] OR "evaluation"[All Fields] OR "evaluation s"[All Fields] OR "evaluations"[All Fields] OR "evaluative"[All Fields] OR "evaluatively"[All Fields] OR "evaluatives"[All Fields] OR "evaluator"[All Fields] OR "evaluator s"[All Fields] OR "evaluators"[All Fields]))) AND ("primary health care"[MeSH Terms] OR ("primary"[All Fields] AND "health"[All Fields] AND "care"[All Fields]) OR "primary health care"[All Fields] OR "primary health care"[All Fields] OR "primary care"[All Fields] OR ("primary nursing"[MeSH Terms] OR ("primary"[All Fields] AND "nursing"[All Fields]) OR "primary nursing"[All Fields] OR ("primary"[All Fields] AND "care"[All Fields] AND "nursing"[All Fields]) OR "primary care nursing"[All Fields] OR "primary care nursing"[MeSH Terms] OR ("primary"[All Fields] AND "care"[All Fields] AND "nursing"[All Fields])) OR ("physicians, primary care"[MeSH Terms] OR ("physicians"[All Fields] AND

"primary"[All Fields] AND "care"[All Fields]) OR "primary care physicians"[All Fields] OR ("physicians"[All Fields] AND "primary"[All Fields] AND "care"[All Fields]) OR "physicians primary care"[All Fields]) OR ("general practice"[MeSH Terms] OR ("general"[All Fields] AND "practice"[All Fields]) OR "general practice"[All Fields]) OR "general practice"[All Fields] OR "gp surger*"[All Fields] OR ("general practice, dental"[MeSH Terms] OR ("general"[All Fields] AND "practice"[All Fields] AND "dental"[All Fields]) OR "dental general practice"[All Fields] OR ("general"[All Fields] AND "practice"[All Fields] AND "dental"[All Fields]) OR "general practice dental"[All Fields]) OR "dentist*"[All Fields] OR ("general practitioners"[MeSH Terms] OR ("general"[All Fields] AND "practitioners"[All Fields]) OR "general practitioners"[All Fields]) OR "general practitioner*"[All Fields] OR ("community pharmacy services"[MeSH Terms] OR ("community"[All Fields] AND "pharmacy"[All Fields] AND "services"[All Fields]) OR "community pharmacy services"[All Fields]) OR "community pharmac*"[All Fields] OR "health visitor"[All Fields] OR ("pediatr care wilmington"[Journal] OR ("pediatric"[All Fields] AND "care"[All Fields]) OR "pediatric care"[All Fields]) OR (("paediatrics"[All Fields] OR "pediatrics"[MeSH Terms] OR "pediatrics"[All Fields] OR "paediatric"[All Fields] OR "pediatric"[All Fields]) AND "care"[All Fields])) AND ("child"[MeSH Terms] OR "child"[All Fields] OR "children"[All Fields] OR "child s"[All Fields] OR "children s"[All Fields] OR "childrens"[All Fields] OR "childs"[All Fields] OR "child*"[All Fields] OR ("adolescences"[All Fields] OR "adolescency"[All Fields] OR "adolescent"[MeSH Terms] OR "adolescent"[All Fields] OR "adolescence"[All Fields] OR "adolescents"[All Fields] OR "adolescent s"[All Fields]) OR "adolescen*"[All Fields] OR ("infant"[MeSH Terms] OR "infant"[All Fields] OR "infants"[All Fields] OR "infant s"[All Fields]) OR "infant*"[All Fields] OR "young people"[All Fields] OR ("adolescent"[MeSH Terms] OR "adolescent"[All Fields] OR "youth"[All Fields] OR "youths"[All Fields] OR "youth s"[All Fields]) OR "juvenile*"[All Fields] OR "teenager*"[All Fields] OR "young adult*"[All Fields] OR ("child*"[All Fields] AND ("child, preschool"[MeSH Terms] OR ("child"[All Fields] AND "preschool"[All Fields]) OR "preschool child"[All Fields] OR ("pre"[All Fields] AND "school"[All Fields]) OR "pre school"[All Fields])) OR ("child*"[All Fields] AND ("health"[MeSH Terms] OR "health"[All Fields] OR "health s"[All Fields] OR "healthful"[All Fields] OR "healthfulness"[All Fields] OR "healths"[All Fields]))) AND ("United Kingdom"[All Fields] OR ("england"[MeSH Terms] OR "england"[All Fields] OR "england s"[All Fields] OR "englands"[All Fields]) OR ("wales"[MeSH Terms] OR "wales"[All Fields] OR "wales s"[All Fields]) OR ("scotland"[MeSH Terms] OR "scotland"[All Fields] OR "scotland s"[All Fields]) OR "Northern Ireland"[All Fields] OR "n ireland"[All Fields]) AND ("perception*"[All Fields] OR ("perceivable"[All Fields] OR "perceive"[All Fields] OR "perceiver"[All Fields] OR "perceiver s"[All Fields] OR "perceivers"[All Fields] OR "perceives"[All Fields] OR "perception"[MeSH Terms] OR "perception"[All Fields] OR "perceived"[All Fields] OR "perceiving"[All Fields]) OR "experience*"[All Fields] OR ("patient satisfaction"[MeSH Terms] OR ("patient"[All Fields] AND "satisfaction"[All Fields]) OR "patient satisfaction"[All Fields]) OR ("patient preference"[MeSH Terms] OR ("patient"[All Fields] AND "preference"[All Fields]) OR "patient preference"[All Fields]) OR ("attitude to health"[MeSH Terms] OR ("attitude"[All Fields] AND "health"[All Fields]) OR "attitude to health"[All Fields]) OR "facilitator*"[All Fields] OR "enabler*"[All Fields] OR "barrier*"[All Fields] OR ("patient acceptance of health care"[MeSH Terms] OR ("patient"[All Fields] AND "acceptance"[All Fields] AND "health"[All Fields] AND "care"[All Fields]) OR "patient acceptance of health care"[All Fields]))) AND ((2012/1/1:2022/2/21[pdat]) AND (english[Filter]))*
