# Supplementary material for: Access to primary care for children and young people (CYP) in the UK: a scoping review of CYP’s, caregivers’ and healthcare professionals’ views and experiences of facilitators and barriers
Source: BMJ Open. 2024 May 30;14(5):e081620. doi: 10.1136/bmjopen-2023-081620 (PMC11141190; doi:10.1136/bmjopen-2023-081620)
Supplement: Supplementary data [file bmjopen-2023-081620supp003.pdf]

Additional file 3: Quality assessment

To achieve 'high' quality, at least five MMAT criteria had to be met, with breadth and depth of analysis, for 'medium' at least four criteria had to be met, and all other studies were rated 'low'. For a judgement of 'high' relevance, studies had to describe, with breadth and depth, factors influencing primary care access and privilege participants' perspectives.

NB there were no randomized controlled trials in the studies so the MMAT questions for section 2 have been removed here.

**SCREENING QUESTIONS**	S1. Are there clear research questions?<br>S2. Do the collected data allow to address the research questions?
**1. QUALITATIVE STUDIES**	1.1. Is the qualitative approach appropriate to answer the research question?<br>1.2. Are the qualitative data collection methods adequate to address the research question?<br>1.3. Are the findings adequately derived from the data?<br>1.4. Is the interpretation of results sufficiently substantiated by data?<br>1.5. Is there coherence between qualitative data sources, collection, analysis and interpretation?
**3. NON-RANDOMIZED STUDIES**	3.1. Are the participants representative of the target population?<br>3.2. Are measurements appropriate regarding both the outcome and intervention (or exposure)?<br>3.3. Are there complete outcome data?<br>3.4. Are the confounders accounted for in the design and analysis?<br>3.5. During the study period, is the intervention administered (or exposure occurred) as intended?
**4. QUANTITATIVE DESCRIPTIVE STUDIES**	4.1. Is the sampling strategy relevant to address the research question?<br>4.2. Is the sample representative of the target population?<br>4.3. Are the measurements appropriate?<br>4.4. Is the risk of nonresponse bias low?<br>4.5. Is the statistical analysis appropriate to answer the research question?
**5. MIXED METHODS STUDIES**	5.1. Is there an adequate rationale for using a mixed methods design to address the research question?<br>5.2. Are the different components of the study effectively integrated to answer the research question?<br>5.3. Are the outputs of the integration of qualitative and quantitative components adequately interpreted?<br>5.4. Are divergences and inconsistencies between quantitative and qualitative results adequately addressed?<br>5.5. Do the different components of the study adhere to the quality criteria of each tradition of the methods involved?

		SCREENING QUESTIONS		1. QUALITATIVE STUDIES					Total MMAT	W1 - quality	W2 - relevance
First author	Year	S1	S2	1.1	1.2	1.3	1.4	1.5			
Ahmaro et al	2021	Yes	Yes	Yes	Yes	Yes	Yes	Yes	5	High	High
Alexakis et al	2015	Yes	Yes	Yes	Yes	Yes	Yes	Yes	5	High	High
Appleton et al	2022	Yes	Yes	Yes	Yes	Yes	Yes	Yes	5	High	High
Bosley et al	2021	Yes	Yes	Yes	Yes	Yes	Yes	Yes	5	Medium	Medium
Brigham et al	2012	Can't tell	Can't tell	Yes	Yes	Yes	No	Can't tell	3	Low	Low
Coleman-Fountain et al	2020	Yes	Yes	Yes	Yes	Yes	Yes	Yes	5	High	Low
Condon et al	2020	Yes	Yes	Yes	Yes	Yes	Yes	Yes	5	High	Low
Corry and Leavey	2017	Yes	Yes	Yes	Yes	Yes	Yes	Yes	5	High	High
Crouch et al	2019	Yes	Yes	Yes	Yes	Yes	Yes	Yes	5	High	Medium
Dando et al	2019	Yes	Yes	Yes	Yes	Yes	Yes	Can't tell	4	Low	Low
Davey et al	2013	Yes	Yes	Yes	Yes	Yes	Can't tell	Yes	4	Medium	High
Dickson	2015	Yes	Yes	Yes	Yes	Yes	No	No	3	Low	Medium
Diwakar et al	2019	Yes	Yes	Yes	No	Yes	Yes	Yes	4	Medium	Medium
Eskytė et al	2021	Yes	Yes	Yes	Yes	Yes	Yes	Yes	5	High	Low
Fox et al	2017	Yes	Can't tell	Yes	Yes	Yes	Yes	Yes	5	High	High
French et al	2020	Yes	Yes	Yes	Yes	Yes	Yes	Yes	5	Medium	High
Henderson and Rubin	2014	Can't tell	Yes	No	No	Can't tell	Yes	Can't tell	3	Low	Low
Ingram et al	2013	Yes	Yes	Yes	Yes	Yes	Yes	No	4	Medium	High
Jobanputra and Singh	2020	Yes	Yes	Yes	No	Yes	No	Yes	3	Low	Medium
Jones et al	2017	Yes	Yes	Yes	Yes	Yes	Yes	Yes	5	High	High
Lewney et al	2019	Yes	Yes	Yes	Yes	Yes	Yes	Yes	5	High	Medium
McDonagh et al	2019	Yes	Yes	Yes	No	Yes	Yes	Yes	4	Medium	High
Mughal et al	2021	Yes	Yes	Yes	Yes	Yes	Yes	Can't tell	4	Medium	High
Muirhead et al	2017	Yes	Yes	Yes	Yes	Can't tell	Yes	Yes	4	Medium	Low
Neill et al	2016	Yes	Yes	Yes	Yes	Yes	Yes	Yes	5	High	High
Neill et al	2015	Yes	Yes	Yes	Yes	Yes	Yes	Yes	5	High	High
O'Brien et al	2017	Yes	Yes	Yes	Yes	Yes	Yes	Yes	5	High	High
Ochieng	2020	Yes	Yes	Yes	Yes	Yes	Yes	Yes	5	High	Low
Rapley et al	2021	Yes	Yes	Yes	Yes	Yes	Yes	Yes	5	High	High
Redsell et al	2013	Yes	Can't tell	Yes	Can't tell	No	Yes	No	2	Low	Low

Roberts et al	2014	Yes	Can't tell	Yes	No	Yes	Yes	No	3	Low	High
Roberts and Condon	2014	Yes	Yes	Yes	No	Yes	Yes	Yes	4	Low	Low
Satherley et al	2021	Yes	Yes	Yes	Yes	Yes	Yes	Yes	5	High	High
Turnbull et al	2021	Yes	Yes	Yes	Yes	Can't tell	Yes	Yes	4	Medium	High
Turner et al	2012	Yes	Yes	Can't tell	Yes	Yes	No	Yes	3	Low	High
Williams et al	2014	Yes	Yes	Yes	Can't tell	Yes	Yes	Yes	4	Low	Low
Williams et al	2012	No	Yes	Yes	Yes	Yes	Yes	Yes	5	Medium	Medium

		**SCREENING QUESTIONS**		**3. NON-RANDOMIZED STUDIES**					**Total MMAT**	**W1 - quality**	**W2 - relevance**
First author	Year	S1	S2	3.1	3.2	3.3	3.4	3.5			
O'Brien et al	2019	Yes	Yes	Yes	Yes	Yes	Yes	Yes	5	High	Low
Usher-Smith et al	2015	Yes	Can't tell	Yes	Yes	Yes	Can't tell	Yes	4	Medium	Medium
Yassaee et al	2017	Yes	Yes	Yes	Yes	Yes	Can't tell	Yes	4	High	Low

		**SCREENING QUESTIONS**		**4. QUANTITATIVE DESCRIPTIVE STUDIES**					**Total MMAT**	**W1 - quality**	**W2 - relevance**
First author	Year	S1	S2	4.1	4.2	4.3	4.4	4.5			
Coyle et al	2013	Yes	Yes	Can't tell	No	Yes	Yes	No	2	Low	Low

		**SCREENING QUESTIONS**		**5. MIXED METHODS STUDIES**					**Total MMAT**	**W1 - quality**	**W2 - relevance**
First author	Year	S1	S2	5.1	5.2	5.3	5.4	5.5			
Crocker et al	2013	Yes	Yes	Yes	Yes	Yes	Yes	No	4	Low	Low
Fox et al	2015	Yes	Yes	Yes	Yes	Yes	No	Yes	4	Medium	Medium
Rashed et al	2022	Yes	Yes	Can't tell	Can't tell	Yes	Yes	Can't tell	2	Low	Medium
Rickett et al	2021	Yes	Yes	Yes	Yes	Yes	Yes	Yes	5	Medium	High
Salaheddin and Mason	2016	Yes	Yes	Can't tell	Yes	Yes	Can't tell	Yes	3	Low	Low
Wilson et al	2021	Yes	Yes	Yes	Yes	Yes	No	Can't tell	3	Low	Low
