# Supplementary material for: Access to primary care for children and young people (CYP) in the UK: a scoping review of CYP’s, caregivers’ and healthcare professionals’ views and experiences of facilitators and barriers
Source: BMJ Open. 2024 May 30;14(5):e081620. doi: 10.1136/bmjopen-2023-081620 (PMC11141190; doi:10.1136/bmjopen-2023-081620)
Supplement: Supplementary data [file bmjopen-2023-081620supp004.pdf]

Additional file 4: data synthesis

Data were synthesised using framework analysis (Gale et al, 2013) in Microsoft Excel. There were four key stages in the analysis process: 1) framework analysis 1 – a descriptive extraction and categorisation, 2) framework analysis 2 – a conceptual analysis, 3) refinement of the themes, and 4) mapping the barriers for sub-populations.

1. Framework analysis 1: a descriptive extraction and categorisation of the data

One reviewer (LH) carried out inductive coding and created an initial framework (a structured template) to summarise/reduce the data to focus on facilitators/barriers to primary healthcare. Data was extracted into an excel worksheet, with each study a row and column a code. The framework was revised iteratively as data from each study was added; by the tenth study, most of the codes were identified and remained the same. The codes were organised under six overarching descriptive categories: accessibility (1), health care beliefs/knowledge/preferences of caregivers (2) and CYP (3), relationship with HCP (4), quality of diagnosis/treatment (5), HCP knowledge/skills/networks/priorities (6). Data from the remaining studies was extracted, with new codes added or revised as best fit the data. Two reviewers (LH and EA) then examined the codes for each category independently and discussed emerging themes.

Table 1: Initial data analysis framework and codes

Overarching category	Codes
**Accessibility**	Multiple opportunities to engage with HCP
	Co-location
	Accessible premises and opening times
	Having information in native or accessible language
	Availability of informational or health resources
	Communication about entry to the service
	Difficulties meeting system structures and requirements
	Difficulties meeting threshold for other services
	Variation/inconsistency of entry criteria
	Patient residential impermanence
	Different ways of being able to access HCP
	Wait times to be seen by HCP
	Reduced services
	Free health care.
	Stigma
	Discrimination
**Parents' beliefs, knowledge, or preferences**	Parents' perceiving access important for child's health
	Familiarity with condition
	Stigma around mental health
	Valuing health professionals' expertise
	Lack of trust in medication/services in UK
	Parents', families or communities' perceiving they had sufficient knowledge themselves
	Parent knowledge or confidence about services or how to access them
	Being able to arrange an emergency appointment
	Parent difficulties attending due to other responsibilities e.g. childcare

	Cultural expectations of health professional
	Concerns about wasting GP time
	Parents feeling misunderstood and not listened to by health system
	Parental perception of gender norms
	Parents feeling shame/judgement
**YPs' beliefs, knowledge, or preferences**	YP knowledge about services or how to access them
	Knowledge/familiarity of condition
	HCP same/opposite sex/gender preferences
	YP anxiety about seeking help from GP or from dentist
	YP perceiving whether HCP would take them seriously, without judgement and be interested in them
	Having choice
	Self-testing
	YP Cultural Expectations of HCP
	YP Self Reliance
	YP Sensitivity relating to family context
**Relationship with HCP**	Clear communication from health professional
	Health professional being friendly, approachable and reassuring (or not)/Personable Qualities of HCP
	Continuity of care
	Trusted relationship
	Confidentiality
	Duration of time in the consultation to listen to concerns
	Parents attending with YP
**Quality of diagnosis or treatment**	Accuracy of test result
	Timeliness of test result
**HCP knowledge, skills, networks, and priorities**	Health professionals having appropriate education and training
	Being able to undertake a good holistic assessment of family needs
	Health professionals' having local knowledge
	Health professionals having signposting, referral and co-ordination skills.
	Collaboration (or lack of) between services
	Priority given to health topic by professional
	HCP Professional Perceptions of Health Topic
	HCP perceptions of the individual

2. Framework analysis 2: a conceptual analysis and development of themes

After reflection and discussion between the reviewers, the data was re-organised conceptually following the journey of a caregiver or CYP from first noticing a health issue and deciding to seek help to attending a consultation and potentially being referred to secondary or adult services, influenced by the work of Ford et al's (2016). Ford et al outlined the following steps in access to primary care for socioeconomically disadvantage older people in rural areas: problem identified, decision to seek help, actively seek help, obtain appointment, get to the appointment, primary care interaction, and outcome.

Five higher-order themes were constructed from the data (see table 2). The data in the initial framework was re-organised, putting data relating to the new a-priori themes into separate Excel

worksheets. Through inductive analysis of the data under each theme, new codes and sub-themes were constructed.

Table 2: Initial higher-order themes

Decision to access care
Reaching and entering services
Communication and trust between HCPs, caregivers and CYP
Gaps in HCP knowledge
General practice as a gatekeeper to, or a holding space for, secondary or adult care

To visualise whether any codes and themes were particularly pertinent for specific sub-populations with higher health needs, data was colour-coded: CYP from deprived areas, looked after children, non-White British CYP, CYP with SEN or disabilities, CYP with chronic conditions, and CYP with mental health problems. Where a study looked at two groups, text was coded in one colour and the cell background another. The sub-populations were selected from CYP target populations and focus clinical areas in the 'Core20Plus5', the national NHS England approach to support the reduction of health inequalities, though we included evidence for any chronic condition instead of the strategy's focus on asthma, diabetes, and epilepsy.

3. Refinement of the themes

The themes and sub-themes were mapped out visually in Powerpoint and discussed with the wider team. The decision to access care, reaching and entering services, and communication and trust formed a repeatable pattern of experiences that affected access to primary care. A consensus was reached among the team that the three sub-themes under "General practice as a gatekeeper…" fit within "Reaching and entering services" and "Communication and trust", and gaps in HCP knowledge impacted on communication and trust, and could be subsumed within that theme. These changes were made and final three over-arching themes were constructed.

4. Mapping the barriers for sub-populations of CYP with higher health needs

Sub-themes that were reported particularly for key sub-populations of interest (see table 3) were systematically mapped into a table.

Table 3: sub-populations of interest

**Author (year)**	CYP with mental health problems	CYP from deprived areas	CYP from non-White British communities	CYP with SEND	CYP with chronic health conditions	Looked after children
Ahmaro et al (2021)						
Alexakis et al (2015)			✓		✓	
Appleton et al (2022)	✓					
Bosley et al (2021)						
Brigham et al (2012)						
Coleman-Fountain et al (2020)	✓			✓		
Condon et al (2020)			✓			
Corry and Leavey (2017)	✓					
Coyle et al (2013)				✓		
Crocker et al (2013)						

Crouch et al (2019)	✓					
Dando et al (2019)			✓			
Davey et al (2013)						
Dickson (2015)		✓				
Diwakar et al (2019)					✓	
Eskytė et al (2021)		✓				
Fox et al (2017)		✓		✓		
Fox et al (2015)	✓					
French et al (2020)				✓		
Henderson and Rubin (2014)		✓				
Ingram et al (2013)						
Jobanputra and Singh (2020)	✓					
Jones et al (2017)						
Lewney et al (2019)						
McDonagh et al (2020)						
Mughal et al (2021)	✓					
Muirhead et al (2017)						✓
Neill et al (2016)*		✓	✓			
Neill et al (2015)*		✓	✓			
O'Brien et al (2019)	✓					
O'Brien et al (2017)	✓					
Ochieng (2020)			✓			
Rapley et al (2021)					✓	
Rashed et al (2022)						
Redsell et al (2013)						
Rickett et al (2021)						
Roberts et al (2014)	✓	✓				
Roberts and Condon (2014)						
Salaheddin and Mason (2016)	✓					
Satherley et al (2021)		✓				
Turnbull et al (2021)						
Turner et al (2012)						
Usher-Smith et al (2015)					✓	
Williams et al (2014)						✓
Williams et al (2012)			✓			
Wilson et al (2021)				✓		
Yassaee et al (2017)						
