# Supplementary material for: Access to primary care for children and young people (CYP) in the UK: a scoping review of CYP’s, caregivers’ and healthcare professionals’ views and experiences of facilitators and barriers
Source: BMJ Open. 2024 May 30;14(5):e081620. doi: 10.1136/bmjopen-2023-081620 (PMC11141190; doi:10.1136/bmjopen-2023-081620)
Supplement: Supplementary data [file bmjopen-2023-081620supp006.pdf]

Supplementary tables

Table 1: Characteristics of studies included

**Author (year)** ***sample location***	***Primary healthcare setting:*** **main focus of study**	**Design**	**Sample population**	**Sample size**	**Focused on CYP in the following age bands:**			**Quality rating**	**Relevance rating**
					**<5**	**5–15**	**16–25**	**High/med/low**	**High/med/low**
Ahmaro *et al.* (2021) *England*	*Pharmacies*: perceptions of YP about sexual health and chlamydia testing and chlamydia treatment.	Qualitative; interviews	YP	26			✓	High	High
Alexakis *et al.* (2015) *England*	*General practice:* understanding the specific issues and service needs of YP with inflammatory bowel disease from black and ethnic minority communities.	Qualitative; interviews	YP	20			✓	High	High
Appleton *et al.* (2022) *England*	*General practice:* exploring the experiences and views of CYP and caregivers of CYP receiving primary care support after child and adolescent mental health services.	Qualitative; interviews	YP and caregivers	14 YP and 13 parents			✓	High	High
Bosley *et al.* (2021) *England*	*General practice and health visiting*: mothers' views on the accessibility and expertise of healthcare professionals caring for their child's health.	Qualitative; focus groups and interviews	Caregivers	6 focus groups (16 parents) and 14 interviews	✓			Medium	Medium
Brigham *et al.* (2012) *England*	*Health visiting*: health visitors' (HVs) perceptions of their role and skills, how they share expertise, and work with other agencies.	Qualitative; focus groups	HCPs	4 focus groups (32 HVs)	✓			Low	Low
Coleman-Fountain *et al.* (2020) *n/k*	*General practice:* exploring how autistic young adults understand and manage mental health problems.	Qualitative; interviews	YP	19			✓	High	Low
Condon *et al.* (2020) *England*	*General practice and health visiting*: parents' experiences of using child health services for their children post-migration from Romania, Poland, Pakistan or Somalia.	Qualitative; focus groups	Caregivers	Five focus groups (28 parents)	✓			High	Low

Corry and Leavey (2017) *Northern Ireland*	*General practice*: adolescents' attitudes to consulting their GP about psychological problems.	Qualitative; focus groups	YP	Nine focus groups (54 YP)		✓	✓	High	High
Coyle *et al.* (2013) *Northern Ireland and Scotland*	*Dental care*: investigated practitioners' willingness to treat adolescents with learning disabilities (LD) in primary dental care.	Quantitative; survey	HCPs	300		✓	✓	Low	Low
Crocker *et al.* (2013) *Wales*	*General practice:* identifying differences between children who consulted a GP and those who did not before the day of hospital presentation with pneumonia or empyema.	Mixed methods; survey and structured interviews	Caregivers	151 survey participants of whom 79 were interviewed	✓	✓		Low	Low
Crouch *et al.* (2019) *England*	*General practice:* understanding families' experiences of seeking help and accessing specialist treatment for childhood anxiety	Qualitative; interviews	Caregivers	16		✓		High	Medium
Dando *et al.* (2019) *England*	*General practice:* understanding the healthcare experiences of Albanian survivors of modern slavery and sexual exploitation	Qualitative; interviews	Caregivers	7 participants of whom 6 were caregivers	✓	n/k		Low	Low
Davey *et al.* (2013) *England*	*General practice and walk-in centres*: explored the needs and experiences of young adults of primary healthcare services.	Qualitative; interviews	YP	20			✓	Medium	High
Dickson (2015) *Northern Ireland*	*Dental care*: parents' perceptions of factors influencing dental registrations of children living within a Sure Start area.	Qualitative; interviews	Caregivers	8	✓			Low	Medium
Diwakar *et al.* (2019) *England*	*General practice:* understanding parent experiences with paediatric allergy pathways.	Qualitative; interviews	Caregivers	18	✓	✓		Medium	Medium
Eskytė *et al.* (2021) *England*	*Health visiting*: organisational factors that obstruct HVs from speaking to parents of babies about oral health	Qualitative; interviews and focus groups	HCPs	3 focus groups (15 HVs) 3 interviews	✓			High	Low

Fox *et al.* (2017) *England*	*General practice and health visiting*: assessed what families affected by autism need and how health, education, and social care services can support them.	Qualitative; interviews	Caregivers	15	✓	✓		High	High
Fox *et al.* (2015) *England*	*General practice*: GP's capabilities, motivations and opportunities for discussing self-harm and to identify barriers to and enablers for discussing self-harm with YP.	Mixed methods; online survey and interviews	HCPs	28 (online survey) 10 (interviews)		✓	✓	Medium	Medium
French *et al.* (2020) *UK*	*General practice:* exploring the primary care experiences of referral and management of ADHD	Qualitative; interviews	HCPs, adults with ADHD, caregivers	5 primary HCP, 5 adults with ADHD, 5 caregivers, 5 secondary HCP	n/k	n/k	n/k	Medium	High
Henderson and Rubin (2014) *England*	*Dental care*: dental, school and family perspectives of an oral health promotion initiative to improve access for pre-school children in deprived communities.	Qualitative; focus groups and interviews	HCPs, school staff, caregivers and CYP	6 focus groups (24 dental practitioners), 9 interviews (school staff), 4 interviews (caregivers and their child)	✓	✓		Low	Low
Ingram *et al.* (2013) *n/k*	*General practice:* to explore parents' views on support and information needs prior to consulting when children have respiratory tract infections with a cough	Qualitative; focus groups and interviews	Caregivers	60	✓	✓		Medium	High
Jobanputra and Singh (2020) *England*	*General practice:* exploring GPs' views on the management of adolescents with mental health disorders	Qualitative; interviews	HCPs	8		✓	✓	Low	Medium
Jones *et al.* (2017) *England*	*General practice*: young adults' opinions of receiving chlamydia testing with condom provision, contraceptive information, and HIV testing.	Qualitative; interviews	YP	30			✓	High	High

Lewney *et al.* (2019) *England*	*Health visiting:* exploring how HVs feel about providing oral health advice and dealing with dental issues	Qualitative; interviews	HCPs	17	✓			High	Medium
McDonagh *et al*. (2020) *UK*	*General practice*: YPs' perspectives on barriers to chlamydia testing and potential intervention functions and implementation strategies to overcome identified barriers.	Qualitative; interviews	YP	28			✓	Medium	High
Mughal *et al.* (2021) *England*	*General practice*: the help-seeking behaviours, experiences of GP care, and access to the general practice of YP who self-harm.	Qualitative; interviews	YP	13			✓	Medium	High
Muirhead *et al.* (2017) *England*	*Dental care:* to understand foster carers' oral health knowledge, attitudes, and experiences of managing foster children's oral health.	Qualitative; focus groups	Caregivers	12		✓	✓	Medium	Low
Neill *et al.* (2016)***** *England*	*Primary care (all except dental and optometry)*: how parents from different socio-economic groups use the information to make decisions during acute childhood illness at home.	Qualitative; focus groups and interviews	Caregivers	Five focus groups (24 parents) and 3 interviews	✓			High	High
Neill *et al.* (2015)***** *England*	*Primary care (all except dental and optometry)*: parents' use of information resources during decision-making in acute childhood illness at home.	Qualitative; focus groups and interviews	Caregivers	Five focus groups (24 parents) and 3 interviews	✓			High	High
O'Brien *et al.* (2019) *England*	*General practice*: GPs experiences of barriers to and facilitators of identifying, managing, and accessing specialist services for anxiety disorders.	Quantitative; cross-sectional survey	HCPs	971	✓	✓		High	Low
O'Brien *et al.* (2017) *England*	*General practice:* explore the experiences of GPs in identification, management, and access to specialist services for anxiety disorders.	Qualitative; interviews	HCPs	20	✓	✓		High	High
Ochieng (2020) *England*	*Health visiting*: the sociocultural, family, and environmental factors that either influence healthy weight in black African children.	Qualitative; focus groups	Caregivers and HCPs	4 focus groups (30 parents) and 3 focus groups (32 HVs)	✓	✓		High	Low

Rapley *et al.* (2021) *England*	*Primary care (all except optometry):* exploring the experiences of care, from initial symptoms to initial referral to paediatric rheumatology.	Qualitative; interviews	Caregivers and HCPs	51 interviews with caregivers (related to 36 CYP), 11 interviews with HCPs	✓	✓	✓	High	High
Rashed *et al.* (2022) *England*	*Pharmacy and general practice:* exploring the experiences, barriers and recommendations of caregivers and YP regarding the use of community pharmacies for children.	Mixed; survey with closed and open questions	Caregivers and YP	213 caregivers and 20 YP	✓	✓	✓	Low	Medium
Redsell *et al.* (2013) *England*	*Health visiting*: investigated the beliefs and current practices of UK HVs concerning recognising and intervening with infants at risk of developing obesity.	Qualitative; interviews	HCPs	30	✓			Low	Low
Rickett *et al.* (2021) *Scotland, Wales, and England*	*General practice:* to understand the healthcare expectations and experiences of caregivers seeking support for their gender diverse children	Mixed; survey with closed and open questions	Caregivers	75	✓	✓		Medium	High
Roberts *et al.* (2014) *England*	*General practice*: GPs' experiences and perceptions of consulting with adolescents who present with psychological difficulties.	Qualitative; interviews	HCPs	19		✓	✓	Low	High
Roberts and Condon (2014) *England*	*Dental care:* exploring parental attitudes to pre-school oral health.	Qualitative; interviews	Caregivers	12	✓			Low	Low
Salaheddin and Mason (2016) *UK*	*General practice:* exploring the barriers to accessing mental health support among young adults.	Mixed; survey with closed and open questions	YP	203			✓	Low	Low
Satherley *et al.* (2021) *England*	*General practice*: how mothers living in deprived neighbourhoods support their children with health conditions.	Qualitative; interviews	Caregivers	8	✓	✓		High	High
Turnbull *et al.* (2021) *England*	*Pharmacy and sexual health clinic:* young women's experiences of accessing emergency contraception pills from pharmacies and sexual health clinics.	Qualitative; interviews	YP	21			✓	Medium	High

Turner *et al.* (2012) *England*	*General practice*: exploring parents' views and experiences of primary care as a treatment setting for childhood obesity.	Qualitative; interviews	Caregivers	15		✓		Low	High
Usher-Smith *et al*. (2015) *England*	*General practice and secondary care*: Explored the pathway to diagnosis of type 1 diabetes.	Quantitative; survey	Caregivers	87	✓	✓		Medium	Medium
Williams *et al.* (2014) *England and Wales*	*Dental care*: the impact of a community-based dental care pathway on children's dental care entering residential or foster care.	Qualitative; interviews and routinely collected data	HCPs, social workers, CYP, and caregivers	Routinely collected data on 89 CYP<br>Dental health professionals (n=6)<br>Social workers (n=2)<br>CYP (n=3)<br>Caregivers (n=5)	✓	✓	✓	Low	Low
Williams *et al.* (2012) *England*	*Not specified (preventative primary care services)*: Described African and African-Caribbean fathers' beliefs about fatherhood, health and preventive primary care services.	Qualitative; focus groups	Caregivers	9 focus groups (46 parents)	n/k	n/k	n/k	Medium	Medium
Wilson *et al.* (2021) *England*	*Optometric practices*: accessibility of eye care for children with typical development and those with autism.	Quantitative; telephone survey	Optometric practices	400	✓	✓		Low	Low
Yassaee *et al.* (2017) *England*	*General practice*: adolescents' experiences of their GP, whether poor reported GP experience was associated with worse physical and mental health measures and whether poor previous GP experience was linked to lower service utilisation.	Quantitative; cross-sectional survey	YP	5,335		✓		High	Low

Table 2: Health topic and CYP population studied

Health topic (e.g., dental, sexual)	Specific population	Primary healthcare setting	Citations
**Non-specific health condition (n=13)**	• Non-specific (n=5)<br>• African and African-Caribbean fathers (n=1)<br>• CYP from different socio-economic and ethnic groups (n=1)<br>• CYP living in deprived areas (n=1)<br>• CYP of Albanian survivors of modern slavery and sexual exploitation (n=1)<br>• CYP migrants from Romania, Poland, Pakistan, or Somalia (n=1)<br>• CYP with ADHD (n=1)<br>• CYP with autism from an ethnic minority/migrant community (n=1)<br>• South Asian and Gypsy/Travelling communities (n=1)	• General practice (n=4)<br>• General practice and health visiting (n=3)<br>• Primary care (all except dentist, n=2)<br>• General practice and walk-in centres (n=1)<br>• Health visiting (n=1)<br>• General practice and pharmacy (n=1)<br>• Not specified (preventative primary care services, n=1)	Condon et al (2020); Brigham et al (2012); Bosley et al. (2021); Dando et al. (2019); Davey et al. (2013); French et al. (2020); Fox et al. (2017); Neill et al. (2015); Neill et al. (2016); Rashed et al. (2022); Satherley et al. (2021); Williams et al. (2012); Yassaee et al. (2017)
**Mental health (n=11)**	• Non-specific (n=9)<br>• CYP living in deprived areas (n=1)<br>• CYP with autism (n=1)	• General practice (n=11)	Appleton et al. (2022); Coleman-Fountain et al. (2020); Corry and Leavey (2017); Crouch et al. (2019); Fox et al. (2015); Jobanputra and Singh (2020); Mughal et al. (2021); O'Brien et al. (2017); O'Brien et al. (2019); Roberts et al. (2014); Salaheddin and Mason (2016)
**Gender diversity (n=1)**	• Non-specific (n=1)	• General practice (n=1)	Rickett et al. (2021)
**Chronic conditions (n=4) (allergies, n=1; inflammatory bowel disease, n=1; juvenile idiopathic arthritis, n=1; type 1 diabetes, n=1)**	• Non-specific (n=3)<br>• Black and minority ethnic CYP (n=1)	• General practice (n=3)<br>• Primary care (all except optometry) (n=1)	Alexakis et al. (2015); Diwakar et al. (2019); Rapley et al. (2021); Usher-Smith et al. (2015)

**Physical health (n=4) (obesity, n=3; pneumonia or empyema, n=1; respiratory tract infections, n=1)**	• Non-specific (n=3)<br>• Black African CYP (n=1)	• General practice (n=2)<br>• Health visiting (n=2)	Crocker et al. (2013); Ingram et al. (2013); Ochieng (2020); Redsell et al. (2013); Turner et al. (2012)
**Oral health (n=8)**	• Non-specific (n=2)<br>• CYP in care (n=2)<br>• CYP living in deprived areas (n=3)<br>• CYP with learning disabilities (n=1)	• Dental care (n=6)<br>• Health visiting (n=2)	Coyle et al. (2013); Dickson (2015); Eskyte et al. (2021); Henderson and Rubin (2014); Lewney et al. (2019); Muirhead et al. (2017); Roberts and Condon (2014); Williams et al. (2014)
**Sexual health (n=4)**	• Non-specific (n=4)	• General practice (n=2)<br>• Pharmacy (n=1)<br>• Pharmacy and sexual health clinic (n=1)	Ahmaro et al. (2021); Jones et al. (2017); McDonagh et al. (2020); Turnbull et al. (2021)
**Optometry (n=1)**	• Younger CYP and CYP with autism (n=1)	• Optometric practices (n=1)	Wilson et al. (2021)
