# Supplementary material for: Access to primary care for children and young people (CYP) in the UK: a scoping review of CYP’s, caregivers’ and healthcare professionals’ views and experiences of facilitators and barriers
Source: BMJ Open. 2024 May 30;14(5):e081620. doi: 10.1136/bmjopen-2023-081620 (PMC11141190; doi:10.1136/bmjopen-2023-081620)
Supplement: Author's manuscript [file bmjopen-2023-081620.draft_revisions.pdf]

BMJ Open is committed to open peer review. As part of this commitment we make the peer review history of every article we publish publicly available.

When an article is published we post the peer reviewers' comments and the authors' responses online. We also post the versions of the paper that were used during peer review. These are the versions that the peer review comments apply to.

The versions of the paper that follow are the versions that were submitted during the peer review process. They are not the versions of record or the final published versions. They should not be cited or distributed as the published version of this manuscript.

BMJ Open is an open access journal and the full, final, typeset and author-corrected version of record of the manuscript is available on our site with no access controls, subscription charges or pay-per-view fees (http://bmjopen.bmj.com).

If you have any questions on BMJ Open's open peer review process please email info.bmjopen@bmj.com

BMJ Open

Access to primary care for children and young people (CYP) in the UK: a scoping review of CYP's, caregivers', and healthcare professionals' views and experiences of facilitators and barriers

Journal:	*BMJ Open*
Manuscript ID	bmjopen-2023-081620
Article Type:	Original research
Date Submitted by the Author:	02-Nov-2023

Complete List of Authors:	Herlitz, Lauren; UCL GOS Institute of Child Health, NIHR Children and Families Policy Research Unit, Population, Policy and Practice<br>Ashford, Emily; UCL Social Research Institute, Thomas Coram Research Unit<br>Powell, Claire; UCL GOS Institute of Child Health, NIHR Children and Families Policy Research Unit, Population, Policy and Practice<br>Herbert, Kevin; Cambridge University, Public Health and Primary Care; Cambridge Research Methods Hub,<br>Morris, Stephen; University of Cambridge, Primary Care Unit<br>Woodman, Jenny; UCL Social Research Institute, Thomas Coram Research Unit

Keywords:	Primary Care < Primary Health Care, Adolescents < Adolescent, Health Equity, PAEDIATRICS, Health Services Accessibility

SCHOLARONE™
Manuscripts

I, the Submitting Author has the right to grant and does grant on behalf of all authors of the Work (as defined in the below author licence), an exclusive licence and/or a non-exclusive licence for contributions from authors who are: i) UK Crown employees; ii) where BMJ has agreed a CC-BY licence shall apply, and/or iii) in accordance with the terms applicable for US Federal Government officers or employees acting as part of their official duties; on a worldwide, perpetual, irrevocable, royalty-free basis to BMJ Publishing Group Ltd ("BMJ") its licensees and where the relevant Journal is co-owned by BMJ to the co-owners of the Journal, to publish the Work in this journal and any other BMJ products and to exploit all rights, as set out in our licence.

The Submitting Author accepts and understands that any supply made under these terms is made by BMJ to the Submitting Author unless you are acting as an employee on behalf of your employer or a postgraduate student of an affiliated institution which is paying any applicable article publishing charge ("APC") for Open Access articles. Where the Submitting Author wishes to make the Work available on an Open Access basis (and intends to pay the relevant APC), the terms of reuse of such Open Access shall be governed by a Creative Commons licence – details of these licences and which Creative Commons licence will apply to this Work are set out in our licence referred to above.

Other than as permitted in any relevant BMJ Author's Self Archiving Policies, I confirm this Work has not been accepted for publication elsewhere, is not being considered for publication elsewhere and does not duplicate material already published. I confirm all authors consent to publication of this Work and authorise the granting of this licence.

For peer review only

Access to primary care for children and young people (CYP) in the UK: a scoping review of CYP's, caregivers', and healthcare professionals' views and experiences of facilitators and barriers

Lauren Herlitz*, NIHR Children and Families Policy Research Unit**,** Population, Policy and Practice, UCL Great Ormond Street Institute of Child Health, 30 Guilford Street, London, WC1N 1EH, UK. ORCID: 0000-0003-2497-9041

Emily Ashford, Thomas Coram Research Unit, UCL Social Research Institute, 27 Woburn Square, London, UK. ORCID: 0000-0001-7193-4378

Claire Powell, NIHR Children and Families Policy Research Unit**,** Population, Policy and Practice, UCL Great Ormond Street Institute of Child Health, 30 Guilford Street, London, WC1N 1EH, UK. ORCID: 0000-0002-6581-0165

Kevin Herbert, Primary Care Unit, Department of Public Health & Primary Care, University of Cambridge, Cambridge, UK. ORCID: 0009-0008-4354-7811

Steve Morris, Primary Care Unit, Department of Public Health & Primary Care, University of Cambridge, Cambridge, UK. ORCID: 0000-0002-5828-3563

Jenny Woodman, Thomas Coram Research Unit, UCL Social Research Institute, 27 Woburn Square, London, UK. ORCID: 0000-0002-9403-4177

*Corresponding author: l.herlitz@ucl.ac.uk

For peer review only - http://bmjopen.bmj.com/site/about/guidelines.xhtml

Abstract (max 300 words)

Objectives

To examine children and young people's (CYP), caregivers' and healthcare professionals' (HCP) views or experiences of facilitators and barriers to CYP access to UK primary care services to better understand healthcare inequity. To explore differences across CYP sub-populations with greater health needs: from deprived areas, identifying as ethnic minorities, with experiences of state care, special educational needs or disabilities, chronic conditions, or mental health problems.

Design

Scoping review.

Eligibility criteria

Included studies were in English, published 2012 – 2022 and reported: the views/experiences of CYP (0 – 25 years), caregivers, or HCPs about accessing UK primary care; using quantitative or qualitative empirical methods.

Data sources

Pubmed, CINAHL, Web of Science, Psycinfo and Scopus.

Results

We included 47 reports (46 studies). CYP/caregivers' decision to access care was facilitated by CYP/caregivers' or their family/friends' ability to identify a health issue as warranting healthcare attention. Barriers to accessing care included perceived stigma (e.g., being seen as a bad parent), embarrassment, and discrimination experiences. CYP and caregivers believed longer opening hours could facilitate more timely access to care. Caregivers and HCPs reported that delayed or rejected referrals to secondary or adult care was a barrier to having needs met, especially for CYP with poor mental health. CYP and caregivers in numerous studies emphasised the importance of communication and trust with HCPs, including taking their concerns seriously, being knowledgeable, and providing continuity of care for CYP. Common barriers reported across high-need sub-populations were caregivers needing knowledge and confidence to advocate for their child, gaps in HCP's knowledge, and a lack of connectedness between primary and secondary care.

Conclusions

Connecting general practices and community health workers/services, improving CYP/caregivers' understanding of common childhood conditions, addressing HCP's knowledge gaps in paediatric care, and integrated approaches between primary and secondary care may reduce inequity in access.

Strengths and limitations of this study

- The review was rigorously conducted and included quality appraisal.
- Mapping patterns of facilitators/barriers across different sub-populations with higher health needs was a strength of the review, revealing that access was affected by caregivers having to be

For peer review only - http://bmjopen.bmj.com/site/about/guidelines.xhtml

able to confidently advocate for their child's needs, multiple barriers existed for some groups, and there was a lack of evidence on access for looked after children.

- Studies in systematic reviews were not screened and we did not search for grey literature due to time and resources constraints.
- Supply barriers, e.g. recruiting and retaining GPs, which affect both CYP and adult patients were not identified using our search terms.

Key words

Primary care; adolescents; children; access to healthcare; health equity

Introduction

Access to healthcare can be defined as the opportunity to identify healthcare needs, to seek, reach and use healthcare services, and to have healthcare needs met (1,2). Primary care access in childhood is important to ensure that children and young people (CYP) are vaccinated, reach developmental milestones, are safeguarded, and that acute and chronic conditions are identified and managed (3,4). Evidence also suggests improved access to primary care may reduce the escalation of health concerns, alleviating pressure on secondary care (5–7). The National Health Service's (NHS) long term plan in England highlights the role of primary care in reducing health inequalities and ensuring CYP have a strong start in life, in particular improving access for CYP with mental health problems, learning disabilities or autism (8). Unmet healthcare needs in adolescence are an independent predictor of poor adult health (9,10).

Recent evidence suggests that CYP access to primary care is inequitable. For example, UK cohort studies linked to routine health data found that CYP living in deprived areas were less likely to access primary care relative to their wealthier peers, and more likely to use acute care (11–13). Inequalities in CYP access to care may result from: variation in the supply of healthcare by area deprivation (14); differences in how conditions are identified and managed, for example, because of increased multimorbidity in CYP in deprived areas (15), or variation in healthcare professionals' (HCPs) expertise (16). Access may be affected by differences in CYP or their caregiver's needs and preferences. While systematic reviews have been conducted on CYP and HCP's views of some specific services in the UK (17–19), this study aimed to synthesise perspectives of CYP, caregivers, and HCP across primary care services in order to deepen understanding of healthcare inequity, barriers to healthcare and how to address them, and looked in detail at facilitators and barriers for CYP with high health needs.

Method

Our methods were informed by rapid evidence review guidance (20). We pre-registered the review protocol in the Open Science Framework (https://osf.io/mfc3z). The study followed the Preferred Reporting Items for Systematic Reviews and Meta-Analyses extension for Scoping Reviews (PRISMA-ScR) statement (additional file 1).

Inclusion/exclusion criteria

We included a study if it:

For peer review only - http://bmjopen.bmj.com/site/about/guidelines.xhtml

- Reported the views or experiences of CYP (aged 0 to 25 years), caregiver (i.e., parent or carer), or HCPs on the facilitators and barriers to primary care access, including studies that examined primary care as a means of accessing secondary care.
- Was based in the UK.
- Used quantitative or qualitative empirical methods.
- Was published in English between 2012 and 2022.

We excluded studies that focused on access to school health services, access to primary care during the COVID-19 pandemic, or on the uptake of vaccinations/immunisations.

Search strategy

We searched PubMed, CINAHL, Web of Science (Social Sciences Citation Index), Psycinfo and Scopus using free-text and index terms for the following concepts: healthcare access, primary care, CYP, UK, and facilitator and barriers (see additional file 2).

Document selection

We imported the search results into Rayyan software (https://www.rayyan.ai/) for de-duplication and screening. Five reviewers independently conducted title/abstract screening and twenty per cent (N=1334) were checked by a second reviewer. Two reviewers independently conducted full-text screening and 25% (N=36) were checked by a second reviewer. The first and second reviewers discussed disagreements until a consensus was reached, bringing in a third team member where necessary.

Data extraction

The following data were extracted: study sample/population; primary care setting; area of health care; study design/methodology; factors affecting primary care access. Data on access to primary care during the COVID-19 pandemic were not extracted.

Quality appraisal

Five reviewers assessed study quality using the Mixed Methods Appraisal Tool (21,22). No study was excluded based on quality, but study quality is acknowledged in the findings and quotes presented are from medium- and high-quality studies only. One reviewer (LH) assigned studies two 'weight-of-evidence' ratings (23), one for quality and one for relevance to answering the review question, rated 'low', 'medium' or 'high' (see additional file 3). For a judgement of 'high' relevance, studies had to describe, with breadth and depth, factors influencing primary care access and privilege participants' perspectives.

Data synthesis

Data were synthesised using framework analysis (24) to systematically review and map the data from each study using a structured template (see additional file 4). After data were descriptively coded, a conceptual framework was applied following a patient pathway from a CYP/caregiver identifying a health issue and deciding to seek help, to organising an appointment, and attending a consultation, influenced by previous work (25). To visualise whether any codes and themes were pertinent for specific sub-populations with high needs, data were colour-coded for the following CYP groups: from deprived areas, experiences of state care (i.e. looked after children), identifying as

For peer review only - http://bmjopen.bmj.com/site/about/guidelines.xhtml

ethnic minorities, with SEN or disabilities, with chronic conditions, and with mental health problems. Sub-populations were selected from CYP target populations and focus clinical areas in the 'Core20Plus5', the NHS England strategy for reducing health inequalities (26). Sub-themes reported for these sub-populations were systematically mapped.

Results

Of the 6,671 unique title/abstracts were generated from database searching in February 2022, 47 reports (of 46 studies) met the inclusion criteria (see figure 1).

Study characteristics

Study design/methods

Most studies were qualitative using interviews (n=25), or focus groups (n=6), or focus groups and interviews (n=5). All quantitative studies used cross-sectional surveys (n=5), whilst mixed-method studies used surveys that contained open and closed questions (n=5) (see table 1).

Health topic

Thirteen studies (28%) were related to CYP access for non-specific health conditions; 11 (24%) were about CYP with mental health conditions; 8 (17%) were about CYP's oral health; 4 (9%) focused on CYP with chronic health conditions; 4 (9%) were about CYP with physical health conditions; 4 (9%) focused on young people's (YP's) sexual health; 1 (2%) was on help-seeking for children's gender identity; and 1 (2%) examined CYP eye care from optometry practices (see supplementary table).

Study participants

Most studies invited either caregivers (n=18), young people (aged 11+ years) (n=11), or HCPs (n=10) to participate; seven studies included more than one type of participant and one study surveyed optometry practices. More than half of studies focused on CYP in general (n=28); the rest focused on a particular sub-population(s) (see table 2).

Primary healthcare setting

The following healthcare settings were studied (note, several studies covered multiple settings): general practice (n=27), health visiting (n=8), dental care (n=6), overall primary care (excluding dental care or optometry) (n=4), pharmacy services (n=3), optometry (n=1), walk-in centres (n=1) or sexual health clinics (n=1) (see table 2).

Study quality and relevance

Ten studies (22%) were rated high on both quality and relevance (see additional file 3). Studies on CYP with chronic conditions and sexual health were rated higher on quality and relevance; while half of oral health studies, and the only optometry study, were rated low on quality.

For peer review only

Figure 1: PRISMA Flow Diagram

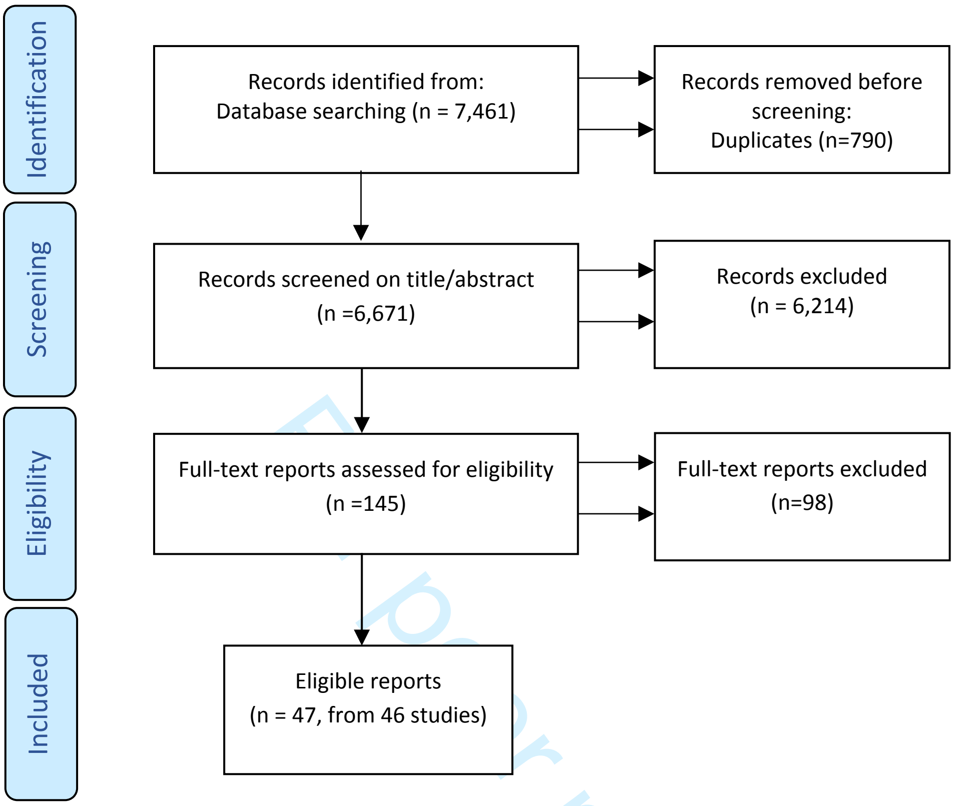

For peer review only

For peer review only - http://bmjopen.bmj.com/site/about/guidelines.xhtml

Table 1: Characteristics of studies included

Author (year) (citation) ***sample location***	***Primary healthcare setting:*** **main focus of study**	**Design**	**Sample population**	**Sample size**	**Focused on CYP in the following age bands:**			**Quality rating**	**Relevance rating**
					**<5**	**5–15**	**16–25**	**High/med/ low**	**High/med/ low**
Ahmaro *et al.* (2021) (27) *England*	*Pharmacies*: perceptions of YP about sexual health and chlamydia testing and chlamydia treatment.	Qualitative; interviews	YP	26			✓	High	High
Alexakis *et al.* (2015) (28) *England*	*General practice:* understanding the specific issues and service needs of YP with inflammatory bowel disease from black and ethnic minority communities.	Qualitative; interviews	YP	20			✓	High	High
Appleton *et al.* (2022) (29) *England*	*General practice:* exploring the experiences and views of CYP and caregivers of CYP receiving primary care support after child and adolescent mental health services.	Qualitative; interviews	YP and caregivers	14 YP and 13 parents			✓	High	High
Bosley *et al.* (2021) (30) *England*	*General practice and health visiting*: mothers' views on the accessibility and expertise of healthcare professionals caring for their child's health.	Qualitative; focus groups and interviews	Caregivers	6 focus groups (16 parents) and 14 interviews	✓			Medium	Medium
Brigham *et al.* (2012) (31) *England*	*Health visiting*: health visitors' (HVs) perceptions of their role and skills, how they share expertise, and work with other agencies.	Qualitative; focus groups	HCPs	4 focus groups (32 HVs)	✓			Low	Low
Coleman-Fountain *et al.* (2020) (32) *n/k*	*General practice:* exploring how autistic young adults understand and manage mental health problems.	Qualitative; interviews	YP	19			✓	High	Low
Condon *et al.* (2020) (33) *England*	*General practice and health visiting*: parents' experiences of using child health services for their children post-migration from Romania, Poland, Pakistan or Somalia.	Qualitative; focus groups	Caregivers	Five focus groups (28 parents)	✓			High	Low

For peer review only - http://bmjopen.bmj.com/site/about/guidelines.xhtml

Corry and Leavey (2017) (34) *Northern Ireland*	*General practice*: adolescents' attitudes to consulting their GP about psychological problems.	Qualitative; focus groups	YP	Nine focus groups (54 YP)		✓	✓	High	High
Coyle *et al.* (2013) (35) *Northern Ireland and Scotland*	*Dental care*: investigated practitioners' willingness to treat adolescents with learning disabilities (LD) in primary dental care.	Quantitative; survey	HCPs	300		✓	✓	Low	Low
Crocker *et al.* (2013) (36) *Wales*	*General practice:* identifying differences between children who consulted a GP and those who did not before the day of hospital presentation with pneumonia or empyema.	Mixed methods; survey and structured interviews	Caregivers	151 survey participants of whom 79 were interviewed	✓	✓		Low	Low
Crouch *et al.* (2019) (37) *England*	*General practice:* understanding families' experiences of seeking help and accessing specialist treatment for childhood anxiety	Qualitative; interviews	Caregivers	16		✓		High	Medium
Dando *et al.* (2019) (38) *England*	*General practice:* understanding the healthcare experiences of Albanian survivors of modern slavery and sexual exploitation	Qualitative; interviews	Caregivers	7 participants of whom 6 were caregivers	✓	n/k		Low	Low
Davey *et al.* (2013) (39) *England*	*General practice and walk-in centres*: explored the needs and experiences of young adults of primary healthcare services.	Qualitative; interviews	YP	20			✓	Medium	High
Dickson (2015) (40) *Northern Ireland*	*Dental care*: parents' perceptions of factors influencing dental registrations of children living within a Sure Start area.	Qualitative; interviews	Caregivers	8	✓			Low	Medium
Diwakar *et al.* (2019) (41) *England*	*General practice:* understanding parent experiences with paediatric allergy pathways.	Qualitative; interviews	Caregivers	18	✓	✓		Medium	Medium
Eskytė *et al.* (2021) (42) *England*	*Health visiting*: organisational factors that obstruct HVs from speaking to parents of babies about oral health	Qualitative; interviews and focus groups	HCPs	3 focus groups (15 HVs) 3 interviews	✓			High	Low

For peer review only - http://bmjopen.bmj.com/site/about/guidelines.xhtml

Fox *et al.* (2017) (43) *England*	*General practice and health visiting*: assessed what families affected by autism need and how health, education, and social care services can support them.	Qualitative; interviews	Caregivers	15	✓	✓		High	High
Fox *et al.* (2015) (44) *England*	*General practice*: GP's capabilities, motivations and opportunities for discussing self-harm and to identify barriers to and enablers for discussing self-harm with YP.	Mixed methods; online survey and interviews	HCPs	28 (online survey) 10 (interviews)		✓	✓	Medium	Medium
French *et al.* (2020) (45) *UK*	*General practice:* exploring the primary care experiences of referral and management of ADHD	Qualitative; interviews	HCPs, adults with ADHD, caregivers	5 primary HCP, 5 adults with ADHD, 5 caregivers, 5 secondary HCP	n/k	n/k	n/k	Medium	High
Henderson and Rubin (2014) (46) *England*	*Dental care*: dental, school and family perspectives of an oral health promotion initiative to improve access for pre-school children in deprived communities.	Qualitative; focus groups and interviews	HCPs, school staff, caregivers and CYP	6 focus groups (24 dental practitioners), 9 interviews (school staff), 4 interviews (caregivers and their child)	✓	✓		Low	Low
Ingram *et al.* (2013) (47) *n/k*	*General practice:* to explore parents' views on support and information needs prior to consulting when children have respiratory tract infections with a cough	Qualitative; focus groups and interviews	Caregivers	60	✓	✓		Medium	High
Jobanputra and Singh (2020) (48) *England*	*General practice:* exploring GPs' views on the management of adolescents with mental health disorders	Qualitative; interviews	HCPs	8		✓	✓	Low	Medium
Jones *et al.* (2017) (49) *England*	*General practice*: young adults' opinions of receiving chlamydia testing with condom provision, contraceptive information, and HIV testing.	Qualitative; interviews	YP	30			✓	High	High

For peer review only - http://bmjopen.bmj.com/site/about/guidelines.xhtml

For peer review only

Lewney *et al.* (2019) (50) *England*	*Health visiting:* exploring how HVs feel about providing oral health advice and dealing with dental issues	Qualitative; interviews	HCPs	17	✓			High	Medium
McDonagh *et al*. (2020) (51) *UK*	*General practice*: YPs' perspectives on barriers to chlamydia testing and potential intervention functions and implementation strategies to overcome identified barriers.	Qualitative; interviews	YP	28			✓	Medium	High
Mughal *et al.* (2021) (52) *England*	*General practice*: the help-seeking behaviours, experiences of GP care, and access to the general practice of YP who self-harm.	Qualitative; interviews	YP	13			✓	Medium	High
Muirhead *et al.* (2017) (53) *England*	*Dental care:* to understand foster carers' oral health knowledge, attitudes, and experiences of managing foster children's oral health.	Qualitative; focus groups	Caregivers	12		✓	✓	Medium	Low
Neill *et al.* (2016)* (54) *England*	*Primary care (all except dental and optometry)*: how parents from different socio-economic groups use the information to make decisions during acute childhood illness at home.	Qualitative; focus groups and interviews	Caregivers	Five focus groups (24 parents) and 3 interviews	✓			High	High
Neill *et al.* (2015)* (55) *England*	*Primary care (all except dental and optometry)*: parents' use of information resources during decision-making in acute childhood illness at home.	Qualitative; focus groups and interviews	Caregivers	Five focus groups (24 parents) and 3 interviews	✓			High	High
O'Brien *et al.* (2019) (56) *England*	*General practice*: GPs experiences of barriers to and facilitators of identifying, managing, and accessing specialist services for anxiety disorders.	Quantitative; cross-sectional survey	HCPs	971	✓	✓		High	Low
O'Brien *et al.* (2017) (57) *England*	*General practice:* explore the experiences of GPs in identification, management, and access to specialist services for anxiety disorders.	Qualitative; interviews	HCPs	20	✓	✓		High	High
Ochieng (2020) (58) *England*	*Health visiting*: the sociocultural, family, and environmental factors that either influence healthy weight in black African children.	Qualitative; focus groups	Caregivers and HCPs	4 focus groups (30 parents) and 3 focus groups (32 HVs)	✓	✓		High	Low

For peer review only

Rapley *et al.* (2021) (59) *England*	*Primary care (all except optometry):* exploring the experiences of care, from initial symptoms to initial referral to paediatric rheumatology.	Qualitative; interviews	Caregivers and HCPs	51 interviews with caregivers (related to 36 CYP), 11 interviews with HCPs	✓	✓	✓	High	High
Rashed *et al.* (2022) (60) *England*	*Pharmacy and general practice:* exploring the experiences, barriers and recommendations of caregivers and YP regarding the use of community pharmacies for children.	Mixed; survey with closed and open questions	Caregivers and YP	213 caregivers and 20 YP	✓	✓	✓	Low	Medium
Redsell *et al.* (2013) (61) *England*	*Health visiting*: investigated the beliefs and current practices of UK HVs concerning recognising and intervening with infants at risk of developing obesity.	Qualitative; interviews	HCPs	30	✓			Low	Low
Rickett *et al.* (2021) (62) *Scotland, Wales, and England*	*General practice:* to understand the healthcare expectations and experiences of caregivers seeking support for their gender diverse children	Mixed; survey with closed and open questions	Caregivers	75	✓	✓		Medium	High
Roberts *et al.* (2014) (63) *England*	*General practice*: GPs' experiences and perceptions of consulting with adolescents who present with psychological difficulties.	Qualitative; interviews	HCPs	19		✓	✓	Low	High
Roberts and Condon (2014) (64) *England*	*Dental care:* exploring parental attitudes to pre-school oral health.	Qualitative; interviews	Caregivers	12	✓			Low	Low
Salaheddin and Mason (2016) (65) *UK*	*General practice:* exploring the barriers to accessing mental health support among young adults.	Mixed; survey with closed and open questions	YP	203			✓	Low	Low
Satherley *et al.* (2021) (66) *England*	*General practice*: how mothers living in deprived neighbourhoods support their children with health conditions.	Qualitative; interviews	Caregivers	8	✓	✓		High	High

For peer review only - http://bmjopen.bmj.com/site/about/guidelines.xhtml

Turnbull *et al.* (2021) (67) *England*	*Pharmacy and sexual health clinic:* young women's experiences of accessing emergency contraception pills from pharmacies and sexual health clinics.	Qualitative; interviews	YP	21			✓	Medium	High
Turner *et al.* (2012) (68) *England*	*General practice*: exploring parents' views and experiences of primary care as a treatment setting for childhood obesity.	Qualitative; interviews	Caregivers	15		✓		Low	High
Usher-Smith *et al*. (2015) (69) *England*	*General practice and secondary care*: Explored the pathway to diagnosis of type 1 diabetes.	Quantitative; survey	Caregivers	87	✓	✓		Medium	Medium
Williams *et al.* (2014) (70) *England and Wales*	*Dental care*: the impact of a community-based dental care pathway on children's dental care entering residential or foster care.	Qualitative; interviews and routinely collected data	HCPs, social workers, CYP, and caregivers	Routinely collected data on 89 CYP<br>Dental health professionals (n=6)<br>Social workers (n=2)<br>CYP (n=3)<br>Caregivers (n=5)	✓	✓	✓	Low	Low
Williams *et al.* (2012) (71) *England*	*Not specified (preventative primary care services)*: Described African and African-Caribbean fathers' beliefs about fatherhood, health and preventive primary care services.	Qualitative; focus groups	Caregivers	9 focus groups (46 parents)	n/k	n/k	n/k	Medium	Medium
Wilson *et al.* (2021) (72) *England*	*Optometric practices*: accessibility of eye care for children with typical development and those with autism.	Quantitative; telephone survey	Optometric practices	400	✓	✓		Low	Low
Yassaee *et al.* (2017) (73) *England*	*General practice*: adolescents' experiences of their GP, whether poor reported GP experience was associated with worse physical and mental health measures and whether poor previous GP experience was linked to lower service utilisation.	Quantitative; cross-sectional survey	YP	5,335		✓		High	Low

*Reports are from the same study.

For peer review only - http://bmjopen.bmj.com/site/about/guidelines.xhtml

Facilitators and barriers in CYP access to primary care

We constructed three overarching themes on CYP and caregivers' access to primary care: deciding to access care; reaching and entering services; and communication and trust between HCPs, caregivers and CYP (see figure 2). Additional file 5 provides a table of themes by study.

Figure 2: Facilitators and barriers to CYP access to primary care

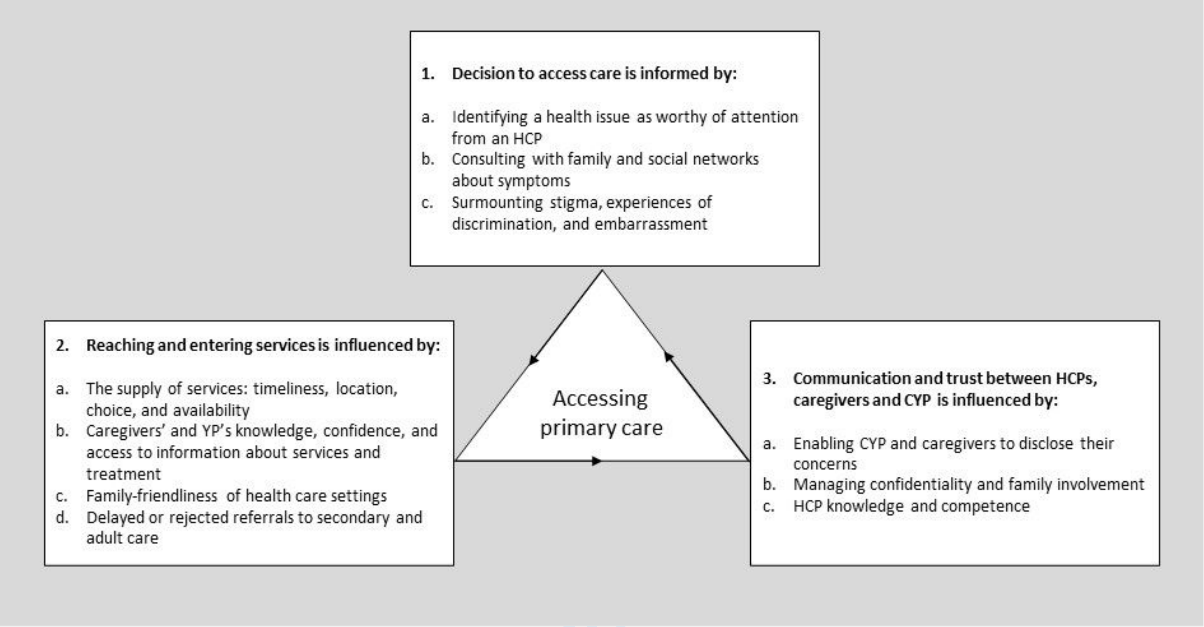

1. Deciding to access care

Multiple studies examined caregivers' and YPs' decisions to access healthcare. We constructed three sub-themes: identifying a health issue as worthy of attention from a HCP (n=9); consulting with family and social networks about symptoms (n=5); and surmounting stigma, experiences of discrimination, and embarrassment (n=17).

a. Identifying a health issue as worthy of attention from a HCP

Three studies (of medium/high-quality, in four reports) reported that a YP or caregiver would only seek help if they considered their symptoms serious, wanting to avoid burdening health system resources (47,54,55,69). As well as assessing severity, caregivers considered the familiarity of the illness, their child's level of distress, and whether symptoms were worsening and/or persisting (47,54,55,69). First-time parents were more likely to access care as childhood illnesses were unfamiliar, making it difficult for parents to judge severity (54). The basis of YP's decision-making for mental health concerns was similar to those of caregivers; they would consider seeking help if their distress was severe and enduring, and was felt to be beyond self-management (32,52).

In three studies of pre-school children, (one high-quality, two low-quality), two of which focused of CYP from deprived areas, HVs and parents reported oral health was of low priority in comparison to assessing children's physical health and developmental milestones (42,46,64).

For peer review only - http://bmjopen.bmj.com/site/about/guidelines.xhtml

b. Consulting with family and social networks about symptoms

Five studies (of medium/high-quality, in six reports) reported that parents and YP utilised their family and social networks, as well as material resources (e.g. websites, leaflets) to confirm their decision to consult (28,43,47,54,55,66). Contradictory advice or encouragement from family/friends to seek help contributed to a decision to consult (47,66).

In three high-quality studies, caregivers from South Asian, Gypsy/Travelling, Somali, and Black ethnic minority groups reported that they would defer to children's grandparents, extended family or community members for advice, and relied upon their children or local community to relay information if they could not read and write in English (28,43,54,55). Two of the studies identified that if the community were unfamiliar with the syndrome/illness studied (or it was stigmatised), families could encounter advice not to seek help, dismissive responses to diagnosis, or inappropriate efforts to treat the condition (28,43):

> *"Some of the people say, "Why are you saying something silly like this?" He's a child, he will grow out of it [autism]. A lot of children can't talk at the normal age, why don't you wait? Don't go to the doctors. He will grow out of these things."* (Caregiver, (43)).

c. Surmounting stigma, experiences of discrimination, and embarrassment

Stigma, discrimination, and embarrassment were reported as barriers to help-seeking. Four studies (of mixed quality) highlighted that parents could feel judged on their parenting, labelled as 'pushy parents', or blamed for their child's condition. This was found in studies of mothers of low socio-economic status, children with ADHD, gender diverse children, and those experiencing childhood obesity (45,62,66,68).

> *"I'm on income support, so asking me to feed her quinoas, avocados and vegetables, that's just not … I can barely get the milk for the tea. And then I have five other children, how am I going to measure the powder every meal?" (*Caregiver, (66))

Stigma and discrimination experienced by ethnic minorities and migrants were barriers identified in four studies (of mixed quality) (33,38,58,71). For example, caregivers being sent away or ignored (38) or labelled as 'aggressive' when trying to resolve misunderstandings with HCPs (71). Two studies (of medium/high-quality) reported that African/African-Caribbean fathers and migrant caregivers perceived preventative services as part of a government surveillance system, indicating distrust of services (33,71).

Stigma related to mental health felt by YP or their caregiver could be a barrier to seeking help from a GP, as reported in four studies (of mixed quality), (43,52,57,65), two of which suggested that mental health stigma was more common among ethnic minorities (43,57). Believing that they would not be taken seriously, or fears that they would not have a say in their treatment, were barriers to seeking mental health support reported by YP (34,52,65).

Embarrassment was a common barrier for YP seeking sexual health care, noted in four studies (of medium/high-quality) (27,49,51,67). YP reported being concerned about being seen by family/friends or judged by staff, feeling ashamed to be accessing emergency contraception, and embarrassed by the testing procedure itself. In one high-quality study, YP felt HCPs might make assumptions about

For peer review only - http://bmjopen.bmj.com/site/about/guidelines.xhtml

promiscuity or judge them on the basis of their sexuality, affecting their willingness to be tested in general practice (51).

2. Reaching and entering services

After caregivers or YP decided healthcare support was needed, organising an appointment and entering services was the next step to access. We identified four sub-themes among the many studies exploring this theme: the supply of services (n=22); caregivers' and YP's knowledge, confidence, and access to information about services and treatment (n=18); family-friendly healthcare settings (n=6); and delayed or rejected referrals to secondary or adult care (n=7).

a. The supply of services: timeliness, location, choice, and availability

Caregivers and YP reported that longer GP, pharmacy and sexual health clinic opening hours could facilitate more timely access to care in seven studies (of mixed quality) (27,36,51,55,60,67). Caregivers noted it could be difficult to attend (or phone for) appointments early in the morning when children were getting ready for school, or at children's bedtime, in one high-quality study (54). Caregivers were willing to seek advice and treatment from nurses, pharmacists and NHS Direct (instead of a GP) if they wanted to be seen quickly, and/or the illness was considered common and/or mild (30,47,55,60). Two studies (of medium/high-quality) found that parents sought out a private diagnosis to gain more timely access to care (for ADHD and for juvenile idiopathic arthritis) and to evade GP 'gatekeeping' (45,59).

Healthcare practices that were within walking distance of patients' homes or work, or on bus routes could facilitate access, as reported by caregivers and YP (27,30,36,49), as could co-locating health and other children's services, according to HVs and caregivers (30,42). Choice of healthcare settings and professional was salient in sexual health studies; YP appreciated options for seeking testing and advice (online, pharmacy, GP, sexual health clinic) where privacy/discretion was a key consideration (see quote below), and some YP preferred to speak to a staff member with the same gender identity (27,30,39,49,51,67).

In terms of service availability, participants from multiple studies reported long waiting times to see a GP (36,39,49,52,60,69). Reduced engagement with HVs as a result of cuts to provision was noticed by caregivers and HVs in two studies (of medium/high-quality) (30,42). HVs also noted the lack of NHS dentists in the deprived areas in which they worked (42). Three studies (of low/medium-quality) found caregivers had received conflicting information from dental practices about the age for registering children (40,53,64). One low-quality study noted that strict non-attendance and de-registration policies to manage resources in dentistry adversely affected looked after children, who often had a history of low dental attendance, poor diet and oral hygiene before care entry, and higher dental care anxiety (70):

> "*They haven't been to the dentist for a long time...then they are suddenly faced with a dental appointment, and often they are fine, and then the day before or the day of the appointment, they categorically refuse to go.*" (Caregiver, (70)).

One study (of low-quality) found optometry practices varied in whether they thought young children (under 5) should be examined by a GP or an optometrist (72).

For peer review only - http://bmjopen.bmj.com/site/about/guidelines.xhtml

b. Caregivers' and YP's knowledge, confidence, and access to information about services and treatment

Studies (of mixed quality) reported variation in caregivers' and YP's knowledge of appointment systems, though YP were more often inexperienced in accessing care (30,33,39,43,46,52). In a high-quality study on Somali migrants' access to care for CYP with autism, caregivers reported feeling overwhelmed by the complexity of the health and education system, and the lack of clarity around the purpose of appointments and professional roles (43). Caregivers of CYP with complex needs and HCPs reported that parents having the confidence to persist in asking for support for their child helped them to gain timely access to care and appropriate referrals to secondary care, as noted in multiple studies (of medium/high-quality) relating to CYP with chronic conditions, mental health problems, ADHD, and gender diversity (37,41,45,57,59,62):

> *"…if I felt a child was, not necessarily needing secondary care but the family were overly concerned and were pushing for a referral [for anxiety], I would probably [go] along with that.'* (GP, (57))

A lack of clear, visible information about what services were offered at the GP and pharmacy was reported by YP and caregivers in four studies (of a mix of quality) (39,49,51,60). Two high-quality studies identified that confusion over who was responsible for organising an interpreter was a barrier to dental and GP care (43,50). Some caregivers liked to receive practical resources and hard copies of information about child health that they could refer back to, reported in two high-quality studies focused on CYP from deprived areas (42,55). YP reported they would like demonstration videos via websites alongside instructions for self-testing in one medium-quality sexual health study (51).

c. Family-friendly healthcare settings

The healthcare setting itself could be a barrier to help-seeking. It was stressful for caregivers to wait with their child or with other children in tow, a problem particularly affecting single parents and parents without easy access to childcare (54). In some practices, the physical environment could be difficult to navigate with a buggy (64). Signalling that healthcare settings were child- and parent-friendly, for example, by putting toys in the waiting area (60,64), or being warm and approachable at the reception desk, was appreciated by caregivers and YP, particularly caregivers who were not fluent in English or YP who were struggling with their mental health (29,44,54). One medium-quality study flagged that the fathers in their study perceived child health services as designed for women, rather than men (71).

d. Delayed or rejected referrals to secondary or adult care

Delayed or rejected referrals to secondary or adult care was a barrier to CYP having their health needs met. Three studies (of medium/high-quality) about care for anxiety, ADHD, and juvenile idiopathic arthritis reported several reasons for GPs delaying referrals: a decision to 'wait and see' to see if more evidence materialised, the assumption that symptoms were the result of another non-medical cause, or were due to a pre-existing known condition (37,45,59). The feeling of being 'passed around' services was recounted by both HCP and caregivers of CYP with these conditions (45,57,59).

For peer review only - http://bmjopen.bmj.com/site/about/guidelines.xhtml

Both caregivers' and HCPs described frustration over the care of CYP's mental health and ADHD resulting from: long waiting lists for Child and Adolescent Mental Health Services (CAMHS); rejected referrals to CAMHS due to high thresholds, GPs lack of knowledge about available mental health and ADHD services, or what information is needed to obtain a successful referral; or lack of clear care pathways, reported in five studies (of mostly medium/high-quality) (44,45,48,56,57).

3. Communication and trust between HCPs, caregivers and CYP in consultations

Once a consultation with a HCP professional was arranged, accessing the help CYP needed depended upon communication and trust with HCPs. We constructed three sub-themes from multiple studies: enabling CYP and caregivers to disclose their concerns (n=22); managing confidentiality and parental involvement (n=6); and HCP knowledge and competence (n=20).

a. Enabling CYP and caregivers to disclose their concerns

A 2014 national survey of adolescents in England found that only 54% of YP who had visited the GP in the last year felt able to talk to them about personal matters (73). Numerous studies highlighted that the quality of patient encounters with HCPs impacted on their willingness to disclose information. Caregivers and YP across many studies identified the same HCP attributes that would help them to share their concerns: HCPs should be reassuring, trustworthy, and knowledgeable (27,30,34,37,51,52,60,67,70).

> '*His [the GP's] patience and lack of judgement was amazing, just to listen to my experiences of what happens for emotionally when I'm self-harming… it was incredible*.' (YP, 22 years, (52))

HCPs showing that they were listening and taking CYP's symptoms seriously was very important. Displaying scepticism or disbelief of CYP's ailments led to CYP feeling that their needs had not been met (28,34,37,39,52,54,55,66).

> *"I went back there (GP practice) quite a few times and… my GP was trying to convince me that it [Crohn's Disease] was in my head and I was just imagining it."* (YP, 24 years, (28))

Two studies of CYP and caregivers from deprived areas (one of which also focused on minority ethnic groups) highlighted that parents felt a sense of powerlessness and inferiority in the provider-patient interaction which could prevent them from sharing relevant information or leave them feeling unsupported (54,55,66).

Continuity of care was considered valuable in building a positive, trusting relationship between YP/caregivers and HCPs (30,39,41,49,54,60,61,70), and was particularly vital for CYP with mental health concerns (29,34,44,52,63). YP, caregivers, and HCP, noted that in discussions about sensitive matters, such as mental health, HCP should be careful about language used and help-seeking should be framed as a healthy and positive behaviour (37,44,49,51,61,68). Information-giving should be tailored to the individual, for example, YP attending a sexual health service might need more support on their first visit (39,67). Participants of all types in multiple studies reported that more consultation time was needed for sensitive subjects, notably mental health, or when support needs were high (34,39,52,61,63,66,67,70).

For peer review only - http://bmjopen.bmj.com/site/about/guidelines.xhtml

b. Confidentiality and family involvement

YP, particularly those with mental health problems, expressed concern that information about them would be shared with family or other professionals without their consent, as reported in four mixed-quality studies (34,39,52,63). Parents could be a facilitator or a barrier to mental health consultations with YP: parents could facilitate access by encouraging them to attend and supporting their account; or parents could inhibit the YP from sharing information if the YP did not want to upset them, if they wanted something different from their parent, or their parental relationship was part of the problem (44,48,63).

c. HCP knowledge and competence

Studies highlighted multiple areas where HCPs lacked sufficient expertise to manage care (see table 2). GP management of CYPs' mental health was the knowledge and competency gap most often reported by YP, caregivers and HCPs. It included: presentation of different conditions; how to enable CYP to share their concerns; knowledge of available treatment options and CAMHS services; and managing potential risks of approaching sensitive topics in front of family members (see table 2). If there was a delay or unsuccessful referral in accessing secondary or adult care (see "Delayed or reject referrals"), then the GP remained the (non-expert) provider of care in the interim (29,45,48,57,63). Managing physical changes from puberty while waiting for specialist care for gender diversity was a new area where expertise was required (62).

YP and caregiver trust in HCPs' expertise could diminish when repeated consultations resulted in little improvement or misdiagnosis, and was a barrier to seeking further help from primary care, as reported in multiple studies (of predominantly medium/high-quality), three focusing on CYP with chronic health conditions and three on ethnic minority groups (28,41,47,54,59,71). Thus, experiences of communication and trust affected the decision to access care in the future.

Barriers affecting equitable access to care

Specific barriers affecting access to care across themes were mapped for several sub-populations with known higher health needs (see table 3). Multiple trust-related barriers were reported by ethnic minority caregivers and YP resulting from negative past experiences with unfriendly staff, or unsatisfactory support or diagnosis, combined with a need for more accessible and culturally appropriate health information. Many barriers to seeking mental health support were identified by YP, caregivers and HCP, including: a lack of patient and HCP awareness of treatment options, and organisational processes which diminished relationship-building between YP and HCPs (e.g. short appointments, less continuity of care). Commons barriers reported across sub-populations were caregivers needing to have the knowledge or confidence to ask for the help they needed or to challenge a HCP whose advice they disagreed with, gaps in HCP knowledge and in communication between primary and secondary care.

For peer review only - http://bmjopen.bmj.com/site/about/guidelines.xhtml

Table 2: Reported variability/gaps in HCP knowledge

Variability/gaps in HCP knowledge in treating CYP	Reported by	References	Quality rating of references
**General practitioners**			
Mental health: presentation of different conditions; enabling CYP to share their concerns; knowledge of available treatment options and CAMHS services; managing potential risks of approaching sensitive topics in front of family members.	CYP<br>HCPs<br>Caregivers	(29,34,44,48,57,63)	3 High,<br>1 Medium,<br>2 Low
Allergy management and referrals to secondary care.	Caregivers	(41)	1 Medium
The needs of primary-aged gender diverse children and support services available.	Caregivers	(62)	1 Medium
Identifying and managing juvenile arthritis.	HCP	(59)	1 High
ADHD aetiology, identification, diagnosis, referral processes, services available.	HCP<br>CYP<br>Caregivers	(45)	1 Medium
The experiences and needs of families from ethnic minority groups.	Caregivers<br>CYP	(28,71)	1 High,<br>1 Medium
How to sensitively and effectively address childhood obesity, particularly when caregivers have struggled with their own weight.	Caregivers	(68)	1 Low
**Dentistry**			
Managing children with learning difficulties.	HCP	(35)	1 Low
**Health visiting teams**			
Oral health promotion, culturally specific oral health guidance, knowledge of local dentistry services.	HCP	(42,50)	2 High
Culturally specific advice concerning feeding practices.	Caregivers<br>HCP	(58)	1 High
How to address childhood obesity.	HCPs	(61)	1 Low

For peer review only

Table 3: Barriers to accessing care for sub-populations of CYP

Sub-population (no. of studies)	Reported barriers to access
CYP with mental health problems (n=11)	• *Decision to access*: Stigma related to mental health. CYP believing they would not be taken seriously or would not have a say in their treatment. CYP believing they could self-manage.<br>• *Reaching and entering services:* Caregivers feeling hesitant to persist in asking for support for their child. Unfriendly reception staff. Delayed or rejected referrals to CAMHS or AMH.<br>• *Communication and trust*: A lack of continuity of care and insufficient time in consultations. YP concerns about confidentiality. GPs lacking knowledge in how to manage CYP mental health.
CYP from deprived areas (n=8)	• *Decision to access*: CYP oral health was a lower priority for some caregivers than children's physical health and developmental milestones. Caregivers feeling judged on their parenting or blamed for their child's condition.<br>• *Reaching and entering services*: Caregivers lacking practical resources and non-digital information.<br>• *Communication and trust*: Caregivers feeling a sense of powerless and inferiority in the provider-patient interaction.
Looked after children (n=2)	• *Reaching and entering services*: Strict non-attendance and de-registration policies.<br>• *Communication and trust*: A lack of continuity of care and insufficient time in consultations.
Ethnic minority CYP (n=7)	• *Decision to access*: A lack of familiarity within the community of the syndrome/illness and stigma related to mental health. Perception of surveillance by healthcare systems. Experiences of stigma and discrimination. Lack of health information in other languages.<br>• *Reaching and entering services*: Unfriendly reception staff. Lack of knowledge of the healthcare (and education) system.<br>• *Communication and trust:* Repeated consultations resulting in little improvement or misdiagnosis. Lack of GP knowledge about the experiences and needs of ethnic minority groups. Health visiting teams lacking knowledge of culturally specific oral health guidance and feeding practices.
CYP with SEND (n=5)	• *Decision to access*: Caregivers feeling judged on their parenting or blamed for their child's condition.<br>• *Reaching and entering services*: Lack of knowledge of the healthcare (and education) system. Delayed or rejected referrals to secondary or adult care. Caregivers feeling hesitant to persist in asking for support for their child.<br>• *Communication and trust*: Dentists lacking knowledge in caring for CYP with learning difficulties.
CYP with chronic health problems (n=4)	• *Reaching and entering services*: Delayed or rejected referrals to secondary care. Caregivers feeling hesitant to persist in asking for support for their child.<br>• *Communication and trust:* Repeated consultations resulting in little improvement or misdiagnosis. Lack of GP knowledge about some childhood chronic health problems.

For peer review only

Discussion

Summary

The review identified high-quality evidence, from multiple studies and informants, that CYP access to primary care was affected by caregivers and YP knowing whether symptoms/conditions could be managed at home or healthcare expertise was needed. Levels of patients' health and language literacy, access to legitimate health advice via social networks or culturally-appropriate resources, and patients' expectations affect equitable and appropriate use of primary care (11,74,75). This suggests multi-lingual public health information about childhood symptoms/conditions, when and how to seek help should be available online and in public spaces, and professionals that bridge community and primary care services (for example, third sector health workers, health visitors, school nurses, family hub workers) should support caregivers/YP *into* primary care when they identify healthcare needs and there are known language, cultural or trust-related barriers to accessing services (76,77).

Many high-quality studies suggested that CYP access to services could be improved by making them easier to reach and enter, for example, by extending opening hours and co-located services. Signals that health care settings were family-friendly, such as having children's books in reception and welcoming reception staff were quick-wins. Flexibility, for example, having the option to call, drop-in, *or* use an online system to make an appointment, could facilitate access for caregivers with different needs and preferences in time, communication and support (78). Wealthier caregivers were able to circumvent blocks to timely secondary care by accessing private health care, but this was not possible for all caregivers, suggesting that waiting lists are likely to disadvantage poorer CYP. This is particularly concerning in dentistry where 27,000 children were on NHS waiting lists for specialist dental care, assessment or procedures in January 2023 (79). Combined with general practice workforce shortages (14), increased CYP morbidities (15), and lower caregiver self-efficacy, health and language literacy in deprived areas, the importance of proactive efforts to address inequalities is evident (78).

Although improving CYP access to mental health care is a high policy priority (8,26), there was strong evidence that YP were reluctant to consult with GPs about mental health concerns without a pre-existing relationship with them. Prioritising continuity for YP when GPs are increasingly working part-time and locuming needs consideration (80,81). Caregivers, YP, and HCPs also reported gaps in GPs knowledge/competence in managing CYP mental health, and long-wait times and rejected referrals to secondary care, indicating a need to increase medical training in child and adolescent mental health (82,83). Although school-based interventions may alleviate concerns for some children, evidence from large-scale mixed-method evaluations suggests that CYP with moderately high emotional needs and those with additional needs (for example, neurodiversity, SEND or difficult family circumstances) may fall through the gaps (84,85). There are examples of integrated approaches for children with chronic health conditions whereby GPs are supported by specialists which could bridge this gap including in mental health (86,87). Social prescribing may also be able to support CYP waiting for CAMHS, though the evidence for this is not yet known (88).

For peer review only - http://bmjopen.bmj.com/site/about/guidelines.xhtml

Strengths and limitations

Our review was rigorously conducted and included quality appraisal. Mapping patterns of facilitators/barriers across different sub-populations with higher health needs revealed that access was affected by caregivers' needing to be able to confidently advocate for their child's needs. It also highlighted the multi-layered barriers that exist for some groups, including ethnic minority CYP, and the lack of current evidence on access for looked after children. Regarding limitations, we only double-screened 20% of title/abstracts and we may have missed reports due to the array of terms for primary care. We could not screen studies in systematic reviews or search for grey literature due to time and resources constraints, and we may have missed relevant reports, particularly for marginalised groups (e.g. LGBTQ+ YP). Supply barriers to access, e.g. recruiting and retaining GPs, were not identified using our search terms likely because they are relevant to access for all patients.

Conclusions

The review evidence suggests that four policy priorities to improve equitable CYP access to primary care: 1) encouraging CYP/caregivers into healthcare settings through general practices developing and maintaining links with community health workers/services, 2) improving CYP/caregivers' understanding of common childhood conditions by providing public health information on common childhood conditions and illnesses in local languages, 3) developing integrated approaches bringing specialist expertise into primary care, and 4) addressing paediatric training gaps for medical students, particularly in child and adolescent mental health.

References

1. Levesque JF, Harris MF, Russell G. Patient-centred access to health care: conceptualising access at the interface of health systems and populations. Int J Equity Health. 2013;12(1):18.

2. Gulliford M, Figueroa-Munoz J, Morgan M, Hughes D, Gibson B, Beech R, et al. What does 'access to health care' mean? J Health Serv Res Policy. 2002 Jul 1;7(3):186–8.

3. Harnden A, Mayon-White R, Mant D, Kelly D, Pearson G. Child deaths: confidential enquiry into the role and quality of UK primary care. Br J Gen Pract. 2009 Nov 1;59(568):819–24.

4. Royal College of Nursing. Safeguarding children and young people: roles and competencies for healthcare staff: intercollegiate document [Internet]. London: Royal College of Nursing; 2019 Jan [cited 2023 Jan 13]. Available from: https://www.rcn.org.uk/professional-development/publications/pub-007366

5. Cecil E, Bottle A, Cowling T, Majeed A, Wolfe I, Saxena S. Primary Care Access, Emergency Department Visits, and Unplanned Short Hospitalizations in the UK. Pediatrics. 2016 Feb;137(2):e20151492–e20151492.

6. Gill PJ, Goldacre MJ, Mant D, Heneghan C, Thomson A, Seagroatt V, et al. Increase in emergency admissions to hospital for children aged under 15 in England, 1999-2010: national database analysis. Arch Dis Child. 2013 May 1;98(5):328–34.

7. Ruzangi J, Blair M, Cecil E, Greenfield G, Bottle A, Hargreaves D. Trends in healthcare use in children aged less than 15 years: A population-based cohort study in England from 2007 to 2017. BMJ Open. 2020;10(5):e033761–e033761.

For peer review only - http://bmjopen.bmj.com/site/about/guidelines.xhtml

8. NHS. The NHS long term plan [Internet]. 2019 [cited 2021 Oct 1]. Available from: https://www.longtermplan.nhs.uk/

9. Hargreaves DS, Elliott MN, Viner RM, Richmond TK, Schuster MA. Unmet Health Care Need in US Adolescents and Adult Health Outcomes. Pediatrics. 2015 Sep 1;136(3):513–20.

10. Ghafari M, Nadi T, Bahadivand-Chegini S, Doosti-Irani A. Global prevalence of unmet need for mental health care among adolescents: A systematic review and meta-analysis. Arch Psychiatr Nurs. 2022 Feb;36:1–6.

11. Coughlan C, Ruzangi J, Neale F, Nezafat Maldonado B, Blair M, Bottle A, et al. Social and ethnic group differences in healthcare use by children aged 0-14 years: A population-based cohort study in England from 2007 to 2017. Arch Dis Child. 2022;107(1):32–9.

12. Perry M, McGowan A, Roberts R, Cottrell S. Timeliness and equity of infant pertussis vaccination in wales: Analysis of the three dose primary course. Vaccine. 2020;38(6):1402–7.

13. Bishop C, Small N, Parslow R. Healthcare use for children with complex needs: Using routine health data linked to a multiethnic, ongoing birth cohort. BMJ Open. 2018;8(3):e018419–e018419.

14. Nussbaum C, Massou E, Fisher R, Morciano M, Harmer R, Ford J. Inequalities in the distribution of the general practice workforce in England: a practice-level longitudinal analysis. BJGP Open. 2021 Oct;5(5):BJGPO.2021.0066.

15. Chung SC, Mueller S, Green K, Chang WH, Hargrave D, Lai AG. Multimorbidity patterns and risk of hospitalisation in children: A population cohort study of 3.6 million children in England, with illustrative examples from childhood cancer survivors. Lancet Reg Health - Eur. 2022 Sep;20:100433.

16. Coughlan B, Duschinsky R, O'Connor M, Woolgar M. Identifying and managing care for children with autism spectrum disorders in general practice: A systematic review and narrative synthesis. Health Soc Care Community. 2020 Nov;28(6):1928–41.

17. Chirewa B, Wakhisi A. Emergency hormonal contraceptive service provision via community pharmacies in the UK: a systematic review of pharmacists' and young women's views, perspectives and experiences. Perspect Public Health. 2020 Mar;140(2):108–16.

18. Ahmadyar M, Rai T, Daly B, Wong G. Improving access of young adults with experience of homelessness to primary care dental services in the UK: A realist synthesis. Community Dent Oral Epidemiol. 2022 Oct;50(5):437–44.

19. McDonagh LK, Saunders JM, Cassell J, Curtis T, Bastaki H, Hartney T, et al. Application of the COM-B model to barriers and facilitators to chlamydia testing in general practice for young people and primary care practitioners: a systematic review. Implement Sci. 2018 Dec;13(1):130.

20. Tricco AC, Lillie E, Zarin W, O'Brien KK, Colquhoun H, Levac D, et al. PRISMA extension for scoping reviews (PRISMA-ScR): Checklist and explanation. Ann Intern Med. 2018;169(7):467–73.

21. Pace R, Pluye P, Bartlett G, Macaulay AC, Salsberg J, Jagosh J, et al. Testing the reliability and efficiency of the pilot Mixed Methods Appraisal Tool (MMAT) for systematic mixed studies review. Int J Nurs Stud. 2012 Jan;49(1):47–53.

For peer review only - http://bmjopen.bmj.com/site/about/guidelines.xhtml

22. Souto RQ, Khanassov V, Hong QN, Bush PL, Vedel I, Pluye P. Systematic mixed studies reviews: Updating results on the reliability and efficiency of the mixed methods appraisal tool. Int J Nurs Stud. 2015 Jan;52(1):500–1.

23. Gough D. Weight of Evidence: a framework for the appraisal of the quality and relevance of evidence. Res Pap Educ. 2007 Jun;22(2):213–28.

24. Gale NK, Heath G, Cameron E, Rashid S, Redwood S. Using the framework method for the analysis of qualitative data in multi-disciplinary health research. BMC Med Res Methodol. 2013 Dec;13(1):117.

25. Ford JA, Wong G, Jones AP, Steel N. Access to primary care for socioeconomically disadvantaged older people in rural areas: a realist review. BMJ Open. 2016 May 1;6(5):e010652.

26. NHS England. Core20PLUS5 – An approach to reducing health inequalities for children and young people [Internet]. 2022 [cited 2023 Aug 16]. Available from: https://www.england.nhs.uk/about/equality/equality-hub/national-healthcare-inequalities-improvement-programme/core20plus5/core20plus5-cyp/

27. Ahmaro L, Lindsey L, Forrest S, Whittlesea C. Young people's perceptions of accessing a community pharmacy for a chlamydia testing kit: a qualitative study based in North East England. BMJ Open. 2021 Sep;11(9):e052228.

28. Alexakis C, Nash A, Lloyd M, Brooks F, Lindsay JO, Poullis A. Inflammatory bowel disease in young patients: challenges faced by black and minority ethnic communities in the UK. Health Soc Care Community. 2015 Nov;23(6):665–72.

29. Appleton R, Loew J, Mughal F. Young people who have fallen through the mental health transition gap: a qualitative study on primary care support. Br J Gen Pract. 2022 Jun;72(719):e413–20.

30. Bosley H, Appleton JV, Henshall C, Jackson D. The influence of perceived accessibility and expertise of healthcare professionals, and service austerity, on mothers' decision-making. Health Soc Care Community. 2021 Mar;29(2):526–34.

31. Brigham L, Maxwell C, Smith A. Leading in practice: a case study of how health visitors share and develop good practice. Community Pract J Community Pract Health Visit Assoc. 2012 May;85(5):24–8.

32. Coleman-Fountain E, Buckley C, Beresford B. Improving mental health in autistic young adults: a qualitative study exploring help-seeking barriers in UK primary care. Br J Gen Pract. 2020 May;70(694):e356–63.

33. Condon L, McClean S, McRae L. 'Differences between the earth and the sky': migrant parents' experiences of child health services for pre-school children in the UK. Prim Health Care Res Dev. 2020;21:e29.

34. Corry DAS, Leavey G. Adolescent trust and primary care: Help-seeking for emotional and psychological difficulties. J Adolesc. 2017 Jan;54(1):1–8.

35. Coyle CF, Humphris GM, Freeman R. Dentists' training and willingness to treat adolescents with learning disabilities: the mediating role of social and clinical factors. Community Dent Health. 2013 Dec;30(4):263–8.

For peer review only - http://bmjopen.bmj.com/site/about/guidelines.xhtml

36. Crocker JC, Evans MR, Powell CVE, Hood K, Butler CC. Why some children hospitalized for pneumonia do not consult with a general practitioner before the day of hospitalization. Eur J Gen Pract. 2013 Dec;19(4):213–20.

37. Crouch L, Reardon T, Farrington A, Glover F, Creswell C. “Just keep pushing”: Parents’ experiences of accessing child and adolescent mental health services for child anxiety problems. Child Care Health Dev. 2019 May 27;cch.12672.

38. Dando CJ, Brierley R, Saunders K, Mackenzie JM. Health inequalities and health equity challenges for victims of modern slavery. J Public Health. 2019 Dec 20;41(4):681–8.

39. Davey A, Asprey A, Carter M, Campbell JL. Trust, negotiation, and communication: young adults’ experiences of primary care services. BMC Fam Pract. 2013 Dec;14(1):202.

40. Dickson CM. Every child has the right to smile!--A qualitative study exploring barriers to dental registration in a SureStart area in Northern Ireland. Community Pract J Community Pract Health Visit Assoc. 2015 Aug;88(8):36–9, 41.

41. Diwakar L, Cummins C, Hackett S, Rees M, Charles L, Kerrigan C, et al. Parent experiences with paediatric allergy pathways in the West Midlands: A qualitative study. Clin Exp Allergy. 2019 Mar;49(3):357–65.

42. Eskytė I, Gray-Burrows KA, Owen J, Sykes-Muskett B, Pavitt SH, West R, et al. Organizational Barriers to Oral Health Conversations Between Health Visitors and Parents of Children Aged 9–12 Months Old. Front Public Health. 2021 Feb 23;9:578168.

43. Fox F, Aabe N, Turner K, Redwood S, Rai D. “It was like walking without knowing where I was going”: A Qualitative Study of Autism in a UK Somali Migrant Community. J Autism Dev Disord. 2017 Feb;47(2):305–15.

44. Fox F, Stallard P, Cooney G. GPs role identifying young people who self-harm: a mixed methods study. Fam Pract. 2015 May 8;cmv031.

45. French B, Perez Vallejos E, Sayal K, Daley D. Awareness of ADHD in primary care: stakeholder perspectives. BMC Fam Pract. 2020 Dec;21(1):45.

46. Henderson E, Rubin G. A model of roles and responsibilities in oral health promotion based on perspectives of a community-based initiative for pre-school children in the UK. Br Dent J. 2014 Mar;216(5):E11–E11.

47. Ingram J, Cabral C, Hay AD, Lucas PJ, Horwood J. Parents’ information needs, self-efficacy and influences on consulting for childhood respiratory tract infections: a qualitative study. BMC Fam Pract. 2013 Dec;14(1):106.

48. Jobanputra S, Singh S. What are general practitioners’ views on the management of adolescents with mental health disorders? A qualitative study. Educ Prim Care. 2020 Sep 2;31(5):323–4.

49. Jones LF, Ricketts E, Town K, Rugman C, Lecky D, Folkard K, et al. Chlamydia and HIV testing, contraception advice, and free condoms offered in general practice: a qualitative interview study of young adults’ perceptions of this initiative. Br J Gen Pract. 2017 Jul;67(660):e490–500.

For peer review only

50. Lewney J, Holmes RD, Rankin J, Exley C. Health visitors' views on promoting oral health and supporting clients with dental health problems: a qualitative study. J Public Health. 2019 Mar 1;41(1):e103–8.

51. McDonagh LK, Harwood H, Saunders JM, Cassell JA, Rait G. How to increase chlamydia testing in primary care: a qualitative exploration with young people and application of a meta-theoretical model. Sex Transm Infect. 2020 Dec;96(8):571–81.

52. Mughal F, Dikomitis L, Babatunde OO, Chew-Graham CA. Experiences of general practice care for self-harm: a qualitative study of young people's perspectives. Br J Gen Pract. 2021 Oct 1;71(711):e744–52.

53. Muirhead V, Subramanian SK, Wright D, Wong FSL. How do foster carers manage the oral health of children in foster care? A qualitative study. Community Dent Oral Epidemiol. 2017 Dec;45(6):529–37.

54. Neill SJ, Jones CH, Lakhanpaul M, Roland DT, Thompson MJ. Parents' help-seeking behaviours during acute childhood illness at home: A contribution to explanatory theory. J Child Health Care. 2016 Mar;20(1):77–86.

55. Neill SJ, Jones CHD, Lakhanpaul M, Roland DT, Thompson MJ, the ASK SNIFF research team. Parent's information seeking in acute childhood illness: what helps and what hinders decision making? Health Expect. 2015 Dec;18(6):3044–56.

56. O'Brien D, Harvey K, Creswell C. Barriers to and facilitators of the identification, management and referral of childhood anxiety disorders in primary care: a survey of general practitioners in England. BMJ Open. 2019 Apr;9(4):e023876.

57. O'Brien D, Harvey K, Young B, Reardon T, Creswell C. GPs' experiences of children with anxiety disorders in primary care: a qualitative study. Br J Gen Pract. 2017 Dec;67(665):e888–98.

58. Ochieng BMN. Healthy weight maintenance strategy in early childhood: The views of black African migrant parents and health visitors. Health Soc Care Community. 2020 Sep;28(5):1551–9.

59. Rapley T, May C, Smith N, Foster HE. 'Snakes & Ladders': factors influencing access to appropriate care for children and young people with suspected juvenile idiopathic arthritis – a qualitative study. Pediatr Rheumatol. 2021 Dec;19(1):43.

60. Rashed AN, Mohamud N, Lam A, Hamadallah H, Terry D, Tomlin S. Community pharmacy role in children's health in England: Experiences and opinions of parents and young people. Health Soc Care Community. 2022 Nov;30(6):2362–71.

61. Redsell SA, Swift JA, Nathan D, Siriwardena AN, Atkinson P, Glazebrook C. UK health visitors' role in identifying and intervening with infants at risk of developing obesity: Health visitors' and infant obesity risk. Matern Child Nutr. 2013 Jul;9(3):396–408.

62. Rickett B, Johnson K, Ingle H, Reynolds M. Support for parents/carers of primary school aged gender diverse children in England, UK: a mixed-method analysis of experiences with health services. Health Sociol Rev. 2021 Jan 2;30(1):9–24.

For peer review only - http://bmjopen.bmj.com/site/about/guidelines.xhtml

63. Roberts J, Crosland A, Fulton J. Patterns of engagement between GPs and adolescents presenting with psychological difficulties: a qualitative study. Br J Gen Pract. 2014 May;64(622):e246–54.

64. Roberts K, Condon L. How do parents look after children's teeth? A qualitative study of attitudes to oral health in the early years. Community Pract J Community Pract Health Visit Assoc. 2014 Apr;87(4):32–5.

65. Salaheddin K, Mason B. Identifying barriers to mental health help-seeking among young adults in the UK: a cross-sectional survey. Br J Gen Pract. 2016 Oct;66(651):e686–92.

66. Satherley RM, Wolfe I, Lingam R. Experiences of healthcare for mothers of children with ongoing illness, living in deprived neighbourhoods health and place. Health Place. 2021 Sep;71:102661.

67. Turnbull G, Scott RH, Mann S, Wellings K. Accessing emergency contraception pills from pharmacies: the experience of young women in London. BMJ Sex Reprod Health. 2021 Jan;47(1):27–31.

68. Turner KM, Salisbury C, Shield JPH. Parents' views and experiences of childhood obesity management in primary care: a qualitative study. Fam Pract. 2012 Aug 1;29(4):476–81.

69. Usher-Smith JA, Thompson MJ, Zhu H, Sharp SJ, Walter FM. The pathway to diagnosis of type 1 diabetes in children: a questionnaire study. BMJ Open. 2015 Mar 17;5(3):e006470–e006470.

70. Williams A, Mackintosh J, Bateman B, Holland S, Rushworth A, Brooks A, et al. The development of a designated dental pathway for looked after children. Br Dent J. 2014 Feb;216(3):E6–E6.

71. Williams R, Hewison A, Stewart M, Liles C, Wildman S. 'We are doing our best': African and African-Caribbean fatherhood, health and preventive primary care services, in England: African and African-Caribbean fatherhood and preventive primary care services. Health Soc Care Community. 2012 Mar;20(2):216–23.

72. Wilson S, Ctori I, Suttle C, Conway M, Shah R. How accessible is primary eye care for children in England? Ophthalmic Physiol Opt. 2021 Sep;41(5):1021–33.

73. Yassaee AA, Hargreaves DS, Chester K, Lamb S, Hagell A, Brooks FM. Experience of Primary Care Services Among Early Adolescents in England and Association With Health Outcomes. J Adolesc Health. 2017 Apr 1;60(4):388–94.

74. Viner RM, Blackburn F, White F, Mannie R, Parr T, Nelson S, et al. The impact of out-of-hospital models of care on paediatric emergency department presentations. Arch Dis Child. 2018 Feb;103(2):128–36.

75. Dale J, Potter R, Owen K, Parsons N, Realpe A, Leach J. Retaining the general practitioner workforce in England: what matters to GPs? A cross-sectional study. BMC Fam Pract. 2015 Dec;16(1):140.

76. Vanden Bossche D, Willems S, Decat P. Understanding Trustful Relationships between Community Health Workers and Vulnerable Citizens during the COVID-19 Pandemic: A Realist Evaluation. Int J Environ Res Public Health. 2022 Feb 22;19(5):2496.

For peer review only - http://bmjopen.bmj.com/site/about/guidelines.xhtml

77. Canvin K, Jones C, Marttila A, Burstrom B, Whitehead M. Can I risk using public services? Perceived consequences of seeking help and health care among households living in poverty: qualitative study. J Epidemiol Community Health. 2007 Nov 1;61(11):984–9.

78. Gkiouleka A, Wong G, Sowden S, Bambra C, Siersbaek R, Manji S, et al. Reducing health inequalities through general practice. Lancet Public Health. 2023 Jun;8(6):e463–72.

79. Liberal Democrats. 27,000 children on waiting lists for specialised dental care [Internet]. 2023 [cited 2023 Sep 5]. Available from: https://www.libdems.org.uk/press/release/27000-children-on-waiting-lists-for-specialised-dental-care

80. Jefferson L, Holmes M. GP workforce crisis: what can we do now? Br J Gen Pract. 2022 May;72(718):206–7.

81. Simon C, Forde E, Fraser A, Wedderburn C, Aylwin S. What is the root cause of the GP workforce crisis? Br J Gen Pract. 2018 Dec;68(677):589–90.

82. England E, Nash V, Hawthorne K. GP training in mental health needs urgent reform. BMJ. 2017 Mar 16;j1311.

83. Salmon G, Tombs M. Teaching medical students child and adolescent psychiatry (CAP). J Ment Health Train Educ Pract. 2019 Aug 29;14(5):348–60.

84. Ellins J, Hocking L, Al-Haboubi M, Newbould J, Fenton SJ, Daniel K, et al. Early evaluation of the Children and Young People's Mental Health Trailblazer programme: a rapid mixed-methods study. Health Soc Care Deliv Res. 2023 Jun;1–137.

85. Wolpert M, Humphrey N, Belsky J, Deighton J. Embedding mental health support in schools: learning from the Targeted Mental Health in Schools (TaMHS) national evaluation. Emot Behav Difficulties. 2013 Sep;18(3):270–83.

86. Woodman J, Lewis H, Cheung R, Gilbert R, Wijlaars LP. Integrating primary and secondary care for children and young people: sharing practice. Arch Dis Child. 2016 Sep;101(9):792–7.

87. Patel S, Hodgkinson T, Fowler R, Pryde K, Ward R. Integrating acute services for children and young people across primary and secondary care. Br J Gen Pract. 2020 Apr;70(693):158–9.

88. Fancourt D, Burton A, Bu F, Deighton J, Turner R, Wright J, et al. Wellbeing while waiting evaluating social prescribing in CAMHS: study protocol for a hybrid type II implementation-effectiveness study. BMC Psychiatry. 2023 May 10;23(1):328.

Contributors

LH, CP, JW and SM contributed to the study's conception, LH conducted the searches, and LH and EA completed the screening, with support from German Alarcon Garavito, Macarena Chepo, Sophie Moniz, Federico Redin, and Cecilia Vindrola-Padros from the RREAL Lab. LH, EA and CP conducted the data extraction and LH and EA carried out the data synthesis. LH lead and EA contributed to drafting the manuscripts and all authors provided critical revisions and editing. All authors reviewed the manuscript.

For peer review only - http://bmjopen.bmj.com/site/about/guidelines.xhtml

Funding

This study was funded by the National Institute for Health and Care Research (NIHR) through the Children and Families Policy Research Unit (PR-PRU-1217-21301). The views expressed are those of the authors and not necessarily those of the NIHR or the Department of Health and Social Care.

Competing interests

The authors declare no conflicts of interest.

Patient and public involvement

Patients and/or the public were not involved in the design, conduct or reporting of this review.

For peer review only

For peer review only - http://bmjopen.bmj.com/site/about/guidelines.xhtml

Supplementary table: Health topic and CYP population studied

Health topic (e.g., dental, sexual)	Specific population	Primary healthcare setting	Citations
**Non-specific health condition (n=13)**	• Non-specific (n=5)<br>• African and African-Caribbean fathers (n=1)<br>• CYP from different socio-economic and ethnic groups (n=1)<br>• CYP living in deprived areas (n=1)<br>• CYP of Albanian survivors of modern slavery and sexual exploitation (n=1)<br>• CYP migrants from Romania, Poland, Pakistan, or Somalia (n=1)<br>• CYP with ADHD (n=1)<br>• CYP with autism from an ethnic minority/migrant community (n=1)<br>• South Asian and Gypsy/Travelling communities (n=1)	• General practice (n=4)<br>• General practice and health visiting (n=3)<br>• Primary care (all except dentist, n=2)<br>• General practice and walk-in centres (n=1)<br>• Health visiting (n=1)<br>• General practice and pharmacy (n=1)<br>• Not specified (preventative primary care services, n=1)	(30,31,33,38,39,43,45,54,55,60,66,71,73)
**Mental health (n=11)**	• Non-specific (n=9)<br>• CYP living in deprived areas (n=1)<br>• CYP with autism (n=1)	• General practice (n=11)	(29,32,34,37,44,48,52,56,57,63,65)
**Gender diversity (n=1)**	• Non-specific (n=1)	• General practice (n=1)	(62)
**Chronic conditions (n=4) (allergies, n=1; inflammatory bowel disease, n=1; juvenile idiopathic arthritis, n=1; type 1 diabetes, n=1)**	• Non-specific (n=3)<br>• Black and minority ethnic CYP (n=1)	• General practice (n=3)<br>• Primary care (all except optometry) (n=1)	(28,41,59,69)
**Physical health (n=4) (obesity, n=3; pneumonia or empyema, n=1; respiratory tract infections, n=1)**	• Non-specific (n=3)<br>• Black African CYP (n=1)	• General practice (n=2)<br>• Health visiting (n=2)	(36,47,58,61,68)

For peer review only

**Oral health (n=8)**	• Non-specific (n=2)<br>• CYP in care (n=2)<br>• CYP living in deprived areas (n=3)<br>• CYP with learning disabilities (n=1)	• Dental care (n=6)<br>• Health visiting (n=2)	(35,40,42,46,50,53,64,70)
**Sexual health (n=4)**	• Non-specific (n=4)	• General practice (n=2)<br>• Pharmacy (n=1)<br>• Pharmacy and sexual health clinic (n=1)	(27,49,51,67)
**Optometry (n=1)**	• Younger CYP and CYP with autism (n=1)	• Optometric practices (n=1)	(72)

For peer review only

Preferred Reporting Items for Systematic reviews and Meta-Analyses extension for Scoping Reviews (PRISMA-ScR) Checklist

SECTION	ITEM	PRISMA-ScR CHECKLIST ITEM	REPORTED ON PAGE #
**TITLE**			
Title	1	Identify the report as a scoping review.	
**ABSTRACT**			
Structured summary	2	Provide a structured summary that includes (as applicable): background, objectives, eligibility criteria, sources of evidence, charting methods, results, and conclusions that relate to the review questions and objectives.	
**INTRODUCTION**			
Rationale	3	Describe the rationale for the review in the context of what is already known. Explain why the review questions/objectives lend themselves to a scoping review approach.	
Objectives	4	Provide an explicit statement of the questions and objectives being addressed with reference to their key elements (e.g., population or participants, concepts, and context) or other relevant key elements used to conceptualize the review questions and/or objectives.	
**METHODS**			
Protocol and registration	5	Indicate whether a review protocol exists; state if and where it can be accessed (e.g., a Web address); and if available, provide registration information, including the registration number.	
Eligibility criteria	6	Specify characteristics of the sources of evidence used as eligibility criteria (e.g., years considered, language, and publication status), and provide a rationale.	
Information sources*	7	Describe all information sources in the search (e.g., databases with dates of coverage and contact with authors to identify additional sources), as well as the date the most recent search was executed.	
Search	8	Present the full electronic search strategy for at least 1 database, including any limits used, such that it could be repeated.	
Selection of sources of evidence†	9	State the process for selecting sources of evidence (i.e., screening and eligibility) included in the scoping review.	
Data charting process‡	10	Describe the methods of charting data from the included sources of evidence (e.g., calibrated forms or forms that have been tested by the team before their use, and whether data charting was done independently or in duplicate) and any processes for obtaining and confirming data from investigators.	
Data items	11	List and define all variables for which data were sought and any assumptions and simplifications made.	
Critical appraisal of individual sources of evidence§	12	If done, provide a rationale for conducting a critical appraisal of included sources of evidence; describe the methods used and how this information was used in any data synthesis (if appropriate).	
Synthesis of results	13	Describe the methods of handling and summarizing the data that were charted.	

St. Michael's
Inspired Care. Inspiring Science.

For peer review only - http://bmjopen.bmj.com/site/about/guidelines.xhtml

SECTION	ITEM	PRISMA-ScR CHECKLIST ITEM	REPORTED ON PAGE #
**RESULTS**			
Selection of sources of evidence	14	Give numbers of sources of evidence screened, assessed for eligibility, and included in the review, with reasons for exclusions at each stage, ideally using a flow diagram.	
Characteristics of sources of evidence	15	For each source of evidence, present characteristics for which data were charted and provide the citations.	
Critical appraisal within sources of evidence	16	If done, present data on critical appraisal of included sources of evidence (see item 12).	
Results of individual sources of evidence	17	For each included source of evidence, present the relevant data that were charted that relate to the review questions and objectives.	
Synthesis of results	18	Summarize and/or present the charting results as they relate to the review questions and objectives.	
**DISCUSSION**			
Summary of evidence	19	Summarize the main results (including an overview of concepts, themes, and types of evidence available), link to the review questions and objectives, and consider the relevance to key groups.	
Limitations	20	Discuss the limitations of the scoping review process.	
Conclusions	21	Provide a general interpretation of the results with respect to the review questions and objectives, as well as potential implications and/or next steps.	
**FUNDING**			
Funding	22	Describe sources of funding for the included sources of evidence, as well as sources of funding for the scoping review. Describe the role of the funders of the scoping review.	

JBI = Joanna Briggs Institute; PRISMA-ScR = Preferred Reporting Items for Systematic reviews and Meta-Analyses extension for Scoping Reviews.

* Where *sources of evidence* (see second footnote) are compiled from, such as bibliographic databases, social media platforms, and Web sites.

† A more inclusive/heterogeneous term used to account for the different types of evidence or data sources (e.g., quantitative and/or qualitative research, expert opinion, and policy documents) that may be eligible in a scoping review as opposed to only studies. This is not to be confused with *information sources* (see first footnote).

‡ The frameworks by Arksey and O'Malley (6) and Levac and colleagues (7) and the JBI guidance (4, 5) refer to the process of data extraction in a scoping review as data charting.

§ The process of systematically examining research evidence to assess its validity, results, and relevance before using it to inform a decision. This term is used for items 12 and 19 instead of "risk of bias" (which is more applicable to systematic reviews of interventions) to include and acknowledge the various sources of evidence that may be used in a scoping review (e.g., quantitative and/or qualitative research, expert opinion, and policy document).

From: Tricco AC, Lillie E, Zarin W, O'Brien KK, Colquhoun H, Levac D, et al. PRISMA Extension for Scoping Reviews (PRISMAScR): Checklist and Explanation. Ann Intern Med. 2018;169:467–473. doi: 10.7326/M18-0850.

For peer review only - http://bmjopen.bmj.com/site/about/guidelines.xhtml

Additional file 2 – Search terms

Cinahl Plus search

(MH “Health Services Accessibility”) or (MH “Quality of Health Care”) or (MH “Help Seeking Behavior”) or (TI(“access to health service*” or “access to care” or “access to health care” or “health equity” or inequal* or equality or disparit* or unequal or gap* or gradient* or disadvantage*)) or (AB(“access to health service*” or “access to care” or “access to health care” or “health equity” or inequal* or equality or disparit* or unequal or gap* or gradient* or disadvantage*)) AND (MH “Primary Health Care”) or (MH “Primary Nursing”) or (MH “Physicians, Family”) or (MH “Family Practice”) or (TI(“primary health care” or “primary care” or “general practice*”) or “GP surger*” or “dentist*” or “general practitioner*” or “community pharmac*”) or (AB(“primary health care” or “primary care” or “general practice*” or “community pharmac*”) AND (MH Child) or (MH Adolescence) or (MH Infant) or (MH “Parent-Child Relations”) or (TI(child* or adolescen* or infant* or “young people” or youth or juvenile* or teenager* or student* or pupil* or “young adult*” or preschool*)) or (AB(child* or adolescen* or infant* or “young people” or youth or juvenile* or teenager* or student* or pupil* or “young adult*” or preschool*)) AND (MH "United Kingdom") or (MH England) or (MH Wales) or (MH Scotland) or (MH “Northern Ireland”) or (MH “Great Britain”) or (TX(UK or “United Kingdom" or England or Wales or Scotland or “Northern Ireland” or “N. Ireland” or “Great Britain”) AND (MH “Patient Satisfaction”) or (MH “Patient Preference”) or (MH “Health Knowledge”) or (TI(perceived or experience* or “attitude* to health” or facilitator* or enabler* or barrier* or promot* or inhibit* or view* or perspective*) or (AB(perceived or experience* or “attitude* to health” or facilitator* or enabler* or barrier* or promot* or inhibit* or view* or perspective*)

Filter: 2012-2022

Psycinfo search

((Health Care Access).sh. or (Health Care Utilization).sh. or (Health Disparities).sh. or (Help Seeking Behavior).sh. or (Health Care Seeking Behavior).sh. or (Quality of Care).sh. or (access to health service*).ti,ab. or (access to care).ti,ab. or (access to health care).ti,ab. or (health equity).ti,ab. or (inequal*).ti,ab. or (equality).ti,ab. or (disparit*).ti,ab. or (unequal).ti,ab. or (gap*).ti,ab. or (gradient*).ti,ab. or (disadvantage*).ti,ab.) AND ((Primary Health Care).sh. or (General Practitioners).sh. or (Family Physicians).sh. or (Pharmacy).sh. or (Dentists).sh. or (primary health care).ti,ab. or (primary care).ti,ab. or (general practice*).ti,ab. or (GP surger*).ti,ab. or (dentist*).ti,ab. or (general practitioner*).ti,ab. or (community pharmac*).ti,ab.) AND ((Child Behavior).sh. or (Early Adolescence).sh. or (Adolescent Psychology).sh. or (Parent-Child Relations).sh. or (child*).ti,ab. or (adolescen*).ti,ab. or (infant*).ti,ab. or (young people).ti,ab. or (youth).ti,ab. or (juvenile*).ti,ab. or (teenager*).ti,ab. or (student*).ti,ab. or (pupil*).ti,ab. or (young adult*) or (preschool).ti,ab.) AND ((United Kingdom).af. or (England).af. or (Wales).af. or (Scotland).af. or (Northern Ireland).af. or (Great Britain).af.) AND ((Client Satisfaction).sh. or (Client Attitudes).sh. or (Health Knowledge).sh. or (Treatment Barriers).sh. or (perceived).ti,ab. or (experience*).ti,ab. or (attitude* to health).ti,ab. or (facilitator*).ti,ab. or (enabler*).ti,ab. or (barrier*).ti,ab. or (promot*).ti,ab. or (inhibit*).ti,ab. or (view*).ti,ab. or (perspective*).ti,ab.)

Filter: 2012-2022

For peer review only - http://bmjopen.bmj.com/site/about/guidelines.xhtml

Web of Science Core Collection Social Sciences Citation Index (SSCI)

(TS=(“health care access” OR “help seeking behavior” OR “help seeking behaviour” OR “quality of care” OR “access to health service*” OR “access to care” OR “access to health care” OR “health equity” or inequal* or equality OR disparit* OR unequal OR gap* OR gradient* OR disadvantage*)) AND (TS=(“primary health care” OR “general practitioner*” OR “family physician*” OR “primary care” OR “general practice*” OR “GP surger*” OR dentist* OR “dental care” OR “community pharmac*”)) AND (TS=(“child behavior” OR “early adolescence” OR “adolescent psychology” OR “parent-child relation*” OR child* OR adolescen* OR infant* OR “young people” OR youth OR juvenile* OR teenage* OR student* OR pupil* OR “young adult*” OR preschool)) AND (ALL=(“United Kingdom” OR England OR Wales OR Scotland OR “Northern Ireland” OR “Great Britain”)) AND (TS=(“patient satisfaction” OR “patient preference*” OR “health knowledge” OR perceived OR experience* OR “attitude* to health” OR facilitator* OR enabler* OR barrier* OR promot* OR inhib* OR view* OR perspective*))

Filter: 2012-2022

SCOPUS

ALL((Health Services Accessibility OR "access to health services" OR "access to care" OR Health Equity OR "health equity" OR inequality OR inequalities OR equality OR disparity OR disparities OR unequal OR gap OR gaps OR gradients OR disadvantage OR health service utilisation OR health service utilisation OR "health resource utilisation" OR "health resource utilisation" OR health care seeking behaviour OR health care seeking behavior OR Health Care Quality OR Health Care Evaluation) AND (Primary Health Care OR "primary health care" OR "primary care" OR Primary Care Nursing OR Physicians Primary Care OR General Practice OR "general practice*" OR "GP surger*" OR General Practice Dental OR "dentist*" OR General Practitioners OR "general practitioner*" OR Community Pharmacy Services OR "community pharmac*" or "health visitor" OR pediatric care OR paediatric care) AND (Child or child* or Adolescent or adolescen* or Infant or infant* or "young people" or youth or juvenile* or teenager* or "young adult*" OR child* pre-school OR child* health) AND ("United Kingdom" OR England OR Wales OR Scotland OR "Northern Ireland" OR "N. Ireland") AND (Perception* OR perceived OR experience* OR Patient Satisfaction OR Patient Preference OR Attitude to Health OR Facilitator* OR enabler* OR barrier* OR Patient Acceptance of Health Care)) AND PUBYEAR > 2011 AND PUBYEAR < 2023 AND (LIMIT-TO (LANGUAGE,"English"))

Pubmed

Search: (("health services accessibility"[MeSH Terms] OR ("health"[All Fields] AND "services"[All Fields] AND "accessibility"[All Fields]) OR "health services accessibility"[All Fields] OR "access to health services"[All Fields] OR "access to care"[All Fields] OR ("health equity"[MeSH Terms] OR ("health"[All Fields] AND "equity"[All Fields]) OR "health equity"[All Fields]) OR "health equity"[All Fields] OR ("inequalities"[All Fields] OR "inequality"[All Fields] OR "inequities"[All Fields] OR "inequity"[All Fields]) OR ("inequalities"[All Fields] OR "inequality"[All Fields] OR "inequities"[All Fields] OR "inequity"[All Fields]) OR ("equal"[All Fields] OR "equaled"[All Fields] OR "equaling"[All Fields] OR "equalisation"[All Fields] OR "equalise"[All Fields] OR "equalised"[All Fields] OR "equalises"[All Fields] OR "equalising"[All Fields] OR "equalities"[All Fields] OR "equality"[All Fields] OR "equalization"[All Fields] OR "equalizations"[All Fields] OR "equalize"[All Fields] OR "equalized"[All Fields] OR "equalizer"[All Fields] OR "equalizers"[All Fields] OR "equalizes"[All Fields] OR "equalizing"[All Fields] OR "equalled"[All Fields] OR "equalling"[All Fields] OR "equally"[All Fields] OR "equals"[All Fields]) OR ("disparate"[All Fields] OR "disparately"[All Fields] OR "disparities"[All Fields] OR "disparity"[All Fields]) OR

For peer review only - http://bmjopen.bmj.com/site/about/guidelines.xhtml

("disparate"[All Fields] OR "disparately"[All Fields] OR "disparities"[All Fields] OR "disparity"[All Fields]) OR ("unequal"[All Fields] OR "unequally"[All Fields] OR "unequals"[All Fields]) OR "gap"[All Fields] OR "gaps"[All Fields] OR ("gradient"[All Fields] OR "gradient s"[All Fields] OR "gradients"[All Fields]) OR ("disadvantage"[All Fields] OR "disadvantageous"[All Fields] OR "disadvantageously"[All Fields] OR "disadvantages"[All Fields] OR "disadvantaging"[All Fields] OR "vulnerable populations"[MeSH Terms] OR ("vulnerable"[All Fields] AND "populations"[All Fields]) OR "vulnerable populations"[All Fields] OR "disadvantaged"[All Fields]) OR (("health services"[MeSH Terms] OR ("health"[All Fields] AND "services"[All Fields]) OR "health services"[All Fields] OR ("health"[All Fields] AND "service"[All Fields]) OR "health service"[All Fields]) AND ("statistics and numerical data"[MeSH Subheading] OR ("statistics"[All Fields] AND "numerical"[All Fields] AND "data"[All Fields]) OR "statistics and numerical data"[All Fields] OR "utilization"[All Fields] OR "utilisation"[All Fields] OR "utilisations"[All Fields] OR "utilise"[All Fields] OR "utilised"[All Fields] OR "utilises"[All Fields] OR "utilising"[All Fields] OR "utilities"[All Fields] OR "utility"[All Fields] OR "utilizations"[All Fields] OR "utilize"[All Fields] OR "utilized"[All Fields] OR "utilizer"[All Fields] OR "utilizers"[All Fields] OR "utilizes"[All Fields] OR "utilizing"[All Fields])) OR (("health services"[MeSH Terms] OR ("health"[All Fields] AND "services"[All Fields]) OR "health services"[All Fields] OR ("health"[All Fields] AND "service"[All Fields]) OR "health service"[All Fields]) AND ("statistics and numerical data"[MeSH Subheading] OR ("statistics"[All Fields] AND "numerical"[All Fields] AND "data"[All Fields]) OR "statistics and numerical data"[All Fields] OR "utilization"[All Fields] OR "utilisation"[All Fields] OR "utilisations"[All Fields] OR "utilise"[All Fields] OR "utilised"[All Fields] OR "utilises"[All Fields] OR "utilising"[All Fields] OR "utilities"[All Fields] OR "utility"[All Fields] OR "utilizations"[All Fields] OR "utilize"[All Fields] OR "utilized"[All Fields] OR "utilizer"[All Fields] OR "utilizers"[All Fields] OR "utilizes"[All Fields] OR "utilizing"[All Fields])) OR "health resource utilisation"[All Fields] OR "health resource utilisation"[All Fields] OR ("health care seeking behaviour"[All Fields] OR "patient acceptance of health care"[MeSH Terms] OR ("patient"[All Fields] AND "acceptance"[All Fields] AND "health"[All Fields] AND "care"[All Fields]) OR "patient acceptance of health care"[All Fields] OR ("health"[All Fields] AND "care"[All Fields] AND "seeking"[All Fields] AND "behavior"[All Fields]) OR "health care seeking behavior"[All Fields]) OR ("health care seeking behaviour"[All Fields] OR "patient acceptance of health care"[MeSH Terms] OR ("patient"[All Fields] AND "acceptance"[All Fields] AND "health"[All Fields] AND "care"[All Fields]) OR "patient acceptance of health care"[All Fields] OR ("health"[All Fields] AND "care"[All Fields] AND "seeking"[All Fields] AND "behavior"[All Fields]) OR "health care seeking behavior"[All Fields]) OR ("quality of health care"[MeSH Terms] OR ("quality"[All Fields] AND "health"[All Fields] AND "care"[All Fields]) OR "quality of health care"[All Fields] OR ("health"[All Fields] AND "care"[All Fields] AND "quality"[All Fields]) OR "health care quality"[All Fields]) OR (("delivery of health care"[MeSH Terms] OR ("delivery"[All Fields] AND "health"[All Fields] AND "care"[All Fields]) OR "delivery of health care"[All Fields] OR ("health"[All Fields] AND "care"[All Fields]) OR "health care"[All Fields]) AND ("evaluability"[All Fields] OR "evaluate"[All Fields] OR "evaluated"[All Fields] OR "evaluates"[All Fields] OR "evaluating"[All Fields] OR "evaluation"[All Fields] OR "evaluation s"[All Fields] OR "evaluations"[All Fields] OR "evaluative"[All Fields] OR "evaluatively"[All Fields] OR "evaluatives"[All Fields] OR "evaluator"[All Fields] OR "evaluator s"[All Fields] OR "evaluators"[All Fields]))) AND ("primary health care"[MeSH Terms] OR ("primary"[All Fields] AND "health"[All Fields] AND "care"[All Fields]) OR "primary health care"[All Fields] OR "primary health care"[All Fields] OR "primary care"[All Fields] OR ("primary nursing"[MeSH Terms] OR ("primary"[All Fields] AND "nursing"[All Fields]) OR "primary nursing"[All Fields] OR ("primary"[All Fields] AND "care"[All Fields] AND "nursing"[All Fields]) OR "primary care nursing"[All Fields] OR "primary care nursing"[MeSH Terms] OR ("primary"[All Fields] AND "care"[All Fields] AND "nursing"[All Fields])) OR ("physicians, primary care"[MeSH Terms] OR ("physicians"[All Fields] AND

"primary"[All Fields] AND "care"[All Fields]) OR "primary care physicians"[All Fields] OR ("physicians"[All Fields] AND "primary"[All Fields] AND "care"[All Fields]) OR "physicians primary care"[All Fields]) OR ("general practice"[MeSH Terms] OR ("general"[All Fields] AND "practice"[All Fields]) OR "general practice"[All Fields]) OR "general practice"[All Fields] OR "gp surger*"[All Fields] OR ("general practice, dental"[MeSH Terms] OR ("general"[All Fields] AND "practice"[All Fields] AND "dental"[All Fields]) OR "dental general practice"[All Fields] OR ("general"[All Fields] AND "practice"[All Fields] AND "dental"[All Fields]) OR "general practice dental"[All Fields]) OR "dentist*"[All Fields] OR ("general practitioners"[MeSH Terms] OR ("general"[All Fields] AND "practitioners"[All Fields]) OR "general practitioners"[All Fields]) OR "general practitioner*"[All Fields] OR ("community pharmacy services"[MeSH Terms] OR ("community"[All Fields] AND "pharmacy"[All Fields] AND "services"[All Fields]) OR "community pharmacy services"[All Fields]) OR "community pharmac*"[All Fields] OR "health visitor"[All Fields] OR ("pediatr care wilmington"[Journal] OR ("pediatric"[All Fields] AND "care"[All Fields]) OR "pediatric care"[All Fields]) OR (("paediatrics"[All Fields] OR "pediatrics"[MeSH Terms] OR "pediatrics"[All Fields] OR "paediatric"[All Fields] OR "pediatric"[All Fields]) AND "care"[All Fields])) AND ("child"[MeSH Terms] OR "child"[All Fields] OR "children"[All Fields] OR "child s"[All Fields] OR "children s"[All Fields] OR "childrens"[All Fields] OR "childs"[All Fields] OR "child*"[All Fields] OR ("adolescences"[All Fields] OR "adolescency"[All Fields] OR "adolescent"[MeSH Terms] OR "adolescent"[All Fields] OR "adolescence"[All Fields] OR "adolescents"[All Fields] OR "adolescent s"[All Fields]) OR "adolescen*"[All Fields] OR ("infant"[MeSH Terms] OR "infant"[All Fields] OR "infants"[All Fields] OR "infant s"[All Fields]) OR "infant*"[All Fields] OR "young people"[All Fields] OR ("adolescent"[MeSH Terms] OR "adolescent"[All Fields] OR "youth"[All Fields] OR "youths"[All Fields] OR "youth s"[All Fields]) OR "juvenile*"[All Fields] OR "teenager*"[All Fields] OR "young adult*"[All Fields] OR ("child*"[All Fields] AND ("child, preschool"[MeSH Terms] OR ("child"[All Fields] AND "preschool"[All Fields]) OR "preschool child"[All Fields] OR ("pre"[All Fields] AND "school"[All Fields]) OR "pre school"[All Fields])) OR ("child*"[All Fields] AND ("health"[MeSH Terms] OR "health"[All Fields] OR "health s"[All Fields] OR "healthful"[All Fields] OR "healthfulness"[All Fields] OR "healths"[All Fields]))) AND ("United Kingdom"[All Fields] OR ("england"[MeSH Terms] OR "england"[All Fields] OR "england s"[All Fields] OR "englands"[All Fields]) OR ("wales"[MeSH Terms] OR "wales"[All Fields] OR "wales s"[All Fields]) OR ("scotland"[MeSH Terms] OR "scotland"[All Fields] OR "scotland s"[All Fields]) OR "Northern Ireland"[All Fields] OR "n ireland"[All Fields]) AND ("perception*"[All Fields] OR ("perceivable"[All Fields] OR "perceive"[All Fields] OR "perceiver"[All Fields] OR "perceiver s"[All Fields] OR "perceivers"[All Fields] OR "perceives"[All Fields] OR "perception"[MeSH Terms] OR "perception"[All Fields] OR "perceived"[All Fields] OR "perceiving"[All Fields]) OR "experience*"[All Fields] OR ("patient satisfaction"[MeSH Terms] OR ("patient"[All Fields] AND "satisfaction"[All Fields]) OR "patient satisfaction"[All Fields]) OR ("patient preference"[MeSH Terms] OR ("patient"[All Fields] AND "preference"[All Fields]) OR "patient preference"[All Fields]) OR ("attitude to health"[MeSH Terms] OR ("attitude"[All Fields] AND "health"[All Fields]) OR "attitude to health"[All Fields]) OR "facilitator*"[All Fields] OR "enabler*"[All Fields] OR "barrier*"[All Fields] OR ("patient acceptance of health care"[MeSH Terms] OR ("patient"[All Fields] AND "acceptance"[All Fields] AND "health"[All Fields] AND "care"[All Fields]) OR "patient acceptance of health care"[All Fields]))) AND ((2012/1/1:2022/2/21[pdat]) AND (english[Filter]))*

Additional file 3: Quality assessment

To achieve 'high' quality, at least five MMAT criteria had to be met, with breadth and depth of analysis, for 'medium' at least four criteria had to be met, and all other studies were rated 'low'. For a judgement of 'high' relevance, studies had to describe, with breadth and depth, factors influencing primary care access and privilege participants' perspectives.

NB there were no randomized controlled trials in the studies so the MMAT questions for section 2 have been removed here.

**SCREENING QUESTIONS**	S1. Are there clear research questions?<br>S2. Do the collected data allow to address the research questions?
**1. QUALITATIVE STUDIES**	1.1. Is the qualitative approach appropriate to answer the research question?<br>1.2. Are the qualitative data collection methods adequate to address the research question?<br>1.3. Are the findings adequately derived from the data?<br>1.4. Is the interpretation of results sufficiently substantiated by data?<br>1.5. Is there coherence between qualitative data sources, collection, analysis and interpretation?
**3. NON-RANDOMIZED STUDIES**	3.1. Are the participants representative of the target population?<br>3.2. Are measurements appropriate regarding both the outcome and intervention (or exposure)?<br>3.3. Are there complete outcome data?<br>3.4. Are the confounders accounted for in the design and analysis?<br>3.5. During the study period, is the intervention administered (or exposure occurred) as intended?
**4. QUANTITATIVE DESCRIPTIVE STUDIES**	4.1. Is the sampling strategy relevant to address the research question?<br>4.2. Is the sample representative of the target population?<br>4.3. Are the measurements appropriate?<br>4.4. Is the risk of nonresponse bias low?<br>4.5. Is the statistical analysis appropriate to answer the research question?
**5. MIXED METHODS STUDIES**	5.1. Is there an adequate rationale for using a mixed methods design to address the research question?<br>5.2. Are the different components of the study effectively integrated to answer the research question?<br>5.3. Are the outputs of the integration of qualitative and quantitative components adequately interpreted?<br>5.4. Are divergences and inconsistencies between quantitative and qualitative results adequately addressed?

5.5. Do the different components of the study adhere to the quality criteria of each tradition of the methods involved?

		SCREENING QUESTIONS		1. QUALITATIVE STUDIES					Total MMAT	W1 - quality	W2 - relevance
First author	Year	S1	S2	1.1	1.2	1.3	1.4	1.5			
Ahmaro et al	2021	Yes	Yes	Yes	Yes	Yes	Yes	Yes	5	High	High
Alexakis et al	2015	Yes	Yes	Yes	Yes	Yes	Yes	Yes	5	High	High
Appleton et al	2022	Yes	Yes	Yes	Yes	Yes	Yes	Yes	5	High	High
Bosley et al	2021	Yes	Yes	Yes	Yes	Yes	Yes	Yes	5	Medium	Medium
Brigham et al	2012	Can't tell	Can't tell	Yes	Yes	Yes	No	Can't tell	3	Low	Low
Coleman-Fountain et al	2020	Yes	Yes	Yes	Yes	Yes	Yes	Yes	5	High	Low
Condon et al	2020	Yes	Yes	Yes	Yes	Yes	Yes	Yes	5	High	Low
Corry and Leavey	2017	Yes	Yes	Yes	Yes	Yes	Yes	Yes	5	High	High
Crouch et al	2019	Yes	Yes	Yes	Yes	Yes	Yes	Yes	5	High	Medium
Dando et al	2019	Yes	Yes	Yes	Yes	Yes	Yes	Can't tell	4	Low	Low
Davey et al	2013	Yes	Yes	Yes	Yes	Yes	Can't tell	Yes	4	Medium	High
Dickson	2015	Yes	Yes	Yes	Yes	Yes	No	No	3	Low	Medium
Diwakar et al	2019	Yes	Yes	Yes	No	Yes	Yes	Yes	4	Medium	Medium
Eskytė et al	2021	Yes	Yes	Yes	Yes	Yes	Yes	Yes	5	High	Low
Fox et al	2017	Yes	Can't tell	Yes	Yes	Yes	Yes	Yes	5	High	High
French et al	2020	Yes	Yes	Yes	Yes	Yes	Yes	Yes	5	Medium	High
Henderson and Rubin	2014	Can't tell	Yes	No	No	Can't tell	Yes	Can't tell	3	Low	Low
Ingram et al	2013	Yes	Yes	Yes	Yes	Yes	Yes	No	4	Medium	High
Jobanputra and Singh	2020	Yes	Yes	Yes	No	Yes	No	Yes	3	Low	Medium
Jones et al	2017	Yes	Yes	Yes	Yes	Yes	Yes	Yes	5	High	High
Lewney et al	2019	Yes	Yes	Yes	Yes	Yes	Yes	Yes	5	High	Medium
McDonagh et al	2019	Yes	Yes	Yes	No	Yes	Yes	Yes	4	Medium	High
Mughal et al	2021	Yes	Yes	Yes	Yes	Yes	Yes	Can't tell	4	Medium	High
Muirhead et al	2017	Yes	Yes	Yes	Yes	Can't tell	Yes	Yes	4	Medium	Low
Neill et al	2016	Yes	Yes	Yes	Yes	Yes	Yes	Yes	5	High	High
Neill et al	2015	Yes	Yes	Yes	Yes	Yes	Yes	Yes	5	High	High
O'Brien et al	2017	Yes	Yes	Yes	Yes	Yes	Yes	Yes	5	High	High
Ochieng	2020	Yes	Yes	Yes	Yes	Yes	Yes	Yes	5	High	Low

For peer review only

Rapley et al	2021	Yes	Yes	Yes	Yes	Yes	Yes	Yes	5	High	High
Redsell et al	2013	Yes	Can't tell	Yes	Can't tell	No	Yes	No	2	Low	Low
Roberts et al	2014	Yes	Can't tell	Yes	No	Yes	Yes	No	3	Low	High
Roberts and Condon	2014	Yes	Yes	Yes	No	Yes	Yes	Yes	4	Low	Low
Satherley et al	2021	Yes	Yes	Yes	Yes	Yes	Yes	Yes	5	High	High
Turnbull et al	2021	Yes	Yes	Yes	Yes	Can't tell	Yes	Yes	4	Medium	High
Turner et al	2012	Yes	Yes	Can't tell	Yes	Yes	No	Yes	3	Low	High
Williams et al	2014	Yes	Yes	Yes	Can't tell	Yes	Yes	Yes	4	Low	Low
Williams et al	2012	No	Yes	Yes	Yes	Yes	Yes	Yes	5	Medium	Medium

		SCREENING QUESTIONS		3. NON-RANDOMIZED STUDIES					Total MMAT	W1 - quality	W2 - relevance
First author	Year	S1	S2	3.1	3.2	3.3	3.4	3.5			
O'Brien et al	2019	Yes	Yes	Yes	Yes	Yes	Yes	Yes	5	High	Low
Usher-Smith et al	2015	Yes	Can't tell	Yes	Yes	Yes	Can't tell	Yes	4	Medium	Medium
Yassaee et al	2017	Yes	Yes	Yes	Yes	Yes	Can't tell	Yes	4	High	Low

		SCREENING QUESTIONS		4. QUANTITATIVE DESCRIPTIVE STUDIES					Total MMAT	W1 - quality	W2 - relevance
First author	Year	S1	S2	4.1	4.2	4.3	4.4	4.5			
Coyle et al	2013	Yes	Yes	Can't tell	No	Yes	Yes	No	2	Low	Low

		SCREENING QUESTIONS		5. MIXED METHODS STUDIES					Total MMAT	W1 - quality	W2 - relevance
First author	Year	S1	S2	5.1	5.2	5.3	5.4	5.5			
Crocker et al	2013	Yes	Yes	Yes	Yes	Yes	Yes	No	4	Low	Low
Fox et al	2015	Yes	Yes	Yes	Yes	Yes	No	Yes	4	Medium	Medium
Rashed et al	2022	Yes	Yes	Can't tell	Can't tell	Yes	Yes	Can't tell	2	Low	Medium
Rickett et al	2021	Yes	Yes	Yes	Yes	Yes	Yes	Yes	5	Medium	High
Salaheddin and Mason	2016	Yes	Yes	Can't tell	Yes	Yes	Can't tell	Yes	3	Low	Low
Wilson et al	2021	Yes	Yes	Yes	Yes	Yes	No	Can't tell	3	Low	Low

For peer review only

Additional file 4: data synthesis

Data were synthesised using framework analysis (Gale et al, 2013) in Microsoft Excel. There were four key stages in the analysis process: 1) framework analysis 1 – a descriptive extraction and categorisation, 2) framework analysis 2 – a conceptual analysis, 3) refinement of the themes, and 4) mapping the barriers for sub-populations.

1. Framework analysis 1: a descriptive extraction and categorisation of the data

One reviewer (LH) carried out inductive coding and created an initial framework (a structured template) to summarise/reduce the data to focus on facilitators/barriers to primary healthcare. Data was extracted into an excel worksheet, with each study a row and column a code. The framework was revised iteratively as data from each study was added; by the tenth study, most of the codes were identified and remained the same. The codes were organised under six overarching descriptive categories: accessibility (1), health care beliefs/knowledge/preferences of caregivers (2) and CYP (3), relationship with HCP (4), quality of diagnosis/treatment (5), HCP knowledge/skills/networks/priorities (6). Data from the remaining studies was extracted, with new codes added or revised as best fit the data. Two reviewers (LH and EA) then examined the codes for each category independently and discussed emerging themes.

Table 1: Initial data analysis framework and codes

Overarching category	Codes
**Accessibility**	Multiple opportunities to engage with HCP
	Co-location
	Accessible premises and opening times
	Having information in native or accessible language
	Availability of informational or health resources
	Communication about entry to the service
	Difficulties meeting system structures and requirements
	Difficulties meeting threshold for other services
	Variation/inconsistency of entry criteria
	Patient residential impermanence
	Different ways of being able to access HCP
	Wait times to be seen by HCP
	Reduced services
	Free health care.
	Stigma
	Discrimination
**Parents' beliefs, knowledge, or preferences**	Parents' perceiving access important for child's health
	Familiarity with condition
	Stigma around mental health
	Valuing health professionals' expertise
	Lack of trust in medication/services in UK
	Parents', families or communities' perceiving they had sufficient knowledge themselves

	Parent knowledge or confidence about services or how to access them
	Being able to arrange an emergency appointment
	Parent difficulties attending due to other responsibilities e.g. childcare
	Cultural expectations of health professional
	Concerns about wasting GP time
	Parents feeling misunderstood and not listened to by health system
	Parental perception of gender norms
	Parents feeling shame/judgement
**YPs' beliefs, knowledge, or preferences**	YP knowledge about services or how to access them
	Knowledge/familiarity of condition
	HCP same/opposite sex/gender preferences
	YP anxiety about seeking help from GP or from dentist
	YP perceiving whether HCP would take them seriously, without judgement and be interested in them
	Having choice
	Self-testing
	YP Cultural Expectations of HCP
	YP Self Reliance
	YP Sensitivity relating to family context
**Relationship with HCP**	Clear communication from health professional
	Health professional being friendly, approachable and reassuring (or not)/Personable Qualities of HCP
	Continuity of care
	Trusted relationship
	Confidentiality
	Duration of time in the consultation to listen to concerns
	Parents attending with YP
**Quality of diagnosis or treatment**	Accuracy of test result
	Timeliness of test result
**HCP knowledge, skills, networks, and priorities**	Health professionals having appropriate education and training
	Being able to undertake a good holistic assessment of family needs
	Health professionals' having local knowledge
	Health professionals having signposting, referral and co-ordination skills.
	Collaboration (or lack of) between services
	Priority given to health topic by professional
	HCP Professional Perceptions of Health Topic
	HCP perceptions of the individual

2. Framework analysis 2: a conceptual analysis and development of themes

After reflection and discussion between the reviewers, the data was re-organised conceptually following the journey of a caregiver or CYP from first noticing a health issue and deciding to seek help to attending a consultation and potentially being referred to secondary or adult services, influenced by the work of Ford et al's (2016). Ford et al outlined the following steps in access to primary care for socioeconomically disadvantage older people in rural areas: problem identified, decision to seek help, actively seek help, obtain appointment, get to the appointment, primary care interaction, and outcome.

For peer review only

Five higher-order themes were constructed from the data (see table 2). The data in the initial framework was re-organised, putting data relating to the new a-priori themes into separate Excel worksheets. Through inductive analysis of the data under each theme, new codes and sub-themes were constructed.

Table 2: Initial higher-order themes

Decision to access care
Reaching and entering services
Communication and trust between HCPs, caregivers and CYP
Gaps in HCP knowledge
General practice as a gatekeeper to, or a holding space for, secondary or adult care

To visualise whether any codes and themes were particularly pertinent for specific sub-populations with higher health needs, data was colour-coded: CYP from deprived areas, looked after children, non-White British CYP, CYP with SEN or disabilities, CYP with chronic conditions, and CYP with mental health problems. Where a study looked at two groups, text was coded in one colour and the cell background another. The sub-populations were selected from CYP target populations and focus clinical areas in the 'Core20Plus5', the national NHS England approach to support the reduction of health inequalities, though we included evidence for any chronic condition instead of the strategy's focus on asthma, diabetes, and epilepsy.

3. Refinement of the themes

The themes and sub-themes were mapped out visually in Powerpoint and discussed with the wider team. The decision to access care, reaching and entering services, and communication and trust formed a repeatable pattern of experiences that affected access to primary care. A consensus was reached among the team that the three sub-themes under "General practice as a gatekeeper…" fit within "Reaching and entering services" and "Communication and trust", and gaps in HCP knowledge impacted on communication and trust, and could be subsumed within that theme. These changes were made and final three over-arching themes were constructed.

4. Mapping the barriers for sub-populations of CYP with higher health needs

Sub-themes that were reported particularly for key sub-populations of interest (see table 3) were systematically mapped into a table.

Table 3: sub-populations of interest

**Author (year)**	CYP with mental health problems	CYP from deprived areas	CYP from non-White British communities	CYP with SEND	CYP with chronic health conditions	Looked after children
Ahmaro et al (2021)						
Alexakis et al (2015)			✓		✓	
Appleton et al (2022)	✓					
Bosley et al (2021)						
Brigham et al (2012)						
Coleman-Fountain et al (2020)	✓			✓		

For peer review only

Condon et al (2020)			✓			
Corry and Leavey (2017)	✓					
Coyle et al (2013)				✓		
Crocker et al (2013)						
Crouch et al (2019)	✓					
Dando et al (2019)			✓			
Davey et al (2013)						
Dickson (2015)		✓				
Diwakar et al (2019)					✓	
Eskytė et al (2021)		✓				
Fox et al (2017)		✓		✓		
Fox et al (2015)	✓					
French et al (2020)				✓		
Henderson and Rubin (2014)		✓				
Ingram et al (2013)						
Jobanputra and Singh (2020)	✓					
Jones et al (2017)						
Lewney et al (2019)						
McDonagh et al (2020)						
Mughal et al (2021)	✓					
Muirhead et al (2017)						✓
Neill et al (2016)*		✓	✓			
Neill et al (2015)*		✓	✓			
O'Brien et al (2019)	✓					
O'Brien et al (2017)	✓					
Ochieng (2020)			✓			
Rapley et al (2021)					✓	
Rashed et al (2022)						
Redsell et al (2013)						
Rickett et al (2021)						
Roberts et al (2014)	✓	✓				
Roberts and Condon (2014)						
Salaheddin and Mason (2016)	✓					
Satherley et al (2021)		✓				
Turnbull et al (2021)						
Turner et al (2012)						
Usher-Smith et al (2015)					✓	
Williams et al (2014)						✓
Williams et al (2012)			✓			
Wilson et al (2021)				✓		
Yassaee et al (2017)						

For peer review only

Author	Deciding to access care: *Identifying a health issue as worthy of attention from a HCP*	Deciding access care: *Consulting with family and social networks about symptoms*	Deciding to access care: *Surmounting stigma, experiences of discrimination, and embarrassment*
Ahmaro *et al.* (2021)			1
Alexakis *et al.* (2015)		1	
Appleton *et al.* (2022)			
Bosley *et al.* (2021)			
Brigham *et al.* (2012)			
Coleman-Fountain *et al.* (2020)	1		
Condon *et al.* (2020)			1
Corry and Leavey (2017)			1
Coyle *et al.* (2013)			
Crocker *et al.* (2013)			
Crouch *et al.* (2019)			
Dando *et al.* (2019)			1
Davey *et al.* (2013)			
Dickson (2015)			
Diwakar *et al.* (2019)			
Eskytė et al. (2021)	1		
Fox *et al.* (2017)		1	1
Fox *et al.* (2015)			
French *et al.* (2020)			1
Henderson and Rubin (2014)	1		
Ingram *et al.* (2013)	1	1	
Jobanputra and Singh (2020)			
Jones *et al.* (2017)			1
Lewney *et al.* (2019)			
McDonagh *et al.* (2020)			1
Mughal *et al.* (2021)	1		1
Muirhead *et al.* (2017)			
Neill *et al.* (2016)*	1	1	
Neill *et al.* (2015)*	1	1	
O'Brien *et al.* (2019)			
O'Brien *et al.* (2017)			1

For peer review only

Ochieng (2020)			1
Rapley *et al.* (2021)			
Rashed *et al.* (2022)			
Redsell *et al.* (2013)			
Rickett *et al.* (2021)			1
Roberts *et al.* (2014)			
Roberts and Condon (2014)	1		
Salaheddin and Mason (2016)			1
Satherley *et al.* (2021)			1
Turnbull *et al.* (2021)			1
Turner *et al.* (2012)			1
Usher-Smith *et al*. (2015)	1		
Williams et al. (2014)			
Williams *et al.* (2012)			1
Wilson et al. (2021)			
Yassaee *et al.* (2017)			

For peer review only

Reaching and entering services: *The supply of services*	Reaching and entering services: *Knowledge, confidence, and access to information about services and treatment*	Reaching and entering services: *Family-friendly healthcare settings*	Reaching and entering services: *Delayed or rejected referrals to secondary or adult care*
1			
		1	
1	1		
	1		
1			
	1		1
1	1		
1			
	1		
1	1		
	1		
		1	1
1	1		1
	1		
1			
			1
1	1		
1	1		
1	1		
1	1		
1			
1		1	
1	1		
			1
	1		1

For peer review only

1	1		1
1	1	1	
	1		
1		1	
1			
1			
1			
		1	
1			

For peer review only

Communication and trust: *Enabling CYP and caregivers to disclose their concerns*	Communication and trust: *Confidentiality and parental involvement*	Communication and trust: *HCP knowledge and competence*	Design
1			Qualitative; interviews
1		1	Qualitative; interviews
1		1	Qualitative; interviews
1			Qualitative; focus groups and interviews
			Qualitative; focus groups
			Qualitative; interviews
			Qualitative; focus groups
1	1	1	Qualitative; focus groups
		1	Quantitative; survey
			Mixed methods; survey and structured interviews
1			Qualitative; interviews
			Qualitative; interviews
1	1		Qualitative; interviews
			Qualitative; interviews
1		1	Qualitative; interviews
		1	Qualitative; interviews and focus groups
			Qualitative; interviews
1	1	1	Mixed methods; online survey and interviews
		1	Qualitative; interviews
			Qualitative; focus groups and interviews
		1	Qualitative; focus groups and interviews
	1	1	Qualitative; interviews
1			Qualitative; interviews
		1	Qualitative; interviews
1			Qualitative; interviews
1	1		Qualitative; interviews
			Qualitative; focus groups
1		1	Qualitative; focus groups and interviews
1			Qualitative; focus groups and interviews
			Quantitative; cross-sectional survey
		1	Qualitative; interviews

For peer review only

		1	Qualitative; focus groups
		1	Qualitative; interviews
1			Mixed; survey with closed and open questions
1		1	Qualitative; interviews
		1	Mixed; survey with closed and open questions
1	1	1	Qualitative; interviews
			Qualitative; interviews
			Mixed; survey with closed and open questions
1			Qualitative; interviews
1			Qualitative; interviews
1		1	Qualitative; interviews
			Quantitative; survey
1			Qualitative; interviews and routinely collected data
		1	Qualitative; focus groups
			Quantitative; telephone survey
1			Quantitative; cross-sectional survey

For peer review only

Who	Quality rating
YP	High
YP	High
YP and caregivers	High
Caregivers	Medium
HCPs	Low
YP	High
Caregivers	High
YP	High
HCPs	Low
Caregivers	Low
Caregivers	High
Caregivers	Low
YP	Medium
Caregivers	Low
Caregivers	Medium
HCPs	High
Caregivers	High
HCPs	Medium
HCPs, adults with ADHD,	Medium
HCPs, school staff, caregivers	Low
Caregivers	Medium
HCPs	Low
YP	High
HCPs	High
YP	Medium
YP	Medium
Caregivers	Medium
Caregivers	High
Caregivers	High
HCPs	High
HCPs	High

For peer review only

Caregivers and HCPs	High
Caregivers and HCPs	High
Caregivers and YP	Low
HCPs	Low
Caregivers	Medium
HCPs	Low
Caregivers	Low
YP	Low
Caregivers	High
YP	Medium
Caregivers	Low
Caregivers	Medium
HCPs, social workers, CYP,	Low
Caregivers	Medium
Optometric practices	Low
YP	High

For peer review only

BMJ Open

Access to primary care for children and young people (CYP) in the UK: a scoping review of CYP's, caregivers', and healthcare professionals' views and experiences of facilitators and barriers

Journal:	*BMJ Open*
Manuscript ID	bmjopen-2023-081620.R1
Article Type:	Original research
Date Submitted by the Author:	05-Mar-2024

Complete List of Authors:	Herlitz, Lauren; UCL GOS Institute of Child Health, NIHR Children and Families Policy Research Unit, Population, Policy and Practice<br>Ashford, Emily; UCL Social Research Institute, Thomas Coram Research Unit<br>Powell, Claire; UCL GOS Institute of Child Health, NIHR Children and Families Policy Research Unit, Population, Policy and Practice<br>Herbert, Kevin; Cambridge University, Public Health and Primary Care; Cambridge Research Methods Hub,<br>Morris, Stephen; University of Cambridge, Primary Care Unit<br>Woodman, Jenny; UCL Social Research Institute, Thomas Coram Research Unit

**Primary Subject Heading**:	Health services research
Secondary Subject Heading:	General practice / Family practice, Paediatrics, Public health
Keywords:	Adolescents < Adolescent, Health Equity, PAEDIATRICS, Health Services Accessibility, Primary Care < Primary Health Care

SCHOLARONE™
Manuscripts

I, the Submitting Author has the right to grant and does grant on behalf of all authors of the Work (as defined in the below author licence), an exclusive licence and/or a non-exclusive licence for contributions from authors who are: i) UK Crown employees; ii) where BMJ has agreed a CC-BY licence shall apply, and/or iii) in accordance with the terms applicable for US Federal Government officers or employees acting as part of their official duties; on a worldwide, perpetual, irrevocable, royalty-free basis to BMJ Publishing Group Ltd ("BMJ") its licensees and where the relevant Journal is co-owned by BMJ to the co-owners of the Journal, to publish the Work in this journal and any other BMJ products and to exploit all rights, as set out in our licence.

The Submitting Author accepts and understands that any supply made under these terms is made by BMJ to the Submitting Author unless you are acting as an employee on behalf of your employer or a postgraduate student of an affiliated institution which is paying any applicable article publishing charge ("APC") for Open Access articles. Where the Submitting Author wishes to make the Work available on an Open Access basis (and intends to pay the relevant APC), the terms of reuse of such Open Access shall be governed by a Creative Commons licence – details of these licences and which Creative Commons licence will apply to this Work are set out in our licence referred to above.

Other than as permitted in any relevant BMJ Author's Self Archiving Policies, I confirm this Work has not been accepted for publication elsewhere, is not being considered for publication elsewhere and does not duplicate material already published. I confirm all authors consent to publication of this Work and authorise the granting of this licence.

For peer review only

Access to primary care for children and young people (CYP) in the UK: a scoping review of CYP's, caregivers', and healthcare professionals' views and experiences of facilitators and barriers

Lauren Herlitz*, NIHR Children and Families Policy Research Unit**,** Population, Policy and Practice, UCL Great Ormond Street Institute of Child Health, 30 Guilford Street, London, WC1N 1EH, UK. ORCID: 0000-0003-2497-9041

Emily Ashford, Thomas Coram Research Unit, UCL Social Research Institute, 27 Woburn Square, London, UK.

Claire Powell, NIHR Children and Families Policy Research Unit**,** Population, Policy and Practice, UCL Great Ormond Street Institute of Child Health, 30 Guilford Street, London, WC1N 1EH, UK. ORCID: 0000-0002-6581-0165

Kevin Herbert, Primary Care Unit, Department of Public Health & Primary Care, University of Cambridge, Cambridge, UK. ORCID: 0009-0008-4354-7811

Steve Morris, Primary Care Unit, Department of Public Health & Primary Care, University of Cambridge, Cambridge, UK. ORCID: 0000-0002-5828-3563

Jenny Woodman, Thomas Coram Research Unit, UCL Social Research Institute, 27 Woburn Square, London, UK. ORCID: 0000-0002-9403-4177

*Corresponding author: l.herlitz@ucl.ac.uk

For peer review only

Abstract (max 300 words)

Objectives

To examine children and young people's (CYP), caregivers' and healthcare professionals' (HCP) views or experiences of facilitators and barriers to CYP access to UK primary care services to better understand healthcare inequity. To explore differences across CYP sub-populations with greater health needs: from deprived areas, identifying as ethnic minorities, with experiences of state care, special educational needs or disabilities, chronic conditions, or mental health problems.

Design

Scoping review.

Eligibility criteria

Included studies were in English, published 2012 – 2022 and reported: the views/experiences of CYP (0 – 25 years), caregivers, or HCPs about accessing UK primary care; using quantitative or qualitative empirical methods.

Data sources

Pubmed, CINAHL, Web of Science, Psycinfo and Scopus.

Results

We included 47 reports (46 studies). CYP/caregivers' decision to access care was facilitated by CYP/caregivers' or their family/friends' ability to identify a health issue as warranting healthcare attention. Barriers to accessing care included perceived stigma (e.g., being seen as a bad parent), embarrassment, and discrimination experiences. CYP and caregivers believed longer opening hours could facilitate more timely access to care. Caregivers and HCPs reported that delayed or rejected referrals to secondary or adult care was a barrier to having needs met, especially for CYP with poor mental health. CYP and caregivers in numerous studies emphasised the importance of communication and trust with HCPs, including taking their concerns seriously, being knowledgeable, and providing continuity of care for CYP. Common barriers reported across high-need sub-populations were caregivers needing knowledge and confidence to advocate for their child, gaps in HCP's knowledge, and a lack of connectedness between primary and secondary care.

Conclusions

Connecting general practices and community health workers/services, improving CYP/caregivers' understanding of common childhood conditions, addressing HCP's knowledge gaps in paediatric care, and integrated approaches between primary and secondary care may reduce inequity in access.

Strengths and limitations of this study

- The review was rigorously conducted and included quality appraisal.
- Mapping patterns of facilitators/barriers across different sub-populations with higher health needs was a strength of the review, revealing that access was affected by caregivers having to be

For peer review only - http://bmjopen.bmj.com/site/about/guidelines.xhtml

able to confidently advocate for their child's needs, multiple barriers existed for some groups, and there was a lack of evidence on access for looked after children.

- Studies in systematic reviews were not screened and we did not search for grey literature due to time and resources constraints.
- Workforce-related barriers, e.g. recruiting and retaining GPs, which affect both CYP and adult patients were not identified using our search terms.

Key words

Primary care; adolescents; children; access to healthcare; health equity

Introduction

Access to healthcare can be defined as the opportunity to identify healthcare needs, to seek, reach and use healthcare services, and to have healthcare needs met (1,2). Primary care access in childhood is important to ensure that children and young people (CYP) are vaccinated, reach developmental milestones, are safeguarded, and that acute and chronic conditions are identified and managed (3,4). Evidence also suggests improved access to primary care may reduce the escalation of health concerns, alleviating pressure on secondary care (5–7). The National Health Service's (NHS) long term plan in England highlights the role of primary care in reducing health inequalities and ensuring CYP (aged 0 – 25) have a strong start in life, in particular improving access for CYP with mental health problems, learning disabilities or autism (8). Unmet healthcare needs in adolescence are an independent predictor of poor adult health (9,10).

Recent evidence suggests that CYP access to primary care is inequitable. For example, UK cohort studies linked to routine health data found that CYP living in deprived areas were less likely to access primary care relative to their wealthier peers, and more likely to use acute care (11–13). Inequalities in CYP access to care may result from: variation in the supply of healthcare by area deprivation (14); differences in how conditions are identified and managed, for example, because of increased multimorbidity in CYP in deprived areas (15), or variation in healthcare professionals' (HCPs) expertise (16). Marginalised CYP and caregivers may not identify themselves as requiring health treatment, or may lack knowledge of available healthcare services and how to navigate complex healthcare systems (17,18). CYP's access is also affected by age and development, with younger children reliant on caregivers, and older adolescents and young adults seeking services independently (19).

Systematic reviews have been conducted on CYP and healthcare professional's (HCP) views of some specific healthcare services in the UK (20–22). In 2021, the National Institute for Health and Care Excellence (NICE) published guidelines on Babies, Children and Young People's experience of healthcare, which included an evidence review of healthcare access (including acute, primary and secondary care settings) (19). Focusing on CYP under 18, it found that a key barrier was a lack of information about when to access healthcare services, what services were available, and how CYP could be supported to access them. CYP also reported that they could avoid seeking help due to fear of being blamed, labelled or being embarrassed, or because they were unsure about the limits of confidentiality (19). Building on evidence from the NICE review, this study focused specifically on CYP's access to primary care, synthesising perspectives of CYP, caregivers, and HCP across primary

For peer review only - http://bmjopen.bmj.com/site/about/guidelines.xhtml

care services in order to deepen understanding of healthcare inequity, barriers to healthcare and how to address them, and looked in detail at facilitators and barriers for CYP with high health needs.

Method

Our methods were informed by rapid evidence review guidance (23). We pre-registered the review protocol in the Open Science Framework (https://osf.io/mfc3z). The study followed the Preferred Reporting Items for Systematic Reviews and Meta-Analyses extension for Scoping Reviews (PRISMA-ScR) statement (additional file 1).

Inclusion/exclusion criteria

We included a study if it:

- Reported the views or experiences of CYP (aged 0 to 25 years), caregiver (i.e., parent or carer), or HCPs on the facilitators and barriers to primary care access, including studies that examined primary care as a means of accessing secondary care.
- Was based in the UK.
- Used quantitative or qualitative empirical methods.
- Was published in English between 2012 and 2022.

We excluded studies that focused on access to school health services, access to primary care during the COVID-19 pandemic, or on the uptake of vaccinations/immunisations. We excluded systematic reviews.

Search strategy

We searched PubMed, CINAHL, Web of Science (Social Sciences Citation Index), Psycinfo and Scopus using free-text and index terms for the following concepts: healthcare access, primary care, CYP, UK, and facilitator and barriers (see additional file 2).

Document selection

We imported the search results into Rayyan software (https://www.rayyan.ai/) for de-duplication and screening. Five reviewers independently conducted title/abstract screening and twenty per cent (N=1334) were checked by a second reviewer. Two reviewers independently conducted full-text screening and 25% (N=36) were checked by a second reviewer. The first and second reviewers discussed disagreements until a consensus was reached, bringing in a third team member where necessary.

Data extraction

The following data were extracted: study sample/population; primary care setting; area of health care; study design/methodology; factors affecting primary care access. Data on access to primary care during the COVID-19 pandemic were not extracted.

Quality appraisal

Five reviewers assessed study quality using the Mixed Methods Appraisal Tool (24,25). No study was excluded based on quality, but study quality is acknowledged in the findings and quotes presented are from medium- and high-quality studies only. One reviewer assigned studies two 'weight-of-

For peer review only - http://bmjopen.bmj.com/site/about/guidelines.xhtml

evidence' ratings (26), one for quality and one for relevance to answering the review question, rated 'low', 'medium' or 'high' (see additional file 3). For a judgement of 'high' relevance, studies had to describe, with breadth and depth, factors influencing primary care access and privilege participants' perspectives.

Data synthesis

Data were synthesised using framework analysis (27) to systematically review and map the data from each study using a structured template (see additional file 4). After data were descriptively coded, a conceptual framework was applied following a patient pathway from a CYP/caregiver identifying a health issue and deciding to seek help, to organising an appointment, and attending a consultation, influenced by previous work (28). To visualise whether any codes and themes were pertinent for specific sub-populations with high needs, data were colour-coded for the following CYP groups: from deprived areas, experiences of state care (i.e. looked after children), identifying as ethnic minorities, with SEN or disabilities, with chronic conditions, and with mental health problems. Sub-populations were selected from CYP target populations and focus clinical areas in the 'Core20Plus5', the NHS England strategy for reducing health inequalities (29). Sub-themes reported for these sub-populations were systematically mapped.

Patient and public involvement

Patients and/or the public were not involved in the design, conduct or reporting of this review.

Results

Of the 6,671 unique title/abstracts were generated from database searching in February 2022, 47 reports (of 46 studies) met the inclusion criteria (see figure 1).

Study characteristics

Study design/methods

Most studies were qualitative using interviews (n=25), or focus groups (n=6), or focus groups and interviews (n=5). All quantitative studies used cross-sectional surveys (n=5), whilst mixed-method studies used surveys that contained open and closed questions (n=5) (see table 1).

CYP age focus and health topic

Ten studies (22%) focused on CYP under 5 years, 12 (26%) were about CYP between the ages of 0 and 15 years, 10 (22%) focused on young people (YP) aged 16 to 25 years, and the rest focused on a range of different ages between 0 and 25 years (see supplementary table).

Thirteen studies (28%) were related to CYP access for non-specific health conditions; 11 (24%) were about CYP with mental health conditions; 8 (17%) were about CYP's oral health; 4 (9%) focused on CYP with chronic health conditions; 4 (9%) were about CYP with physical health conditions; 4 (9%) focused on YP's sexual health; 1 (2%) was on help-seeking for children's gender identity; and 1 (2%) examined CYP eye care from optometry practices (see table 1).

For peer review only - http://bmjopen.bmj.com/site/about/guidelines.xhtml

Study participants

Most studies invited either caregivers (n=18), young people (aged 11+ years) (n=11), or HCPs (n=10) to participate; seven studies included more than one type of participant and one study surveyed optometry practices. More than half of studies focused on CYP in general (n=28); the rest focused on a particular sub-population(s) (see supplementary table).

Primary healthcare setting

The following healthcare settings were studied (note, several studies covered multiple settings): general practice (n=27), health visiting (n=8), dental care (n=6), overall primary care (excluding dental care or optometry) (n=4), pharmacy services (n=3), optometry (n=1), walk-in centres (n=1) or sexual health clinics (n=1) (see table 1).

Study quality and relevance

Ten studies (22%) were rated high on both quality and relevance (see additional file 3). Studies on CYP with chronic conditions and sexual health were rated higher on quality and relevance; while half of oral health studies, and the only optometry study, were rated low on quality.

Insert figure 1 about here.

For peer review only

Table 1: Characteristics of studies included

**Author (year) (citation) *location***	***Primary healthcare setting:* main focus of study**	**Design⁺**
Ahmaro *et al.* (2021) (30) *England*	*Pharmacies*: sexual health and chlamydia testing and chlamydia treatment.	Qual; I
Alexakis *et al.* (2015) (31) *England*	*General practice:* needs of YP with inflammatory bowel disease from black & ethnic minority communities.	Qual; I
Appleton *et al.* (2022) (32) *England*	*General practice:* receiving primary care support after child and adolescent mental health services.	Qual; I
Bosley *et al.* (2021) (33) *England*	*General practice and health visiting*: the accessibility and expertise of HCPs.	Qual; FG & I
Brigham *et al.* (2012) (34) *England*	*Health visiting*: health visitors' (HVs) perceptions of their role and skills, sharing expertise, and work with other agencies.	Qual; FG
Coleman-Fountain *et al.* (2020) (35) *n/k*	*General practice:* exploring how autistic young adults understand and manage mental health problems.	Qual; I
Condon *et al.* (2020) (36) *England*	*General practice and health visiting*: using services post-migration from Romania, Poland, Pakistan or Somalia.	Qual; FG
Corry and Leavey (2017) (37) *N. Ireland*	*General practice*: adolescents' attitudes to consulting their GP about psychological problems.	Qual; FG
Coyle *et al.* (2013) (38) *N. Ireland & Scotland*	*Dental care*: HCP's willingness to treat adolescents with learning disabilities (LD) in primary dental care.	Quant; S
Crocker *et al.* (2013) (39) *Wales*	*General practice:* consulting a GP before the day of hospital presentation with pneumonia or empyema.	Mixed; S & I
Crouch *et al.* (2019) (40) *England*	*General practice:* seeking help and accessing specialist treatment for childhood anxiety	Qual; I
Dando *et al.* (2019) (41) *England*	*General practice:* healthcare experiences of Albanian survivors of modern slavery and sexual exploitation	Qual; I
Davey *et al.* (2013) (42) *England*	*General practice and walk-in centres*: the needs and experiences of young adults of primary healthcare services.	Qual; I
Dickson (2015) (43) *N. Ireland*	*Dental care*: parents' perceptions of factors influencing dental registrations of children living within a Sure Start area.	Qual; I
Diwakar *et al.* (2019) (44) *England*	*General practice:* understanding parent experiences with paediatric allergy pathways.	Qual; I
Eskytė *et al.* (2021) (45) *England*	*Health visiting*: organisational factors that obstruct HVs from speaking to parents of babies about oral health	Qual; I & FG
Fox *et al.* (2017) (46) *England*	*General practice and health visiting*: health, education, and social care services support for CYP with autism.	Qual; I
Fox *et al.* (2015) (47) *England*	*General practice*: identifying barriers to and enablers for discussing self-harm with YP.	Mixed; online S & I
French *et al.* (2020) (48) *UK*	*General practice:* exploring the primary care experiences of referral and management of ADHD	Qual; I
Henderson and Rubin (2014) (49) *England*	*Dental care*: an oral health promotion initiative to improve access for pre-school children in deprived communities.	Qual; FG & I
Ingram *et al.* (2013) (50) *n/k*	*General practice:* support/information needs when children have respiratory tract infections with a cough	Qual; FG & I
Jobanputra and Singh (2020) (51) *England*	*General practice:* exploring GPs' views on the management of adolescents with mental health disorders	Qual; I
Jones *et al.* (2017) (52) *England*	*General practice*: receiving chlamydia testing with condoms, contraceptive information, and HIV testing.	Qual; I

For peer review only - http://bmjopen.bmj.com/site/about/guidelines.xhtml

Lewney *et al.* (2019) (53) *England*	*Health visiting:* HVs views about providing oral health advice and dealing with dental issues	Qual; I
McDonagh *et al.* (2020) (54) *UK*	*General practice*: barriers to chlamydia testing and potential intervention functions and implementation strategies.	Qual; I
Mughal *et al.* (2021) (55) *England*	*General practice*: help-seeking behaviours, GP care, and healthcare access for YP who self-harm.	Qual; I
Muirhead *et al.* (2017) (56) *England*	*Dental care:* foster carers' oral health knowledge, attitudes, and experiences of managing foster children's oral health.	Qual; FG
Neill *et al.* (2016)***** (57) *England*	*Primary care (all except dental and optometry)*: making decisions during acute childhood illness at home.	Qual; FG & I
Neill *et al.* (2015)***** (58) *England*	*Primary care (all except dental and optometry)*: information resources for decision-making in acute childhood illness at home.	Qual; FG & I
O'Brien *et al.* (2019) (59) *England*	*General practice*: identifying, managing, and accessing specialist services for anxiety disorders.	Quant; S
O'Brien *et al.* (2017) (60) *England*	*General practice:* identification, management, and access to specialist services for anxiety disorders.	Qual; I
Ochieng (2020) (61) *England*	*Health visiting*: factors that either influence healthy weight in black African children.	Qual; FG
Rapley *et al.* (2021) (62) *England*	*Primary care (all except optometry):* experiences of care, from initial symptoms to initial referral to paediatric rheumatology.	Qual; I
Rashed *et al.* (2022) (63) *England*	*Pharmacy and general practice:* exploring the experiences, barriers and recommendations of caregivers and YP regarding the use of community pharmacies for children.	Mixed; S
Redsell *et al.* (2013) (64) *England*	*Health visiting*: the beliefs and practices of UK HVs concerning infants at risk of developing obesity.	Qual; I
Rickett *et al.* (2021) (65) *Scotland, Wales, & England*	*General practice:* healthcare expectations and experiences of caregivers seeking support for their gender diverse children	Mixed; S
Roberts *et al.* (2014) (66) *England*	*General practice*: GPs' experiences and views of consulting with adolescents with psychological difficulties.	Qual; I
Roberts and Condon (2014) (67) *England*	*Dental care:* exploring parental attitudes to pre-school oral health.	Qual; I
Salaheddin and Mason (2016) (68) *UK*	*General practice:* exploring the barriers to accessing mental health support among young adults.	Mixed; S
Satherley *et al.* (2021) (69) *England*	*General practice*: how mothers living in deprived neighbourhoods support their children with health conditions.	Qual; I
Turnbull *et al.* (2021) (70) *England*	*Pharmacy and sexual health clinic:* accessing emergency contraception pills	Qual; I
Turner *et al.* (2012) (71) *England*	*General practice*: views and experiences of primary care as a treatment setting for childhood obesity.	Qual; I
Usher-Smith *et al.* (2015) (72) *England*	*General practice and secondary care*: Explored the pathway to diagnosis of type 1 diabetes.	Quant; S
Williams *et al.* (2014) (73) *England & Wales*	*Dental care*: the impact of a community-based dental care pathway on children's dental care entering residential or foster care.	Qual; I & RDC
Williams *et al.* (2012) (74) *England*	*Not specified (preventative primary care services)*: African and African-Caribbean fathers' beliefs about fatherhood, health and preventive primary care services.	Qual; FG
Wilson *et al.* (2021) (75) *England*	*Optometric practices*: accessibility of eye care for children with typical development and those with autism.	Quant; S
Yassaee *et al.* (2017) (76) *England*	*General practice*: GP experiences, associations between poor reported GP experience and physical and mental health measures and service utilisation.	Quant; S

[+]Qual = qualitative, Quant = Quantitative, Mixed = Mixed Method, I = Interviews, FG = Focus Groups, S = Survey, RDC = routine data collection

For peer review only - http://bmjopen.bmj.com/site/about/guidelines.xhtml

For peer review only

*Reports are from the same study.

For peer review only

Facilitators and barriers in CYP access to primary care

We constructed three overarching themes on CYP and caregivers' access to primary care: deciding to access care; reaching and entering services; and communication and trust between HCPs, caregivers and CYP (see figure 2). Additional file 5 provides a table of themes by study.

Insert figure 2 about here.

1. Deciding to access care

Multiple studies examined caregivers' and YPs' decisions to access healthcare. We constructed three sub-themes: identifying a health issue as worthy of attention from a HCP (n=9); consulting with family and social networks about symptoms (n=5); and surmounting stigma, experiences of discrimination, and embarrassment (n=17).

a. Identifying a health issue as worthy of attention from a HCP

Three studies (of medium/high-quality, in four reports) reported that a YP or caregiver would only seek help if they considered their symptoms serious, wanting to avoid burdening health system resources (50,57,58,72). As well as assessing severity, caregivers considered the familiarity of the illness, their child's level of distress, and whether symptoms were worsening and/or persisting (50,57,58,72). First-time parents were more likely to access care as childhood illnesses were unfamiliar, making it difficult for parents to judge severity (57). The basis of YP's (aged 16+) decision-making for mental health concerns was similar to those of caregivers; they would consider seeking help if their distress was severe and enduring, and was felt to be beyond self-management (35,55).

In three studies of pre-school children, (one high-quality, two low-quality), two of which focused of CYP from deprived areas, HVs and parents reported oral health was of low priority in comparison to assessing children's physical health and developmental milestones (45,49,67).

b. Consulting with family and social networks about symptoms

Five studies (of medium/high-quality, in six reports) reported that parents and YP utilised their family and social networks, as well as material resources (e.g. websites, leaflets) to confirm their decision to consult (31,46,50,57,58,69). Contradictory advice or encouragement from family/friends to seek help contributed to a decision to consult (50,69).

In three high-quality studies (in four reports), caregivers from South Asian, Gypsy/Travelling, and Somali communities, and YP from Black and ethnic minority groups reported that they would defer to children's grandparents, extended family or community members for advice, and relied upon their children or local community to relay information if they could not read and write in English (31,46,57,58). Two of the studies identified that if the community were unfamiliar with the syndrome/illness studied (or it was stigmatised), families could encounter advice not to seek help, dismissive responses to diagnosis, or inappropriate efforts to treat the condition (31,46):

> *"Some of the people say, "Why are you saying something silly like this?" He's a child, he will grow out of it [autism]. A lot of children can't talk at the normal age, why don't you wait? Don't go to the doctors. He will grow out of these things."* (Caregiver, (46)).

For peer review only - http://bmjopen.bmj.com/site/about/guidelines.xhtml

For peer review only

c. Surmounting stigma, experiences of discrimination, and embarrassment

Stigma, discrimination, and embarrassment were reported as barriers to help-seeking. Four studies (of mixed quality) highlighted that parents could feel judged on their parenting, labelled as 'pushy parents', or blamed for their child's condition. This was found in studies of mothers of low socio-economic status, children with ADHD, gender diverse children, and those experiencing childhood obesity (48,65,69,71).

> *"I'm on income support, so asking me to feed her quinoas, avocados and vegetables, that's just not … I can barely get the milk for the tea. And then I have five other children, how am I going to measure the powder every meal?" (*Caregiver, (69))

Stigma and discrimination experienced by ethnic minorities and migrants were barriers identified in four studies (of mixed quality) (36,41,61,74). For example, caregivers being sent away or ignored (41) or labelled as 'aggressive' when trying to resolve misunderstandings with HCPs (74). Two studies (of medium/high-quality) reported that African/African-Caribbean fathers and migrant caregivers perceived preventative services as part of a government surveillance system, indicating distrust of services (36,74).

Stigma related to mental health felt by YP or their caregiver could be a barrier to seeking help from a GP, as reported in four studies (of mixed quality), (46,55,60,68), two of which suggested that mental health stigma was more common among ethnic minorities (46,60). Believing that they would not be taken seriously, or fears that they would not have a say in their treatment, were barriers to seeking mental health support reported by YP (aged 13+) (37,55,68).

Embarrassment was a common barrier for YP (aged 16+) seeking sexual health care, noted in four studies (of medium/high-quality) (30,52,54,70). YP reported being concerned about being seen by family/friends or judged by staff, feeling ashamed to be accessing emergency contraception, and embarrassed by the testing procedure itself. In one high-quality study, YP felt HCPs might make assumptions about promiscuity or judge them on the basis of their sexuality, affecting their willingness to be tested in general practice (54).

2. Reaching and entering services

After caregivers or YP decided healthcare support was needed, organising an appointment and entering services was the next step to access. We identified four sub-themes among the many studies exploring this theme: the supply of services (n=22); caregivers' and YP's knowledge, confidence, and access to information about services and treatment (n=18); family-friendly healthcare settings (n=6); and delayed or rejected referrals to secondary or adult care (n=7).

a. The supply of services: timeliness, location, choice, and availability

Caregivers and YP reported that longer GP, pharmacy and sexual health clinic opening hours could facilitate more timely access to care in seven studies (of mixed quality) (30,39,54,58,63,70). Caregivers of young children (under 5) noted it could be difficult to attend (or phone for) appointments early in the morning when children were getting ready for school, or at children's bedtime, in one high-quality study (57). Caregivers were willing to seek advice and treatment from nurses, pharmacists and NHS Direct (instead of a GP) if they wanted to be seen quickly, and/or the illness was considered common and/or mild (33,50,58,63). Two studies (of medium/high-quality)

For peer review only - http://bmjopen.bmj.com/site/about/guidelines.xhtml

found that parents sought out a private diagnosis to gain more timely access to care (for ADHD and for juvenile idiopathic arthritis) and to evade GP ‘gatekeeping’ (48,62).

Healthcare practices that were within walking distance of patients’ homes or work, or on bus routes could facilitate access, as reported by caregivers and YP (30,33,39,52), as could co-locating health and other children’s services, according to HVs and caregivers (33,45). Choice of healthcare settings and professional was salient in sexual health studies; YP (aged 16+) appreciated options for seeking testing and advice (online, pharmacy, GP, sexual health clinic) where privacy/discretion was a key consideration, and some YP preferred to speak to a staff member with the same gender identity (30,42,52,54,70).

In terms of service availability, participants from multiple studies reported long waiting times to see a GP (39,42,52,55,63,72). Reduced engagement with HVs as a result of cuts to provision was noticed by caregivers and HVs in two studies (of medium/high-quality) (33,45). HVs also noted the lack of NHS dentists in the deprived areas in which they worked (45). Three studies (of low/medium-quality) found caregivers had received conflicting information from dental practices about the age for registering children (43,56,67). One low-quality study noted that strict non-attendance and de-registration policies to manage resources in dentistry adversely affected looked after children, who often had a history of low dental attendance, poor diet and oral hygiene before care entry, and higher dental care anxiety (73):

> *“They haven't been to the dentist for a long time...then they are suddenly faced with a dental appointment, and often they are fine, and then the day before or the day of the appointment, they categorically refuse to go.”* (Caregiver, (73)).

One study (of low-quality) found optometry practices varied in whether they thought young children (under 5) should be examined by a GP or an optometrist (75).

b. Caregivers’ and YP’s knowledge, confidence, and access to information about services and treatment

Studies (of mixed quality) reported variation in caregivers' and YP's knowledge of appointment systems, though YP were more often inexperienced in accessing care (33,36,42,46,49,55). In a high-quality study on Somali migrants’ access to care for CYP with autism, caregivers reported feeling overwhelmed by the complexity of the health and education system, and the lack of clarity around the purpose of appointments and professional roles (46). Caregivers of CYP with complex needs and HCPs reported that parents having the confidence to persist in asking for support for their child helped them to gain timely access to care and appropriate referrals to secondary care, as noted in multiple studies (of medium/high-quality) relating to CYP with chronic conditions, mental health problems, ADHD, and gender diversity (40,44,48,60,62,65):

> *“…if I felt a child was, not necessarily needing secondary care but the family were overly concerned and were pushing for a referral [for anxiety], I would probably [go] along with that.’* (GP, (60))

A lack of clear, visible information about what services were offered at the GP and pharmacy was reported by YP (aged 16+) and caregivers in four studies (of a mix of quality) (42,52,54,63). Two high-

For peer review only - http://bmjopen.bmj.com/site/about/guidelines.xhtml

quality studies identified that confusion over who was responsible for organising an interpreter was a barrier to dental and GP care (46,53). Some caregivers of young children reported that they liked to receive practical resources and hard copies of information about child health that they could refer back to, reported in two high-quality studies focused on CYP from deprived areas (45,58). YP (aged 16+) reported they would like demonstration videos via websites alongside instructions for self-testing in one medium-quality sexual health study (54).

c. Family-friendly healthcare settings

The healthcare setting itself could be a barrier to help-seeking. It was stressful for caregivers of young children to wait with their child or with other children in tow, a problem particularly affecting single parents and parents without easy access to childcare (57). In some practices, the physical environment could be difficult to navigate with a buggy (67). Signalling that healthcare settings were child- and parent-friendly, for example, by putting posters or toys in the waiting area for younger children (63,67), or being warm and approachable at the reception desk, was appreciated by caregivers and YP, particularly caregivers who were not fluent in English or YP who were struggling with their mental health (32,47,57). One medium-quality study flagged that the fathers in their study perceived child health services as designed for women, rather than men (74).

d. Delayed or rejected referrals to secondary or adult care

Delayed or rejected referrals to secondary or adult care was a barrier to CYP having their health needs met. Three studies (of medium/high-quality) about care for anxiety, ADHD, and juvenile idiopathic arthritis reported several reasons for GPs delaying referrals: a decision to 'wait and see' to see if more evidence materialised, the assumption that symptoms were the result of another non-medical cause, or were due to a pre-existing known condition (40,48,62). The feeling of being 'passed around' services was recounted by both HCP and caregivers of CYP with these conditions (48,60,62).

Both caregivers' and HCPs described frustration over the care of CYP's mental health and ADHD resulting from: long waiting lists for Child and Adolescent Mental Health Services (CAMHS); rejected referrals to CAMHS due to high thresholds, GPs lack of knowledge about available mental health and ADHD services, or what information is needed to obtain a successful referral; or lack of clear care pathways, reported in five studies (of mostly medium/high-quality) (47,48,51,59,60).

3. Communication and trust between HCPs, caregivers and CYP in consultations

Once a consultation with a HCP professional was arranged, accessing the help CYP needed depended upon communication and trust with HCPs. We constructed three sub-themes from multiple studies: enabling CYP and caregivers to disclose their concerns (n=22); managing confidentiality and parental involvement (n=6); and HCP knowledge and competence (n=20).

a. Enabling CYP and caregivers to disclose their concerns

A 2014 national survey of adolescents in England found that only 54% of YP who had visited the GP in the last year felt able to talk to them about personal matters (76). Numerous studies highlighted that the quality of patient encounters with HCPs impacted on their willingness to disclose information. Caregivers and YP across many studies identified the same HCP attributes that would

For peer review only - http://bmjopen.bmj.com/site/about/guidelines.xhtml

help them to share their concerns: HCPs should be reassuring, trustworthy, and knowledgeable (30,33,37,40,54,55,63,70,73).

> '*His [the GP's] patience and lack of judgement was amazing, just to listen to my experiences of what happens for emotionally when I'm self-harming… it was incredible*.' (YP, 22 years, (55))

HCPs showing that they were listening and taking CYP's symptoms seriously was very important. Displaying scepticism or disbelief of CYP's ailments led to caregivers and CYP feeling that CYP's needs had not been met (31,37,40,42,55,57,58,69).

> *"I went back there (GP practice) quite a few times and… my GP was trying to convince me that it [Crohn's Disease] was in my head and I was just imagining it."* (YP, 24 years, (31))

Two studies (in three reports) of caregivers from deprived areas (one of which also focused on minority ethnic groups) highlighted that parents felt a sense of powerlessness and inferiority in the provider-patient interaction which could prevent them from sharing relevant information or leave them feeling unsupported (57,58,69).

Continuity of care was considered valuable in building a positive, trusting relationship between YP/caregivers and HCPs (33,42,44,52,57,63,64,73), and was particularly vital for CYP with mental health concerns (32,37,47,55,66). YP, caregivers, and HCP, noted that in discussions about sensitive matters, such as mental health, HCP should be careful about language used and help-seeking should be framed as a healthy and positive behaviour (40,47,52,54,64,71). Information-giving should be tailored to the individual, for example, YP attending a sexual health service might need more support on their first visit (42,70). Participants of all types in multiple studies reported that more consultation time was needed for sensitive subjects, notably mental health, or when support needs were high (37,42,55,64,66,69,70,73).

b. Confidentiality and family involvement

YP, particularly those with mental health problems, expressed concern that information about them would be shared with family or other professionals without their consent, as reported in four mixed-quality studies (37,42,55,66). Parents could be a facilitator or a barrier to mental health consultations with YP: parents could facilitate access by encouraging them to attend and supporting their account; or parents could inhibit the YP from sharing information if the YP did not want to upset them, if they wanted something different from their parent, or their parental relationship was part of the problem (47,51,66).

c. HCP knowledge and competence

Studies highlighted multiple areas where HCPs lacked sufficient expertise to manage care (see table 2). GP management of CYPs' mental health was the knowledge and competency gap most often reported by YP, caregivers and HCPs. It included: presentation of different conditions; how to enable CYP to share their concerns; knowledge of available treatment options and CAMHS services; and managing potential risks of approaching sensitive topics in front of family members (see table 2). If there was a delay or unsuccessful referral in accessing secondary or adult care (see "Delayed or reject referrals"), then the GP remained the (non-expert) provider of care in the interim

For peer review only - http://bmjopen.bmj.com/site/about/guidelines.xhtml

(32,48,51,60,66). Managing physical changes from puberty while waiting for specialist care for gender diversity was a new area where expertise was required (65).

YP and caregiver trust in HCPs' expertise could diminish when repeated consultations resulted in little improvement or misdiagnosis, and was a barrier to seeking further help from primary care, as reported in multiple studies (of predominantly medium/high-quality), three focusing on CYP with chronic health conditions and three on ethnic minority groups (31,44,50,57,62,74). Thus, experiences of communication and trust affected the decision to access care in the future.

Barriers affecting equitable access to care

Specific barriers affecting access to care across themes were mapped for several sub-populations with known higher health needs (see table 3). Multiple trust-related barriers were reported by ethnic minority caregivers and YP resulting from negative past experiences with unfriendly staff, or unsatisfactory support or diagnosis, combined with a need for more accessible and culturally appropriate health information. Many barriers to seeking mental health support were identified by YP, caregivers and HCP, including: a lack of patient and HCP awareness of treatment options, and organisational processes which diminished relationship-building between YP and HCPs (e.g. short appointments, less continuity of care). Commons barriers reported across sub-populations were caregivers needing to have the knowledge or confidence to ask for the help they needed or to challenge a HCP whose advice they disagreed with, gaps in HCP knowledge and in communication between primary and secondary care.

For peer review only

For peer review only - http://bmjopen.bmj.com/site/about/guidelines.xhtml

Table 2: Reported variability/gaps in HCP knowledge

Variability/gaps in HCP knowledge in treating CYP	Reported by	References	Quality rating of references
**General practitioners**			
Mental health: presentation of different conditions; enabling CYP to share their concerns; knowledge of available treatment options and CAMHS services; managing potential risks of approaching sensitive topics in front of family members.	CYP<br>HCPs<br>Caregivers	(32,37,47,51,60,66)	3 High,<br>1 Medium,<br>2 Low
Allergy management and referrals to secondary care.	Caregivers	(44)	1 Medium
The needs of primary-aged gender diverse children and support services available.	Caregivers	(65)	1 Medium
Identifying and managing juvenile arthritis.	HCP	(62)	1 High
ADHD aetiology, identification, diagnosis, referral processes, services available.	HCP<br>CYP<br>Caregivers	(48)	1 Medium
The experiences and needs of families from ethnic minority groups.	Caregivers<br>CYP	(31,74)	1 High,<br>1 Medium
How to sensitively and effectively address childhood obesity, particularly when caregivers have struggled with their own weight.	Caregivers	(71)	1 Low
**Dentistry**			
Managing children with learning difficulties.	HCP	(38)	1 Low
**Health visiting teams**			
Oral health promotion, culturally specific oral health guidance, knowledge of local dentistry services.	HCP	(45,53)	2 High
Culturally specific advice concerning feeding practices.	Caregivers<br>HCP	(61)	1 High
How to address childhood obesity.	HCPs	(64)	1 Low

For peer review only

For peer review only - http://bmjopen.bmj.com/site/about/guidelines.xhtml

Table 3: Barriers to accessing care for sub-populations of CYP

Sub-population (no. of studies)	Reported barriers to access
CYP with mental health problems (n=11)	• *Decision to access*: Stigma related to mental health. CYP believing they would not be taken seriously or would not have a say in their treatment. CYP believing they could self-manage.<br>• *Reaching and entering services:* Caregivers feeling hesitant to persist in asking for support for their child. Unfriendly reception staff. Delayed or rejected referrals to CAMHS or AMH.<br>• *Communication and trust*: A lack of continuity of care and insufficient time in consultations. YP concerns about confidentiality. GPs lacking knowledge in how to manage CYP mental health.
CYP from deprived areas (n=8)	• *Decision to access*: CYP oral health was a lower priority for some caregivers than children's physical health and developmental milestones. Caregivers feeling judged on their parenting or blamed for their child's condition.<br>• *Reaching and entering services*: Caregivers lacking practical resources and non-digital information.<br>• *Communication and trust*: Caregivers feeling a sense of powerless and inferiority in the provider-patient interaction.
Looked after children (n=2)	• *Reaching and entering services*: Strict non-attendance and de-registration policies.<br>• *Communication and trust*: A lack of continuity of care and insufficient time in consultations.
Ethnic minority CYP (n=7)	• *Decision to access*: A lack of familiarity within the community of the syndrome/illness and stigma related to mental health. Perception of surveillance by healthcare systems. Experiences of stigma and discrimination. Lack of health information in other languages.<br>• *Reaching and entering services*: Unfriendly reception staff. Lack of knowledge of the healthcare (and education) system.<br>• *Communication and trust:* Repeated consultations resulting in little improvement or misdiagnosis. Lack of GP knowledge about the experiences and needs of ethnic minority groups. Health visiting teams lacking knowledge of culturally specific oral health guidance and feeding practices.
CYP with SEND (n=5)	• *Decision to access*: Caregivers feeling judged on their parenting or blamed for their child's condition.<br>• *Reaching and entering services*: Lack of knowledge of the healthcare (and education) system. Delayed or rejected referrals to secondary or adult care. Caregivers feeling hesitant to persist in asking for support for their child.<br>• *Communication and trust*: Dentists lacking knowledge in caring for CYP with learning difficulties.
CYP with chronic health problems (n=4)	• *Reaching and entering services*: Delayed or rejected referrals to secondary care. Caregivers feeling hesitant to persist in asking for support for their child.<br>• *Communication and trust:* Repeated consultations resulting in little improvement or misdiagnosis. Lack of GP knowledge about some childhood chronic health problems.

For peer review only - http://bmjopen.bmj.com/site/about/guidelines.xhtml

Discussion

Summary

The review identified high-quality evidence, from multiple studies and informants, that CYP access to primary care was affected by caregivers and YP knowing whether symptoms/conditions could be managed at home or whether healthcare expertise was needed, supporting other studies that show patients must identify themselves as a suitable candidate for healthcare services in order to seek access (77,78). The NICE review of access also highlighted the importance of CYP having information about the healthcare services available to them (19). Levels of patients' health and language literacy, access to legitimate health advice via social networks or culturally-appropriate resources, and patients' expectations affect equitable and appropriate use of primary care (11,79,80). This suggests multi-lingual public health information about childhood symptoms/conditions, when and how to seek help should be available online and in public spaces, and professionals that bridge community and primary care services (for example, third sector health workers, health visitors, school nurses, family hub workers) should support caregivers/YP *into* primary care when they identify healthcare needs and there are known language, cultural or trust-related barriers to accessing services (19,81–83).

Many high-quality studies suggested that CYP access to services could be improved by making them easier to reach and enter, for example, by extending opening hours and co-located services. Signals that healthcare settings were family-friendly, such as having posters/information designed for CYP in reception, appropriate to the needs of different age groups, and having welcoming and friendly reception staff were quick-wins. Flexibility, for example, having the option to call, drop-in, *or* use an online system to make an appointment, could facilitate access for caregivers with different needs and preferences in time, communication and support (84). Wealthier caregivers were able to circumvent blocks to timely secondary care by accessing private health care, but this was not possible for all caregivers, suggesting that waiting lists are likely to disadvantage poorer CYP. This is particularly concerning in dentistry where 27,000 children were on NHS waiting lists for specialist dental care, assessment or procedures in January 2023 (85). Combined with general practice workforce shortages (14), increased CYP morbidities (15), and lower caregiver self-efficacy, health and language literacy in deprived areas, the importance of proactive efforts to address inequalities is evident (84).

Although improving CYP access to mental health care is a high policy priority (8,29), there was strong evidence that YP were reluctant to consult with GPs about mental health concerns without a pre-existing relationship with them. Feelings of fear or embarrassment, experiences of discrimination, and/or negative interactions with HCPs, for example, feeling dismissed or unheard, increased CYP's and caregivers' reticence to disclose concerns, a finding mirrored in the NICE review (19). Prioritising continuity of care for YP to enable trust to develop in a context where GPs are increasingly working part-time and locuming needs consideration (86–88). Caregivers, YP, and HCPs also reported gaps in GPs knowledge/competence in managing CYP mental health, and long-wait times and rejected referrals to secondary care, indicating a need to increase medical training in child and adolescent mental health (89,90). Although school-based interventions may alleviate concerns for some children, evidence from large-scale mixed-method evaluations suggests that CYP with moderately high emotional needs and those with additional needs (for example, neurodiversity, SEND or difficult

For peer review only - http://bmjopen.bmj.com/site/about/guidelines.xhtml

family circumstances) may fall through the gaps (91,92). There are examples of integrated approaches for children with chronic health conditions whereby GPs are supported by specialists which could bridge this gap including in mental health (93,94). The new role of the primary care CYP mental health practitioner and social prescribing link workers may be able to support CYP waiting for CAMHS, though the evidence for this is not yet known (95,96).

The review highlighted aspects of primary healthcare experiences that were well-evidenced, with multiple studies of high or medium quality across different informants' views. These were: experiences of stigma, discrimination, and embarrassment as access barriers; access affected by the supply of services; knowledge, confidence, and information facilitating CYP's/caregivers' access; and HCPs needing to enable CYP/caregivers to disclose their concerns. However, we also identified several evidence gaps where more research was needed: 1) CYP's perspectives on creating family-friendly healthcare settings; 2) CYP's views on the impact of delayed or rejected referrals; 3) high quality studies on managing confidentiality and parental involvement, including caregivers' perspectives; and 4) high quality studies on experiences of access to oral healthcare and optometry.

Strengths and limitations

Our review was rigorously conducted and included quality appraisal. Mapping patterns of facilitators/barriers across different sub-populations with higher health needs revealed that access was affected by caregivers' needing to be able to confidently advocate for their child's needs. It also highlighted the multi-layered barriers that exist for some groups, including ethnic minority CYP, and the lack of current evidence on access for looked after children. It extends the findings of the NICE review by highlighting how local healthcare knowledge within communities and social networks affects CYP's and caregivers' decision to seek help, the impact of delays or rejected referrals to secondary care, and areas where HCPs may lack knowledge and competence.

Regarding limitations, we only double-screened 20% of title/abstracts and we may have missed reports due to the array of terms for primary care, for example, we did not include search terms specific to health visiting, walk-in centres, or sexual health clinics. Our definition of access included being able to use healthcare services and have healthcare needs met. Consequently, we viewed communication problems in consultations as part of the negotiation of access and not being referred to secondary care when CYP/caregivers perceived it necessary as a failure have healthcare needs met. Although including terms specific to patient-doctor communication and referral decision-making would have increased the sensitivity of the review, it would have reduced its specificity and increased the resources needed for screening beyond those that were available. Note, recommendations on communicating with CYP can be found in the NICE guidelines (19). We could not screen studies in systematic reviews or search for grey literature due to time and resources constraints, and we may have missed relevant reports, particularly for marginalised groups (e.g. LGBTQ+ YP, migrants). The impact of workforce barriers to access, e.g. recruiting and retaining GPs, were not identified using our search terms and may require specific terms to be added to future reviews.

Conclusions

The review evidence suggests that four policy priorities to improve equitable CYP access to primary care: 1) encouraging CYP/caregivers into healthcare settings through general practices developing

For peer review only - http://bmjopen.bmj.com/site/about/guidelines.xhtml

and maintaining links with community health workers/services, 2) improving CYP/caregivers' understanding of common childhood conditions by providing public health information on common childhood conditions and illnesses in local languages, 3) developing integrated approaches bringing specialist expertise into primary care, and 4) addressing paediatric training gaps for medical students, particularly in child and adolescent mental health.

Figure 1: PRISMA Flow Diagram

Figure 2: Facilitators and barriers to CYP access to primary care

Ethical Approval Statement

Not applicable

References

1. Levesque JF, Harris MF, Russell G. Patient-centred access to health care: conceptualising access at the interface of health systems and populations. Int J Equity Health. 2013;12(1):18.

2. Gulliford M, Figueroa-Munoz J, Morgan M, Hughes D, Gibson B, Beech R, et al. What does 'access to health care' mean? J Health Serv Res Policy. 2002 Jul 1;7(3):186–8.

3. Harnden A, Mayon-White R, Mant D, Kelly D, Pearson G. Child deaths: confidential enquiry into the role and quality of UK primary care. Br J Gen Pract. 2009 Nov 1;59(568):819–24.

4. Royal College of Nursing. Safeguarding children and young people: roles and competencies for healthcare staff: intercollegiate document [Internet]. London: Royal College of Nursing; 2019 Jan [cited 2023 Jan 13]. Available from: https://www.rcn.org.uk/professional-development/publications/pub-007366

5. Cecil E, Bottle A, Cowling T, Majeed A, Wolfe I, Saxena S. Primary Care Access, Emergency Department Visits, and Unplanned Short Hospitalizations in the UK. Pediatrics. 2016 Feb;137(2):e20151492–e20151492.

6. Gill PJ, Goldacre MJ, Mant D, Heneghan C, Thomson A, Seagroatt V, et al. Increase in emergency admissions to hospital for children aged under 15 in England, 1999-2010: national database analysis. Arch Dis Child. 2013 May 1;98(5):328–34.

7. Ruzangi J, Blair M, Cecil E, Greenfield G, Bottle A, Hargreaves D. Trends in healthcare use in children aged less than 15 years: A population-based cohort study in England from 2007 to 2017. BMJ Open. 2020;10(5):e033761–e033761.

8. NHS. The NHS long term plan [Internet]. 2019 [cited 2021 Oct 1]. Available from: https://www.longtermplan.nhs.uk/

9. Hargreaves DS, Elliott MN, Viner RM, Richmond TK, Schuster MA. Unmet Health Care Need in US Adolescents and Adult Health Outcomes. Pediatrics. 2015 Sep 1;136(3):513–20.

10. Ghafari M, Nadi T, Bahadivand-Chegini S, Doosti-Irani A. Global prevalence of unmet need for mental health care among adolescents: A systematic review and meta-analysis. Arch Psychiatr Nurs. 2022 Feb;36:1–6.

For peer review only - http://bmjopen.bmj.com/site/about/guidelines.xhtml

11. Coughlan C, Ruzangi J, Neale F, Nezafat Maldonado B, Blair M, Bottle A, et al. Social and ethnic group differences in healthcare use by children aged 0-14 years: A population-based cohort study in England from 2007 to 2017. Arch Dis Child. 2022;107(1):32–9.

12. Perry M, McGowan A, Roberts R, Cottrell S. Timeliness and equity of infant pertussis vaccination in wales: Analysis of the three dose primary course. Vaccine. 2020;38(6):1402–7.

13. Bishop C, Small N, Parslow R. Healthcare use for children with complex needs: Using routine health data linked to a multiethnic, ongoing birth cohort. BMJ Open. 2018;8(3):e018419–e018419.

14. Nussbaum C, Massou E, Fisher R, Morciano M, Harmer R, Ford J. Inequalities in the distribution of the general practice workforce in England: a practice-level longitudinal analysis. BJGP Open. 2021 Oct;5(5):BJGPO.2021.0066.

15. Chung SC, Mueller S, Green K, Chang WH, Hargrave D, Lai AG. Multimorbidity patterns and risk of hospitalisation in children: A population cohort study of 3.6 million children in England, with illustrative examples from childhood cancer survivors. Lancet Reg Health - Eur. 2022 Sep;20:100433.

16. Coughlan B, Duschinsky R, O'Connor M, Woolgar M. Identifying and managing care for children with autism spectrum disorders in general practice: A systematic review and narrative synthesis. Health Soc Care Community. 2020 Nov;28(6):1928–41.

17. Smales M, Morris H, Savaglio M, Skouteris H, Green R. 'I'm dealing with all these health issues that could have been addressed when I was younger'. Delivery of health services to Australian young people in out-of-home care: Lived experiences. Health Soc Care Community [Internet]. 2022 Jul [cited 2023 Dec 7];30(4). Available from: https://onlinelibrary.wiley.com/doi/10.1111/hsc.13548

18. van der Boor CF, White R. Barriers to Accessing and Negotiating Mental Health Services in Asylum Seeking and Refugee Populations: The Application of the Candidacy Framework. J Immigr Minor Health. 2020 Feb 1;22(1):156–74.

19. National Institute for Health and Care Excellence. Babies, children and young people's experience of healthcare [Internet]. London: National Institute for Health and Care Excellence (NICE); 2021 [cited 2024 Feb 26]. Available from: https://www.nice.org.uk/guidance/ng204/resources/babies-children-and-young-peoples-experience-of-healthcare-pdf-66143714734789

20. Chirewa B, Wakhisi A. Emergency hormonal contraceptive service provision via community pharmacies in the UK: a systematic review of pharmacists' and young women's views, perspectives and experiences. Perspect Public Health. 2020 Mar;140(2):108–16.

21. Ahmadyar M, Rai T, Daly B, Wong G. Improving access of young adults with experience of homelessness to primary care dental services in the UK: A realist synthesis. Community Dent Oral Epidemiol. 2022 Oct;50(5):437–44.

22. McDonagh LK, Saunders JM, Cassell J, Curtis T, Bastaki H, Hartney T, et al. Application of the COM-B model to barriers and facilitators to chlamydia testing in general practice for young people and primary care practitioners: a systematic review. Implement Sci. 2018 Dec;13(1):130.

For peer review only

For peer review only - http://bmjopen.bmj.com/site/about/guidelines.xhtml

23. Tricco AC, Lillie E, Zarin W, O'Brien KK, Colquhoun H, Levac D, et al. PRISMA extension for scoping reviews (PRISMA-ScR): Checklist and explanation. Ann Intern Med. 2018;169(7):467–73.

24. Pace R, Pluye P, Bartlett G, Macaulay AC, Salsberg J, Jagosh J, et al. Testing the reliability and efficiency of the pilot Mixed Methods Appraisal Tool (MMAT) for systematic mixed studies review. Int J Nurs Stud. 2012 Jan;49(1):47–53.

25. Souto RQ, Khanassov V, Hong QN, Bush PL, Vedel I, Pluye P. Systematic mixed studies reviews: Updating results on the reliability and efficiency of the mixed methods appraisal tool. Int J Nurs Stud. 2015 Jan;52(1):500–1.

26. Gough D. Weight of Evidence: a framework for the appraisal of the quality and relevance of evidence. Res Pap Educ. 2007 Jun;22(2):213–28.

27. Gale NK, Heath G, Cameron E, Rashid S, Redwood S. Using the framework method for the analysis of qualitative data in multi-disciplinary health research. BMC Med Res Methodol. 2013 Dec;13(1):117.

28. Ford JA, Wong G, Jones AP, Steel N. Access to primary care for socioeconomically disadvantaged older people in rural areas: a realist review. BMJ Open. 2016 May 1;6(5):e010652.

29. NHS England. Core20PLUS5 – An approach to reducing health inequalities for children and young people [Internet]. 2022 [cited 2023 Aug 16]. Available from: https://www.england.nhs.uk/about/equality/equality-hub/national-healthcare-inequalities-improvement-programme/core20plus5/core20plus5-cyp/

30. Ahmaro L, Lindsey L, Forrest S, Whittlesea C. Young people's perceptions of accessing a community pharmacy for a chlamydia testing kit: a qualitative study based in North East England. BMJ Open. 2021 Sep;11(9):e052228.

31. Alexakis C, Nash A, Lloyd M, Brooks F, Lindsay JO, Poullis A. Inflammatory bowel disease in young patients: challenges faced by black and minority ethnic communities in the UK. Health Soc Care Community. 2015 Nov;23(6):665–72.

32. Appleton R, Loew J, Mughal F. Young people who have fallen through the mental health transition gap: a qualitative study on primary care support. Br J Gen Pract. 2022 Jun;72(719):e413–20.

33. Bosley H, Appleton JV, Henshall C, Jackson D. The influence of perceived accessibility and expertise of healthcare professionals, and service austerity, on mothers' decision-making. Health Soc Care Community. 2021 Mar;29(2):526–34.

34. Brigham L, Maxwell C, Smith A. Leading in practice: a case study of how health visitors share and develop good practice. Community Pract J Community Pract Health Visit Assoc. 2012 May;85(5):24–8.

35. Coleman-Fountain E, Buckley C, Beresford B. Improving mental health in autistic young adults: a qualitative study exploring help-seeking barriers in UK primary care. Br J Gen Pract. 2020 May;70(694):e356–63.

36. Condon L, McClean S, McRae L. 'Differences between the earth and the sky': migrant parents' experiences of child health services for pre-school children in the UK. Prim Health Care Res Dev. 2020;21:e29.

For peer review only

For peer review only - http://bmjopen.bmj.com/site/about/guidelines.xhtml

37. Corry DAS, Leavey G. Adolescent trust and primary care: Help-seeking for emotional and psychological difficulties. J Adolesc. 2017 Jan;54(1):1–8.

38. Coyle CF, Humphris GM, Freeman R. Dentists' training and willingness to treat adolescents with learning disabilities: the mediating role of social and clinical factors. Community Dent Health. 2013 Dec;30(4):263–8.

39. Crocker JC, Evans MR, Powell CVE, Hood K, Butler CC. Why some children hospitalized for pneumonia do not consult with a general practitioner before the day of hospitalization. Eur J Gen Pract. 2013 Dec;19(4):213–20.

40. Crouch L, Reardon T, Farrington A, Glover F, Creswell C. "Just keep pushing": Parents' experiences of accessing child and adolescent mental health services for child anxiety problems. Child Care Health Dev. 2019 May 27;cch.12672.

41. Dando CJ, Brierley R, Saunders K, Mackenzie JM. Health inequalities and health equity challenges for victims of modern slavery. J Public Health. 2019 Dec 20;41(4):681–8.

42. Davey A, Asprey A, Carter M, Campbell JL. Trust, negotiation, and communication: young adults' experiences of primary care services. BMC Fam Pract. 2013 Dec;14(1):202.

43. Dickson CM. Every child has the right to smile!--A qualitative study exploring barriers to dental registration in a SureStart area in Northern Ireland. Community Pract J Community Pract Health Visit Assoc. 2015 Aug;88(8):36–9, 41.

44. Diwakar L, Cummins C, Hackett S, Rees M, Charles L, Kerrigan C, et al. Parent experiences with paediatric allergy pathways in the West Midlands: A qualitative study. Clin Exp Allergy. 2019 Mar;49(3):357–65.

45. Eskytė I, Gray-Burrows KA, Owen J, Sykes-Muskett B, Pavitt SH, West R, et al. Organizational Barriers to Oral Health Conversations Between Health Visitors and Parents of Children Aged 9–12 Months Old. Front Public Health. 2021 Feb 23;9:578168.

46. Fox F, Aabe N, Turner K, Redwood S, Rai D. "It was like walking without knowing where I was going": A Qualitative Study of Autism in a UK Somali Migrant Community. J Autism Dev Disord. 2017 Feb;47(2):305–15.

47. Fox F, Stallard P, Cooney G. GPs role identifying young people who self-harm: a mixed methods study. Fam Pract. 2015 May 8;cmv031.

48. French B, Perez Vallejos E, Sayal K, Daley D. Awareness of ADHD in primary care: stakeholder perspectives. BMC Fam Pract. 2020 Dec;21(1):45.

49. Henderson E, Rubin G. A model of roles and responsibilities in oral health promotion based on perspectives of a community-based initiative for pre-school children in the UK. Br Dent J. 2014 Mar;216(5):E11–E11.

50. Ingram J, Cabral C, Hay AD, Lucas PJ, Horwood J. Parents' information needs, self-efficacy and influences on consulting for childhood respiratory tract infections: a qualitative study. BMC Fam Pract. 2013 Dec;14(1):106.

51. Jobanputra S, Singh S. What are general practitioners' views on the management of adolescents with mental health disorders? A qualitative study. Educ Prim Care. 2020 Sep 2;31(5):323–4.

For peer review only

For peer review only - http://bmjopen.bmj.com/site/about/guidelines.xhtml

52. Jones LF, Ricketts E, Town K, Rugman C, Lecky D, Folkard K, et al. Chlamydia and HIV testing, contraception advice, and free condoms offered in general practice: a qualitative interview study of young adults' perceptions of this initiative. Br J Gen Pract. 2017 Jul;67(660):e490–500.

53. Lewney J, Holmes RD, Rankin J, Exley C. Health visitors' views on promoting oral health and supporting clients with dental health problems: a qualitative study. J Public Health. 2019 Mar 1;41(1):e103–8.

54. McDonagh LK, Harwood H, Saunders JM, Cassell JA, Rait G. How to increase chlamydia testing in primary care: a qualitative exploration with young people and application of a meta-theoretical model. Sex Transm Infect. 2020 Dec;96(8):571–81.

55. Mughal F, Dikomitis L, Babatunde OO, Chew-Graham CA. Experiences of general practice care for self-harm: a qualitative study of young people's perspectives. Br J Gen Pract. 2021 Oct 1;71(711):e744–52.

56. Muirhead V, Subramanian SK, Wright D, Wong FSL. How do foster carers manage the oral health of children in foster care? A qualitative study. Community Dent Oral Epidemiol. 2017 Dec;45(6):529–37.

57. Neill SJ, Jones CH, Lakhanpaul M, Roland DT, Thompson MJ. Parents' help-seeking behaviours during acute childhood illness at home: A contribution to explanatory theory. J Child Health Care. 2016 Mar;20(1):77–86.

58. Neill SJ, Jones CHD, Lakhanpaul M, Roland DT, Thompson MJ, the ASK SNIFF research team. Parent's information seeking in acute childhood illness: what helps and what hinders decision making? Health Expect. 2015 Dec;18(6):3044–56.

59. O'Brien D, Harvey K, Creswell C. Barriers to and facilitators of the identification, management and referral of childhood anxiety disorders in primary care: a survey of general practitioners in England. BMJ Open. 2019 Apr;9(4):e023876.

60. O'Brien D, Harvey K, Young B, Reardon T, Creswell C. GPs' experiences of children with anxiety disorders in primary care: a qualitative study. Br J Gen Pract. 2017 Dec;67(665):e888–98.

61. Ochieng BMN. Healthy weight maintenance strategy in early childhood: The views of black African migrant parents and health visitors. Health Soc Care Community. 2020 Sep;28(5):1551–9.

62. Rapley T, May C, Smith N, Foster HE. 'Snakes & Ladders': factors influencing access to appropriate care for children and young people with suspected juvenile idiopathic arthritis – a qualitative study. Pediatr Rheumatol. 2021 Dec;19(1):43.

63. Rashed AN, Mohamud N, Lam A, Hamadallah H, Terry D, Tomlin S. Community pharmacy role in children's health in England: Experiences and opinions of parents and young people. Health Soc Care Community. 2022 Nov;30(6):2362–71.

64. Redsell SA, Swift JA, Nathan D, Siriwardena AN, Atkinson P, Glazebrook C. UK health visitors' role in identifying and intervening with infants at risk of developing obesity: Health visitors' and infant obesity risk. Matern Child Nutr. 2013 Jul;9(3):396–408.

For peer review only - http://bmjopen.bmj.com/site/about/guidelines.xhtml

65. Rickett B, Johnson K, Ingle H, Reynolds M. Support for parents/carers of primary school aged gender diverse children in England, UK: a mixed-method analysis of experiences with health services. Health Sociol Rev. 2021 Jan 2;30(1):9–24.

66. Roberts J, Crosland A, Fulton J. Patterns of engagement between GPs and adolescents presenting with psychological difficulties: a qualitative study. Br J Gen Pract. 2014 May;64(622):e246–54.

67. Roberts K, Condon L. How do parents look after children's teeth? A qualitative study of attitudes to oral health in the early years. Community Pract J Community Pract Health Visit Assoc. 2014 Apr;87(4):32–5.

68. Salaheddin K, Mason B. Identifying barriers to mental health help-seeking among young adults in the UK: a cross-sectional survey. Br J Gen Pract. 2016 Oct;66(651):e686–92.

69. Satherley RM, Wolfe I, Lingam R. Experiences of healthcare for mothers of children with ongoing illness, living in deprived neighbourhoods health and place. Health Place. 2021 Sep;71:102661.

70. Turnbull G, Scott RH, Mann S, Wellings K. Accessing emergency contraception pills from pharmacies: the experience of young women in London. BMJ Sex Reprod Health. 2021 Jan;47(1):27–31.

71. Turner KM, Salisbury C, Shield JPH. Parents' views and experiences of childhood obesity management in primary care: a qualitative study. Fam Pract. 2012 Aug 1;29(4):476–81.

72. Usher-Smith JA, Thompson MJ, Zhu H, Sharp SJ, Walter FM. The pathway to diagnosis of type 1 diabetes in children: a questionnaire study. BMJ Open. 2015 Mar 17;5(3):e006470–e006470.

73. Williams A, Mackintosh J, Bateman B, Holland S, Rushworth A, Brooks A, et al. The development of a designated dental pathway for looked after children. Br Dent J. 2014 Feb;216(3):E6–E6.

74. Williams R, Hewison A, Stewart M, Liles C, Wildman S. 'We are doing our best': African and African-Caribbean fatherhood, health and preventive primary care services, in England: African and African-Caribbean fatherhood and preventive primary care services. Health Soc Care Community. 2012 Mar;20(2):216–23.

75. Wilson S, Ctori I, Suttle C, Conway M, Shah R. How accessible is primary eye care for children in England? Ophthalmic Physiol Opt. 2021 Sep;41(5):1021–33.

76. Yassaee AA, Hargreaves DS, Chester K, Lamb S, Hagell A, Brooks FM. Experience of Primary Care Services Among Early Adolescents in England and Association With Health Outcomes. J Adolesc Health. 2017 Apr 1;60(4):388–94.

77. Dixon-Woods M, Cavers D, Agarwal S, Annandale E, Arthur A, Harvey J, et al. Conducting a critical interpretive synthesis of the literature on access to healthcare by vulnerable groups. BMC Med Res Methodol. 2006 Jul 26;6(1):35.

78. Hudson N, Culley L, Johnson M, McFeeters M, Robertson N, Angell E, et al. Asthma management in British South Asian children: an application of the candidacy framework to a qualitative understanding of barriers to effective and accessible asthma care. BMC Public Health. 2016 Dec;16(1):510.

For peer review only - http://bmjopen.bmj.com/site/about/guidelines.xhtml

79. Viner RM, Blackburn F, White F, Mannie R, Parr T, Nelson S, et al. The impact of out-of-hospital models of care on paediatric emergency department presentations. Arch Dis Child. 2018 Feb;103(2):128–36.

80. Dale J, Potter R, Owen K, Parsons N, Realpe A, Leach J. Retaining the general practitioner workforce in England: what matters to GPs? A cross-sectional study. BMC Fam Pract. 2015 Dec;16(1):140.

81. Vanden Bossche D, Willems S, Decat P. Understanding Trustful Relationships between Community Health Workers and Vulnerable Citizens during the COVID-19 Pandemic: A Realist Evaluation. Int J Environ Res Public Health. 2022 Feb 22;19(5):2496.

82. Canvin K, Jones C, Marttila A, Burstrom B, Whitehead M. Can I risk using public services? Perceived consequences of seeking help and health care among households living in poverty: qualitative study. J Epidemiol Community Health. 2007 Nov 1;61(11):984–9.

83. Thomas L, Parker S, Song H, Gunatillaka N, Russell G, Harris M. Health service brokerage to improve primary care access for populations experiencing vulnerability or disadvantage: a systematic review and realist synthesis. BMC Health Serv Res. 2019 Dec;19(1):269.

84. Gkiouleka A, Wong G, Sowden S, Bambra C, Siersbaek R, Manji S, et al. Reducing health inequalities through general practice. Lancet Public Health. 2023 Jun;8(6):e463–72.

85. Liberal Democrats. 27,000 children on waiting lists for specialised dental care [Internet]. 2023 [cited 2023 Sep 5]. Available from: https://www.libdems.org.uk/press/release/27000-children-on-waiting-lists-for-specialised-dental-care

86. Jefferson L, Holmes M. GP workforce crisis: what can we do now? Br J Gen Pract. 2022 May;72(718):206–7.

87. Simon C, Forde E, Fraser A, Wedderburn C, Aylwin S. What is the root cause of the GP workforce crisis? Br J Gen Pract. 2018 Dec;68(677):589–90.

88. Navein A, Gonzalez-Viana E, Mehmeti A, Hargreaves D, Elvins R, Churchill R. Barriers and facilitators to continuity and co-ordination of healthcare for under 18 years old: a systematic review. Arch Dis Child. 2023 Oct;108(10):783–8.

89. England E, Nash V, Hawthorne K. GP training in mental health needs urgent reform. BMJ. 2017 Mar 16;j1311.

90. Salmon G, Tombs M. Teaching medical students child and adolescent psychiatry (CAP). J Ment Health Train Educ Pract. 2019 Aug 29;14(5):348–60.

91. Ellins J, Hocking L, Al-Haboubi M, Newbould J, Fenton SJ, Daniel K, et al. Early evaluation of the Children and Young People's Mental Health Trailblazer programme: a rapid mixed-methods study. Health Soc Care Deliv Res. 2023 Jun;1–137.

92. Wolpert M, Humphrey N, Belsky J, Deighton J. Embedding mental health support in schools: learning from the Targeted Mental Health in Schools (TaMHS) national evaluation. Emot Behav Difficulties. 2013 Sep;18(3):270–83.

93. Woodman J, Lewis H, Cheung R, Gilbert R, Wijlaars LP. Integrating primary and secondary care for children and young people: sharing practice. Arch Dis Child. 2016 Sep;101(9):792–7.

For peer review only - http://bmjopen.bmj.com/site/about/guidelines.xhtml

94. Patel S, Hodgkinson T, Fowler R, Pryde K, Ward R. Integrating acute services for children and young people across primary and secondary care. Br J Gen Pract. 2020 Apr;70(693):158–9.

95. NHS England. Expanding our workforce [Internet]. n.d. [cited 2024 Feb 23]. Available from: https://www.england.nhs.uk/gp/expanding-our-workforce/

96. Fancourt D, Burton A, Bu F, Deighton J, Turner R, Wright J, et al. Wellbeing while waiting evaluating social prescribing in CAMHS: study protocol for a hybrid type II implementation-effectiveness study. BMC Psychiatry. 2023 May 10;23(1):328.

Acknowledgements

We would like to thank German Alarcon Garavito, Macarena Chepo, Sophie Moniz, Federico Redin, and Cecilia Vindrola-Padros from the Rapid Research Evaluation and Appraisal (RREAL) Lab for their contributions to the screening for this review.

Contributors

LH, CP, JW, KH and SM contributed to the study's conception, LH conducted the searches, and LH and EA completed the screening. LH, EA and CP conducted the data extraction and quality appraisal, and LH and EA carried out the data synthesis. LH led and EA contributed to drafting the manuscripts and all authors provided critical revisions and editing. All authors reviewed the manuscript.

Funding

This study was funded by the National Institute for Health and Care Research (NIHR) through the Children and Families Policy Research Unit (PR-PRU-1217-21301). The views expressed are those of the authors and not necessarily those of the NIHR or the Department of Health and Social Care.

Competing interests

The authors declare no conflicts of interest.

Data availability statement

All data relevant to the study are included in the article or uploaded as online supplemental information.

For peer review only

For peer review only - http://bmjopen.bmj.com/site/about/guidelines.xhtml

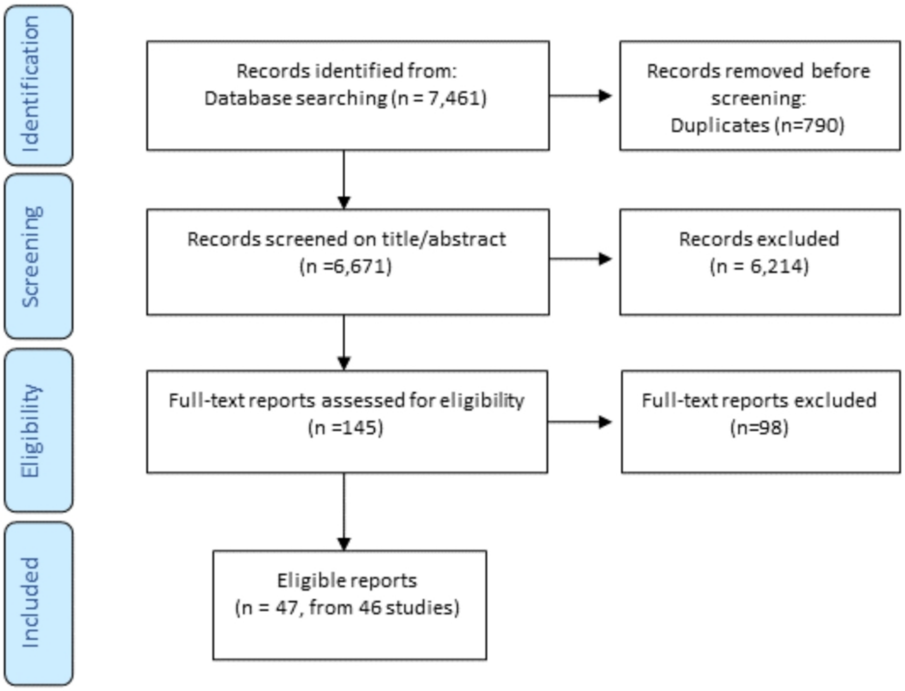

PRISMA flow diagram

254x195mm (300 x 300 DPI)

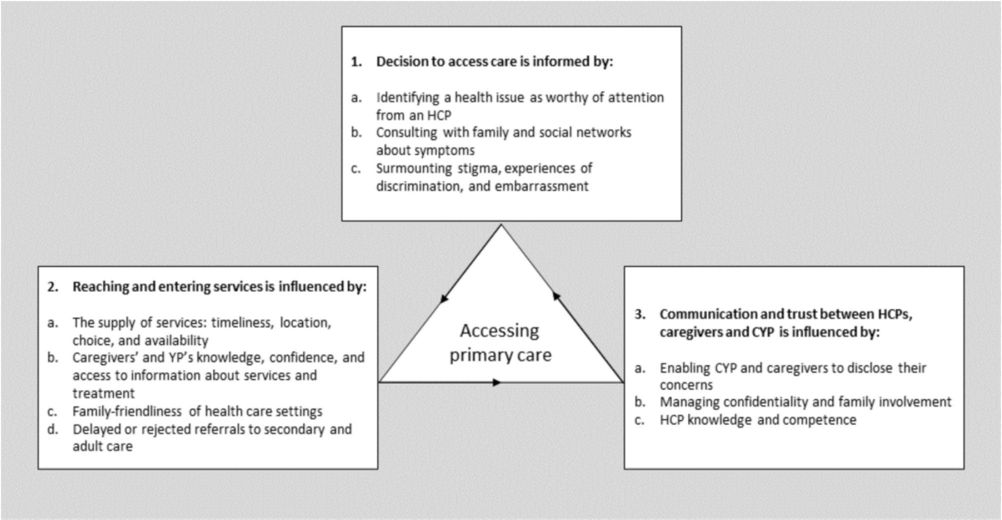

Facilitators and barriers to CYP access to primary care

254x131mm (300 x 300 DPI)

Preferred Reporting Items for Systematic reviews and Meta-Analyses extension for Scoping Reviews (PRISMA-ScR) Checklist

SECTION	ITEM	PRISMA-ScR CHECKLIST ITEM	REPORTED ON PAGE #
**TITLE**			
Title	1	Identify the report as a scoping review.	
**ABSTRACT**			
Structured summary	2	Provide a structured summary that includes (as applicable): background, objectives, eligibility criteria, sources of evidence, charting methods, results, and conclusions that relate to the review questions and objectives.	
**INTRODUCTION**			
Rationale	3	Describe the rationale for the review in the context of what is already known. Explain why the review questions/objectives lend themselves to a scoping review approach.	
Objectives	4	Provide an explicit statement of the questions and objectives being addressed with reference to their key elements (e.g., population or participants, concepts, and context) or other relevant key elements used to conceptualize the review questions and/or objectives.	
**METHODS**			
Protocol and registration	5	Indicate whether a review protocol exists; state if and where it can be accessed (e.g., a Web address); and if available, provide registration information, including the registration number.	
Eligibility criteria	6	Specify characteristics of the sources of evidence used as eligibility criteria (e.g., years considered, language, and publication status), and provide a rationale.	
Information sources*	7	Describe all information sources in the search (e.g., databases with dates of coverage and contact with authors to identify additional sources), as well as the date the most recent search was executed.	
Search	8	Present the full electronic search strategy for at least 1 database, including any limits used, such that it could be repeated.	
Selection of sources of evidence†	9	State the process for selecting sources of evidence (i.e., screening and eligibility) included in the scoping review.	
Data charting process‡	10	Describe the methods of charting data from the included sources of evidence (e.g., calibrated forms or forms that have been tested by the team before their use, and whether data charting was done independently or in duplicate) and any processes for obtaining and confirming data from investigators.	
Data items	11	List and define all variables for which data were sought and any assumptions and simplifications made.	
Critical appraisal of individual sources of evidence§	12	If done, provide a rationale for conducting a critical appraisal of included sources of evidence; describe the methods used and how this information was used in any data synthesis (if appropriate).	
Synthesis of results	13	Describe the methods of handling and summarizing the data that were charted.	

For peer review only

SECTION	ITEM	PRISMA-ScR CHECKLIST ITEM	REPORTED ON PAGE #
**RESULTS**			
Selection of sources of evidence	14	Give numbers of sources of evidence screened, assessed for eligibility, and included in the review, with reasons for exclusions at each stage, ideally using a flow diagram.	
Characteristics of sources of evidence	15	For each source of evidence, present characteristics for which data were charted and provide the citations.	
Critical appraisal within sources of evidence	16	If done, present data on critical appraisal of included sources of evidence (see item 12).	
Results of individual sources of evidence	17	For each included source of evidence, present the relevant data that were charted that relate to the review questions and objectives.	
Synthesis of results	18	Summarize and/or present the charting results as they relate to the review questions and objectives.	
**DISCUSSION**			
Summary of evidence	19	Summarize the main results (including an overview of concepts, themes, and types of evidence available), link to the review questions and objectives, and consider the relevance to key groups.	
Limitations	20	Discuss the limitations of the scoping review process.	
Conclusions	21	Provide a general interpretation of the results with respect to the review questions and objectives, as well as potential implications and/or next steps.	
**FUNDING**			
Funding	22	Describe sources of funding for the included sources of evidence, as well as sources of funding for the scoping review. Describe the role of the funders of the scoping review.	

JBI = Joanna Briggs Institute; PRISMA-ScR = Preferred Reporting Items for Systematic reviews and Meta-Analyses extension for Scoping Reviews.

* Where *sources of evidence* (see second footnote) are compiled from, such as bibliographic databases, social media platforms, and Web sites.

† A more inclusive/heterogeneous term used to account for the different types of evidence or data sources (e.g., quantitative and/or qualitative research, expert opinion, and policy documents) that may be eligible in a scoping review as opposed to only studies. This is not to be confused with *information sources* (see first footnote).

‡ The frameworks by Arksey and O'Malley (6) and Levac and colleagues (7) and the JBI guidance (4, 5) refer to the process of data extraction in a scoping review as data charting.

§ The process of systematically examining research evidence to assess its validity, results, and relevance before using it to inform a decision. This term is used for items 12 and 19 instead of "risk of bias" (which is more applicable to systematic reviews of interventions) to include and acknowledge the various sources of evidence that may be used in a scoping review (e.g., quantitative and/or qualitative research, expert opinion, and policy document).

From: Tricco AC, Lillie E, Zarin W, O'Brien KK, Colquhoun H, Levac D, et al. PRISMA Extension for Scoping Reviews (PRISMAScR): Checklist and Explanation. Ann Intern Med. 2018;169:467–473. doi: 10.7326/M18-0850.

For peer review only

Additional file 2 – Search terms

Cinahl Plus search

(MH “Health Services Accessibility”) or (MH “Quality of Health Care”) or (MH “Help Seeking Behavior”) or (TI(“access to health service*” or “access to care” or “access to health care” or “health equity” or inequal* or equality or disparit* or unequal or gap* or gradient* or disadvantage*)) or (AB(“access to health service*” or “access to care” or “access to health care” or “health equity” or inequal* or equality or disparit* or unequal or gap* or gradient* or disadvantage*)) AND (MH “Primary Health Care”) or (MH “Primary Nursing”) or (MH “Physicians, Family”) or (MH “Family Practice”) or (TI(“primary health care” or “primary care” or “general practice*”) or “GP surger*” or “dentist*” or “general practitioner*” or “community pharmac*”) or (AB(“primary health care” or “primary care” or “general practice*” or “community pharmac*”) AND (MH Child) or (MH Adolescence) or (MH Infant) or (MH “Parent-Child Relations”) or (TI(child* or adolescen* or infant* or “young people” or youth or juvenile* or teenager* or student* or pupil* or “young adult*” or preschool*)) or (AB(child* or adolescen* or infant* or “young people” or youth or juvenile* or teenager* or student* or pupil* or “young adult*” or preschool*)) AND (MH "United Kingdom") or (MH England) or (MH Wales) or (MH Scotland) or (MH “Northern Ireland”) or (MH “Great Britain”) or (TX(UK or “United Kingdom" or England or Wales or Scotland or “Northern Ireland” or “N. Ireland” or “Great Britain”) AND (MH “Patient Satisfaction”) or (MH “Patient Preference”) or (MH “Health Knowledge”) or (TI(perceived or experience* or “attitude* to health” or facilitator* or enabler* or barrier* or promot* or inhibit* or view* or perspective*) or (AB(perceived or experience* or “attitude* to health” or facilitator* or enabler* or barrier* or promot* or inhibit* or view* or perspective*)

Filter: 2012-2022

Psycinfo search

((Health Care Access).sh. or (Health Care Utilization).sh. or (Health Disparities).sh. or (Help Seeking Behavior).sh. or (Health Care Seeking Behavior).sh. or (Quality of Care).sh. or (access to health service*).ti,ab. or (access to care).ti,ab. or (access to health care).ti,ab. or (health equity).ti,ab. or (inequal*).ti,ab. or (equality).ti,ab. or (disparit*).ti,ab. or (unequal).ti,ab. or (gap*).ti,ab. or (gradient*).ti,ab. or (disadvantage*).ti,ab.) AND ((Primary Health Care).sh. or (General Practitioners).sh. or (Family Physicians).sh. or (Pharmacy).sh. or (Dentists).sh. or (primary health care).ti,ab. or (primary care).ti,ab. or (general practice*).ti,ab. or (GP surger*).ti,ab. or (dentist*).ti,ab. or (general practitioner*).ti,ab. or (community pharmac*).ti,ab.) AND ((Child Behavior).sh. or (Early Adolescence).sh. or (Adolescent Psychology).sh. or (Parent-Child Relations).sh. or (child*).ti,ab. or (adolescen*).ti,ab. or (infant*).ti,ab. or (young people).ti,ab. or (youth).ti,ab. or (juvenile*).ti,ab. or (teenager*).ti,ab. or (student*).ti,ab. or (pupil*).ti,ab. or (young adult*) or (preschool).ti,ab.) AND ((United Kingdom).af. or (England).af. or (Wales).af. or (Scotland).af. or (Northern Ireland).af. or (Great Britain).af.) AND ((Client Satisfaction).sh. or (Client Attitudes).sh. or (Health Knowledge).sh. or (Treatment Barriers).sh. or (perceived).ti,ab. or (experience*).ti,ab. or (attitude* to health).ti,ab. or (facilitator*).ti,ab. or (enabler*).ti,ab. or (barrier*).ti,ab. or (promot*).ti,ab. or (inhibit*).ti,ab. or (view*).ti,ab. or (perspective*).ti,ab.)

Filter: 2012-2022

Web of Science Core Collection Social Sciences Citation Index (SSCI)

(TS=("health care access" OR "help seeking behavior" OR "help seeking behaviour" OR "quality of care" OR "access to health service*" OR "access to care" OR "access to health care" OR "health equity" or inequal* or equality OR disparit* OR unequal OR gap* OR gradient* OR disadvantage*)) AND (TS=("primary health care" OR "general practitioner*" OR "family physician*" OR "primary care" OR "general practice*" OR "GP surger*" OR dentist* OR "dental care" OR "community pharmac*")) AND (TS=("child behavior" OR "early adolescence" OR "adolescent psychology" OR "parent-child relation*" OR child* OR adolescen* OR infant* OR "young people" OR youth OR juvenile* OR teenage* OR student* OR pupil* OR "young adult*" OR preschool)) AND (ALL=("United Kingdom" OR England OR Wales OR Scotland OR "Northern Ireland" OR "Great Britain")) AND (TS=("patient satisfaction" OR "patient preference*" OR "health knowledge" OR perceived OR experience* OR "attitude* to health" OR facilitator* OR enabler* OR barrier* OR promot* OR inhib* OR view* OR perspective*))

Filter: 2012-2022

SCOPUS

ALL((Health Services Accessibility OR "access to health services" OR "access to care" OR Health Equity OR "health equity" OR inequality OR inequalities OR equality OR disparity OR disparities OR unequal OR gap OR gaps OR gradients OR disadvantage OR health service utilisation OR health service utilisation OR "health resource utilisation" OR "health resource utilisation" OR health care seeking behaviour OR health care seeking behavior OR Health Care Quality OR Health Care Evaluation) AND (Primary Health Care OR "primary health care" OR "primary care" OR Primary Care Nursing OR Physicians Primary Care OR General Practice OR "general practice*" OR "GP surger*" OR General Practice Dental OR "dentist*" OR General Practitioners OR "general practitioner*" OR Community Pharmacy Services OR "community pharmac*" or "health visitor" OR pediatric care OR paediatric care) AND (Child or child* or Adolescent or adolescen* or Infant or infant* or "young people" or youth or juvenile* or teenager* or "young adult*" OR child* pre-school OR child* health) AND ("United Kingdom" OR England OR Wales OR Scotland OR "Northern Ireland" OR "N. Ireland") AND (Perception* OR perceived OR experience* OR Patient Satisfaction OR Patient Preference OR Attitude to Health OR Facilitator* OR enabler* OR barrier* OR Patient Acceptance of Health Care)) AND PUBYEAR > 2011 AND PUBYEAR < 2023 AND (LIMIT-TO (LANGUAGE,"English"))

Pubmed

Search: (("health services accessibility"[MeSH Terms] OR ("health"[All Fields] AND "services"[All Fields] AND "accessibility"[All Fields]) OR "health services accessibility"[All Fields] OR "access to health services"[All Fields] OR "access to care"[All Fields] OR ("health equity"[MeSH Terms] OR ("health"[All Fields] AND "equity"[All Fields]) OR "health equity"[All Fields]) OR "health equity"[All Fields] OR ("inequalities"[All Fields] OR "inequality"[All Fields] OR "inequities"[All Fields] OR "inequity"[All Fields]) OR ("inequalities"[All Fields] OR "inequality"[All Fields] OR "inequities"[All Fields] OR "inequity"[All Fields]) OR ("equal"[All Fields] OR "equaled"[All Fields] OR "equaling"[All Fields] OR "equalisation"[All Fields] OR "equalise"[All Fields] OR "equalised"[All Fields] OR "equalises"[All Fields] OR "equalising"[All Fields] OR "equalities"[All Fields] OR "equality"[All Fields] OR "equalization"[All Fields] OR "equalizations"[All Fields] OR "equalize"[All Fields] OR "equalized"[All Fields] OR "equalizer"[All Fields] OR "equalizers"[All Fields] OR "equalizes"[All Fields] OR "equalizing"[All Fields] OR "equalled"[All Fields] OR "equalling"[All Fields] OR "equally"[All Fields] OR "equals"[All Fields]) OR ("disparate"[All Fields] OR "disparately"[All Fields] OR "disparities"[All Fields] OR "disparity"[All Fields]) OR

For peer review only

("disparate"[All Fields] OR "disparately"[All Fields] OR "disparities"[All Fields] OR "disparity"[All Fields]) OR ("unequal"[All Fields] OR "unequally"[All Fields] OR "unequals"[All Fields]) OR "gap"[All Fields] OR "gaps"[All Fields] OR ("gradient"[All Fields] OR "gradient s"[All Fields] OR "gradients"[All Fields]) OR ("disadvantage"[All Fields] OR "disadvantageous"[All Fields] OR "disadvantageously"[All Fields] OR "disadvantages"[All Fields] OR "disadvantaging"[All Fields] OR "vulnerable populations"[MeSH Terms] OR ("vulnerable"[All Fields] AND "populations"[All Fields]) OR "vulnerable populations"[All Fields] OR "disadvantaged"[All Fields]) OR (("health services"[MeSH Terms] OR ("health"[All Fields] AND "services"[All Fields]) OR "health services"[All Fields] OR ("health"[All Fields] AND "service"[All Fields]) OR "health service"[All Fields]) AND ("statistics and numerical data"[MeSH Subheading] OR ("statistics"[All Fields] AND "numerical"[All Fields] AND "data"[All Fields]) OR "statistics and numerical data"[All Fields] OR "utilization"[All Fields] OR "utilisation"[All Fields] OR "utilisations"[All Fields] OR "utilise"[All Fields] OR "utilised"[All Fields] OR "utilises"[All Fields] OR "utilising"[All Fields] OR "utilities"[All Fields] OR "utility"[All Fields] OR "utilizations"[All Fields] OR "utilize"[All Fields] OR "utilized"[All Fields] OR "utilizer"[All Fields] OR "utilizers"[All Fields] OR "utilizes"[All Fields] OR "utilizing"[All Fields])) OR (("health services"[MeSH Terms] OR ("health"[All Fields] AND "services"[All Fields]) OR "health services"[All Fields] OR ("health"[All Fields] AND "service"[All Fields]) OR "health service"[All Fields]) AND ("statistics and numerical data"[MeSH Subheading] OR ("statistics"[All Fields] AND "numerical"[All Fields] AND "data"[All Fields]) OR "statistics and numerical data"[All Fields] OR "utilization"[All Fields] OR "utilisation"[All Fields] OR "utilisations"[All Fields] OR "utilise"[All Fields] OR "utilised"[All Fields] OR "utilises"[All Fields] OR "utilising"[All Fields] OR "utilities"[All Fields] OR "utility"[All Fields] OR "utilizations"[All Fields] OR "utilize"[All Fields] OR "utilized"[All Fields] OR "utilizer"[All Fields] OR "utilizers"[All Fields] OR "utilizes"[All Fields] OR "utilizing"[All Fields])) OR "health resource utilisation"[All Fields] OR "health resource utilisation"[All Fields] OR ("health care seeking behaviour"[All Fields] OR "patient acceptance of health care"[MeSH Terms] OR ("patient"[All Fields] AND "acceptance"[All Fields] AND "health"[All Fields] AND "care"[All Fields]) OR "patient acceptance of health care"[All Fields] OR ("health"[All Fields] AND "care"[All Fields] AND "seeking"[All Fields] AND "behavior"[All Fields]) OR "health care seeking behavior"[All Fields]) OR ("health care seeking behaviour"[All Fields] OR "patient acceptance of health care"[MeSH Terms] OR ("patient"[All Fields] AND "acceptance"[All Fields] AND "health"[All Fields] AND "care"[All Fields]) OR "patient acceptance of health care"[All Fields] OR ("health"[All Fields] AND "care"[All Fields] AND "seeking"[All Fields] AND "behavior"[All Fields]) OR "health care seeking behavior"[All Fields]) OR ("quality of health care"[MeSH Terms] OR ("quality"[All Fields] AND "health"[All Fields] AND "care"[All Fields]) OR "quality of health care"[All Fields] OR ("health"[All Fields] AND "care"[All Fields] AND "quality"[All Fields]) OR "health care quality"[All Fields]) OR (("delivery of health care"[MeSH Terms] OR ("delivery"[All Fields] AND "health"[All Fields] AND "care"[All Fields]) OR "delivery of health care"[All Fields] OR ("health"[All Fields] AND "care"[All Fields]) OR "health care"[All Fields]) AND ("evaluability"[All Fields] OR "evaluate"[All Fields] OR "evaluated"[All Fields] OR "evaluates"[All Fields] OR "evaluating"[All Fields] OR "evaluation"[All Fields] OR "evaluation s"[All Fields] OR "evaluations"[All Fields] OR "evaluative"[All Fields] OR "evaluatively"[All Fields] OR "evaluatives"[All Fields] OR "evaluator"[All Fields] OR "evaluator s"[All Fields] OR "evaluators"[All Fields]))) AND ("primary health care"[MeSH Terms] OR ("primary"[All Fields] AND "health"[All Fields] AND "care"[All Fields]) OR "primary health care"[All Fields] OR "primary health care"[All Fields] OR "primary care"[All Fields] OR ("primary nursing"[MeSH Terms] OR ("primary"[All Fields] AND "nursing"[All Fields]) OR "primary nursing"[All Fields] OR ("primary"[All Fields] AND "care"[All Fields] AND "nursing"[All Fields]) OR "primary care nursing"[All Fields] OR "primary care nursing"[MeSH Terms] OR ("primary"[All Fields] AND "care"[All Fields] AND "nursing"[All Fields])) OR ("physicians, primary care"[MeSH Terms] OR ("physicians"[All Fields] AND

"primary"[All Fields] AND "care"[All Fields]) OR "primary care physicians"[All Fields] OR ("physicians"[All Fields] AND "primary"[All Fields] AND "care"[All Fields]) OR "physicians primary care"[All Fields]) OR ("general practice"[MeSH Terms] OR ("general"[All Fields] AND "practice"[All Fields]) OR "general practice"[All Fields]) OR "general practice"[All Fields] OR "gp surger*"[All Fields] OR ("general practice, dental"[MeSH Terms] OR ("general"[All Fields] AND "practice"[All Fields] AND "dental"[All Fields]) OR "dental general practice"[All Fields] OR ("general"[All Fields] AND "practice"[All Fields] AND "dental"[All Fields]) OR "general practice dental"[All Fields]) OR "dentist*"[All Fields] OR ("general practitioners"[MeSH Terms] OR ("general"[All Fields] AND "practitioners"[All Fields]) OR "general practitioners"[All Fields]) OR "general practitioner*"[All Fields] OR ("community pharmacy services"[MeSH Terms] OR ("community"[All Fields] AND "pharmacy"[All Fields] AND "services"[All Fields]) OR "community pharmacy services"[All Fields]) OR "community pharmac*"[All Fields] OR "health visitor"[All Fields] OR ("pediatr care wilmington"[Journal] OR ("pediatric"[All Fields] AND "care"[All Fields]) OR "pediatric care"[All Fields]) OR (("paediatrics"[All Fields] OR "pediatrics"[MeSH Terms] OR "pediatrics"[All Fields] OR "paediatric"[All Fields] OR "pediatric"[All Fields]) AND "care"[All Fields])) AND ("child"[MeSH Terms] OR "child"[All Fields] OR "children"[All Fields] OR "child s"[All Fields] OR "children s"[All Fields] OR "childrens"[All Fields] OR "childs"[All Fields] OR "child*"[All Fields] OR ("adolescences"[All Fields] OR "adolescency"[All Fields] OR "adolescent"[MeSH Terms] OR "adolescent"[All Fields] OR "adolescence"[All Fields] OR "adolescents"[All Fields] OR "adolescent s"[All Fields]) OR "adolescen*"[All Fields] OR ("infant"[MeSH Terms] OR "infant"[All Fields] OR "infants"[All Fields] OR "infant s"[All Fields]) OR "infant*"[All Fields] OR "young people"[All Fields] OR ("adolescent"[MeSH Terms] OR "adolescent"[All Fields] OR "youth"[All Fields] OR "youths"[All Fields] OR "youth s"[All Fields]) OR "juvenile*"[All Fields] OR "teenager*"[All Fields] OR "young adult*"[All Fields] OR ("child*"[All Fields] AND ("child, preschool"[MeSH Terms] OR ("child"[All Fields] AND "preschool"[All Fields]) OR "preschool child"[All Fields] OR ("pre"[All Fields] AND "school"[All Fields]) OR "pre school"[All Fields])) OR ("child*"[All Fields] AND ("health"[MeSH Terms] OR "health"[All Fields] OR "health s"[All Fields] OR "healthful"[All Fields] OR "healthfulness"[All Fields] OR "healths"[All Fields]))) AND ("United Kingdom"[All Fields] OR ("england"[MeSH Terms] OR "england"[All Fields] OR "england s"[All Fields] OR "englands"[All Fields]) OR ("wales"[MeSH Terms] OR "wales"[All Fields] OR "wales s"[All Fields]) OR ("scotland"[MeSH Terms] OR "scotland"[All Fields] OR "scotland s"[All Fields]) OR "Northern Ireland"[All Fields] OR "n ireland"[All Fields]) AND ("perception*"[All Fields] OR ("perceivable"[All Fields] OR "perceive"[All Fields] OR "perceiver"[All Fields] OR "perceiver s"[All Fields] OR "perceivers"[All Fields] OR "perceives"[All Fields] OR "perception"[MeSH Terms] OR "perception"[All Fields] OR "perceived"[All Fields] OR "perceiving"[All Fields]) OR "experience*"[All Fields] OR ("patient satisfaction"[MeSH Terms] OR ("patient"[All Fields] AND "satisfaction"[All Fields]) OR "patient satisfaction"[All Fields]) OR ("patient preference"[MeSH Terms] OR ("patient"[All Fields] AND "preference"[All Fields]) OR "patient preference"[All Fields]) OR ("attitude to health"[MeSH Terms] OR ("attitude"[All Fields] AND "health"[All Fields]) OR "attitude to health"[All Fields]) OR "facilitator*"[All Fields] OR "enabler*"[All Fields] OR "barrier*"[All Fields] OR ("patient acceptance of health care"[MeSH Terms] OR ("patient"[All Fields] AND "acceptance"[All Fields] AND "health"[All Fields] AND "care"[All Fields]) OR "patient acceptance of health care"[All Fields]))) AND ((2012/1/1:2022/2/21[pdat]) AND (english[Filter]))*

For peer review only

Additional file 3: Quality assessment

To achieve 'high' quality, at least five MMAT criteria had to be met, with breadth and depth of analysis, for 'medium' at least four criteria had to be met, and all other studies were rated 'low'. For a judgement of 'high' relevance, studies had to describe, with breadth and depth, factors influencing primary care access and privilege participants' perspectives.

NB there were no randomized controlled trials in the studies so the MMAT questions for section 2 have been removed here.

**SCREENING QUESTIONS**	S1. Are there clear research questions?<br>S2. Do the collected data allow to address the research questions?
**1. QUALITATIVE STUDIES**	1.1. Is the qualitative approach appropriate to answer the research question?<br>1.2. Are the qualitative data collection methods adequate to address the research question?<br>1.3. Are the findings adequately derived from the data?<br>1.4. Is the interpretation of results sufficiently substantiated by data?<br>1.5. Is there coherence between qualitative data sources, collection, analysis and interpretation?
**3. NON-RANDOMIZED STUDIES**	3.1. Are the participants representative of the target population?<br>3.2. Are measurements appropriate regarding both the outcome and intervention (or exposure)?<br>3.3. Are there complete outcome data?<br>3.4. Are the confounders accounted for in the design and analysis?<br>3.5. During the study period, is the intervention administered (or exposure occurred) as intended?
**4. QUANTITATIVE DESCRIPTIVE STUDIES**	4.1. Is the sampling strategy relevant to address the research question?<br>4.2. Is the sample representative of the target population?<br>4.3. Are the measurements appropriate?<br>4.4. Is the risk of nonresponse bias low?<br>4.5. Is the statistical analysis appropriate to answer the research question?
**5. MIXED METHODS STUDIES**	5.1. Is there an adequate rationale for using a mixed methods design to address the research question?<br>5.2. Are the different components of the study effectively integrated to answer the research question?<br>5.3. Are the outputs of the integration of qualitative and quantitative components adequately interpreted?<br>5.4. Are divergences and inconsistencies between quantitative and qualitative results adequately addressed?<br>5.5. Do the different components of the study adhere to the quality criteria of each tradition of the methods involved?

		SCREENING QUESTIONS		1. QUALITATIVE STUDIES					Total MMAT	W1 - quality	W2 - relevance
First author	Year	S1	S2	1.1	1.2	1.3	1.4	1.5			
Ahmaro et al	2021	Yes	Yes	Yes	Yes	Yes	Yes	Yes	5	High	High
Alexakis et al	2015	Yes	Yes	Yes	Yes	Yes	Yes	Yes	5	High	High
Appleton et al	2022	Yes	Yes	Yes	Yes	Yes	Yes	Yes	5	High	High
Bosley et al	2021	Yes	Yes	Yes	Yes	Yes	Yes	Yes	5	Medium	Medium
Brigham et al	2012	Can't tell	Can't tell	Yes	Yes	Yes	No	Can't tell	3	Low	Low
Coleman-Fountain et al	2020	Yes	Yes	Yes	Yes	Yes	Yes	Yes	5	High	Low
Condon et al	2020	Yes	Yes	Yes	Yes	Yes	Yes	Yes	5	High	Low
Corry and Leavey	2017	Yes	Yes	Yes	Yes	Yes	Yes	Yes	5	High	High
Crouch et al	2019	Yes	Yes	Yes	Yes	Yes	Yes	Yes	5	High	Medium
Dando et al	2019	Yes	Yes	Yes	Yes	Yes	Yes	Can't tell	4	Low	Low
Davey et al	2013	Yes	Yes	Yes	Yes	Yes	Can't tell	Yes	4	Medium	High
Dickson	2015	Yes	Yes	Yes	Yes	Yes	No	No	3	Low	Medium
Diwakar et al	2019	Yes	Yes	Yes	No	Yes	Yes	Yes	4	Medium	Medium
Eskytė et al	2021	Yes	Yes	Yes	Yes	Yes	Yes	Yes	5	High	Low
Fox et al	2017	Yes	Can't tell	Yes	Yes	Yes	Yes	Yes	5	High	High
French et al	2020	Yes	Yes	Yes	Yes	Yes	Yes	Yes	5	Medium	High
Henderson and Rubin	2014	Can't tell	Yes	No	No	Can't tell	Yes	Can't tell	3	Low	Low
Ingram et al	2013	Yes	Yes	Yes	Yes	Yes	Yes	No	4	Medium	High
Jobanputra and Singh	2020	Yes	Yes	Yes	No	Yes	No	Yes	3	Low	Medium
Jones et al	2017	Yes	Yes	Yes	Yes	Yes	Yes	Yes	5	High	High
Lewney et al	2019	Yes	Yes	Yes	Yes	Yes	Yes	Yes	5	High	Medium
McDonagh et al	2019	Yes	Yes	Yes	No	Yes	Yes	Yes	4	Medium	High
Mughal et al	2021	Yes	Yes	Yes	Yes	Yes	Yes	Can't tell	4	Medium	High
Muirhead et al	2017	Yes	Yes	Yes	Yes	Can't tell	Yes	Yes	4	Medium	Low
Neill et al	2016	Yes	Yes	Yes	Yes	Yes	Yes	Yes	5	High	High
Neill et al	2015	Yes	Yes	Yes	Yes	Yes	Yes	Yes	5	High	High
O'Brien et al	2017	Yes	Yes	Yes	Yes	Yes	Yes	Yes	5	High	High
Ochieng	2020	Yes	Yes	Yes	Yes	Yes	Yes	Yes	5	High	Low
Rapley et al	2021	Yes	Yes	Yes	Yes	Yes	Yes	Yes	5	High	High
Redsell et al	2013	Yes	Can't tell	Yes	Can't tell	No	Yes	No	2	Low	Low

For peer review only

Roberts et al	2014	Yes	Can't tell	Yes	No	Yes	Yes	No	3	Low	High
Roberts and Condon	2014	Yes	Yes	Yes	No	Yes	Yes	Yes	4	Low	Low
Satherley et al	2021	Yes	Yes	Yes	Yes	Yes	Yes	Yes	5	High	High
Turnbull et al	2021	Yes	Yes	Yes	Yes	Can't tell	Yes	Yes	4	Medium	High
Turner et al	2012	Yes	Yes	Can't tell	Yes	Yes	No	Yes	3	Low	High
Williams et al	2014	Yes	Yes	Yes	Can't tell	Yes	Yes	Yes	4	Low	Low
Williams et al	2012	No	Yes	Yes	Yes	Yes	Yes	Yes	5	Medium	Medium

		SCREENING QUESTIONS		3. NON-RANDOMIZED STUDIES					Total MMAT	W1 - quality	W2 - relevance
First author	Year	S1	S2	3.1	3.2	3.3	3.4	3.5			
O'Brien et al	2019	Yes	Yes	Yes	Yes	Yes	Yes	Yes	5	High	Low
Usher-Smith et al	2015	Yes	Can't tell	Yes	Yes	Yes	Can't tell	Yes	4	Medium	Medium
Yassaee et al	2017	Yes	Yes	Yes	Yes	Yes	Can't tell	Yes	4	High	Low

		SCREENING QUESTIONS		4. QUANTITATIVE DESCRIPTIVE STUDIES					Total MMAT	W1 - quality	W2 - relevance
First author	Year	S1	S2	4.1	4.2	4.3	4.4	4.5			
Coyle et al	2013	Yes	Yes	Can't tell	No	Yes	Yes	No	2	Low	Low

		SCREENING QUESTIONS		5. MIXED METHODS STUDIES					Total MMAT	W1 - quality	W2 - relevance
First author	Year	S1	S2	5.1	5.2	5.3	5.4	5.5			
Crocker et al	2013	Yes	Yes	Yes	Yes	Yes	Yes	No	4	Low	Low
Fox et al	2015	Yes	Yes	Yes	Yes	Yes	No	Yes	4	Medium	Medium
Rashed et al	2022	Yes	Yes	Can't tell	Can't tell	Yes	Yes	Can't tell	2	Low	Medium
Rickett et al	2021	Yes	Yes	Yes	Yes	Yes	Yes	Yes	5	Medium	High
Salaheddin and Mason	2016	Yes	Yes	Can't tell	Yes	Yes	Can't tell	Yes	3	Low	Low
Wilson et al	2021	Yes	Yes	Yes	Yes	Yes	No	Can't tell	3	Low	Low

Additional file 4: data synthesis

Data were synthesised using framework analysis (Gale et al, 2013) in Microsoft Excel. There were four key stages in the analysis process: 1) framework analysis 1 – a descriptive extraction and categorisation, 2) framework analysis 2 – a conceptual analysis, 3) refinement of the themes, and 4) mapping the barriers for sub-populations.

1. Framework analysis 1: a descriptive extraction and categorisation of the data

One reviewer (LH) carried out inductive coding and created an initial framework (a structured template) to summarise/reduce the data to focus on facilitators/barriers to primary healthcare. Data was extracted into an excel worksheet, with each study a row and column a code. The framework was revised iteratively as data from each study was added; by the tenth study, most of the codes were identified and remained the same. The codes were organised under six overarching descriptive categories: accessibility (1), health care beliefs/knowledge/preferences of caregivers (2) and CYP (3), relationship with HCP (4), quality of diagnosis/treatment (5), HCP knowledge/skills/networks/priorities (6). Data from the remaining studies was extracted, with new codes added or revised as best fit the data. Two reviewers (LH and EA) then examined the codes for each category independently and discussed emerging themes.

Table 1: Initial data analysis framework and codes

Overarching category	Codes
**Accessibility**	Multiple opportunities to engage with HCP
	Co-location
	Accessible premises and opening times
	Having information in native or accessible language
	Availability of informational or health resources
	Communication about entry to the service
	Difficulties meeting system structures and requirements
	Difficulties meeting threshold for other services
	Variation/inconsistency of entry criteria
	Patient residential impermanence
	Different ways of being able to access HCP
	Wait times to be seen by HCP
	Reduced services
	Free health care.
	Stigma
	Discrimination
**Parents' beliefs, knowledge, or preferences**	Parents' perceiving access important for child's health
	Familiarity with condition
	Stigma around mental health
	Valuing health professionals' expertise
	Lack of trust in medication/services in UK
	Parents', families or communities' perceiving they had sufficient knowledge themselves
	Parent knowledge or confidence about services or how to access them
	Being able to arrange an emergency appointment
	Parent difficulties attending due to other responsibilities e.g. childcare

	Cultural expectations of health professional
	Concerns about wasting GP time
	Parents feeling misunderstood and not listened to by health system
	Parental perception of gender norms
	Parents feeling shame/judgement
**YPs' beliefs, knowledge, or preferences**	YP knowledge about services or how to access them
	Knowledge/familiarity of condition
	HCP same/opposite sex/gender preferences
	YP anxiety about seeking help from GP or from dentist
	YP perceiving whether HCP would take them seriously, without judgement and be interested in them
	Having choice
	Self-testing
	YP Cultural Expectations of HCP
	YP Self Reliance
	YP Sensitivity relating to family context
**Relationship with HCP**	Clear communication from health professional
	Health professional being friendly, approachable and reassuring (or not)/Personable Qualities of HCP
	Continuity of care
	Trusted relationship
	Confidentiality
	Duration of time in the consultation to listen to concerns
	Parents attending with YP
**Quality of diagnosis or treatment**	Accuracy of test result
	Timeliness of test result
**HCP knowledge, skills, networks, and priorities**	Health professionals having appropriate education and training
	Being able to undertake a good holistic assessment of family needs
	Health professionals' having local knowledge
	Health professionals having signposting, referral and co-ordination skills.
	Collaboration (or lack of) between services
	Priority given to health topic by professional
	HCP Professional Perceptions of Health Topic
	HCP perceptions of the individual

2. Framework analysis 2: a conceptual analysis and development of themes

After reflection and discussion between the reviewers, the data was re-organised conceptually following the journey of a caregiver or CYP from first noticing a health issue and deciding to seek help to attending a consultation and potentially being referred to secondary or adult services, influenced by the work of Ford et al's (2016). Ford et al outlined the following steps in access to primary care for socioeconomically disadvantage older people in rural areas: problem identified, decision to seek help, actively seek help, obtain appointment, get to the appointment, primary care interaction, and outcome.

Five higher-order themes were constructed from the data (see table 2). The data in the initial framework was re-organised, putting data relating to the new a-priori themes into separate Excel

worksheets. Through inductive analysis of the data under each theme, new codes and sub-themes were constructed.

Table 2: Initial higher-order themes

Decision to access care
Reaching and entering services
Communication and trust between HCPs, caregivers and CYP
Gaps in HCP knowledge
General practice as a gatekeeper to, or a holding space for, secondary or adult care

To visualise whether any codes and themes were particularly pertinent for specific sub-populations with higher health needs, data was colour-coded: CYP from deprived areas, looked after children, non-White British CYP, CYP with SEN or disabilities, CYP with chronic conditions, and CYP with mental health problems. Where a study looked at two groups, text was coded in one colour and the cell background another. The sub-populations were selected from CYP target populations and focus clinical areas in the 'Core20Plus5', the national NHS England approach to support the reduction of health inequalities, though we included evidence for any chronic condition instead of the strategy's focus on asthma, diabetes, and epilepsy.

3. Refinement of the themes

The themes and sub-themes were mapped out visually in Powerpoint and discussed with the wider team. The decision to access care, reaching and entering services, and communication and trust formed a repeatable pattern of experiences that affected access to primary care. A consensus was reached among the team that the three sub-themes under "General practice as a gatekeeper…" fit within "Reaching and entering services" and "Communication and trust", and gaps in HCP knowledge impacted on communication and trust, and could be subsumed within that theme. These changes were made and final three over-arching themes were constructed.

4. Mapping the barriers for sub-populations of CYP with higher health needs

Sub-themes that were reported particularly for key sub-populations of interest (see table 3) were systematically mapped into a table.

Table 3: sub-populations of interest

**Author (year)**	CYP with mental health problems	CYP from deprived areas	CYP from non-White British communities	CYP with SEND	CYP with chronic health conditions	Looked after children
Ahmaro et al (2021)						
Alexakis et al (2015)			✓		✓	
Appleton et al (2022)	✓					
Bosley et al (2021)						
Brigham et al (2012)						
Coleman-Fountain et al (2020)	✓			✓		
Condon et al (2020)			✓			
Corry and Leavey (2017)	✓					
Coyle et al (2013)				✓		
Crocker et al (2013)						

For peer review only

Crouch et al (2019)	✓					
Dando et al (2019)			✓			
Davey et al (2013)						
Dickson (2015)		✓				
Diwakar et al (2019)					✓	
Eskytė et al (2021)		✓				
Fox et al (2017)		✓		✓		
Fox et al (2015)	✓					
French et al (2020)				✓		
Henderson and Rubin (2014)		✓				
Ingram et al (2013)						
Jobanputra and Singh (2020)	✓					
Jones et al (2017)						
Lewney et al (2019)						
McDonagh et al (2020)						
Mughal et al (2021)	✓					
Muirhead et al (2017)						✓
Neill et al (2016)*		✓	✓			
Neill et al (2015)*		✓	✓			
O'Brien et al (2019)	✓					
O'Brien et al (2017)	✓					
Ochieng (2020)			✓			
Rapley et al (2021)					✓	
Rashed et al (2022)						
Redsell et al (2013)						
Rickett et al (2021)						
Roberts et al (2014)	✓	✓				
Roberts and Condon (2014)						
Salaheddin and Mason (2016)	✓					
Satherley et al (2021)		✓				
Turnbull et al (2021)						
Turner et al (2012)						
Usher-Smith et al (2015)					✓	
Williams et al (2014)						✓
Williams et al (2012)			✓			
Wilson et al (2021)				✓		
Yassaee et al (2017)						

For peer review only

Author	Deciding to access care: *Identifying a health issue as worthy of attention from a HCP*	Deciding access care: *Consulting with family and social networks about symptoms*	Deciding to access care: *Surmounting stigma, experiences of discrimination, and embarrassment*
Ahmaro *et al.* (2021)			1
Alexakis *et al.* (2015)		1	
Appleton *et al.* (2022)			
Bosley *et al.* (2021)			
Brigham *et al.* (2012)			
Coleman-Fountain *et al.* (2020)	1		
Condon *et al.* (2020)			1
Corry and Leavey (2017)			1
Coyle *et al.* (2013)			
Crocker *et al.* (2013)			
Crouch *et al.* (2019)			
Dando *et al.* (2019)			1
Davey *et al.* (2013)			
Dickson (2015)			
Diwakar *et al.* (2019)			
Eskytė et al. (2021)	1		
Fox *et al.* (2017)		1	1
Fox *et al.* (2015)			
French *et al.* (2020)			1
Henderson and Rubin (2014)	1		
Ingram *et al.* (2013)	1	1	
Jobanputra and Singh (2020)			
Jones *et al.* (2017)			1
Lewney *et al.* (2019)			
McDonagh *et al.* (2020)			1
Mughal *et al.* (2021)	1		1
Muirhead *et al.* (2017)			
Neill *et al.* (2016)*	1	1	
Neill *et al.* (2015)*	1	1	
O'Brien *et al.* (2019)			
O'Brien *et al.* (2017)			1

For peer review only

Ochieng (2020)			1
Rapley *et al.* (2021)			
Rashed *et al.* (2022)			
Redsell *et al.* (2013)			
Rickett *et al.* (2021)			1
Roberts *et al.* (2014)			
Roberts and Condon (2014)	1		
Salaheddin and Mason (2016)			1
Satherley *et al.* (2021)			1
Turnbull *et al.* (2021)			1
Turner *et al.* (2012)			1
Usher-Smith *et al*. (2015)	1		
Williams et al. (2014)			
Williams *et al.* (2012)			1
Wilson et al. (2021)			
Yassaee *et al.* (2017)			

For peer review only

Reaching and entering services: *The supply of services*	Reaching and entering services: *Knowledge, confidence, and access to information about services and treatment*	Reaching and entering services: *Family-friendly healthcare settings*	Reaching and entering services: *Delayed or rejected referrals to secondary or adult care*
1			
		1	
1	1		
	1		
1			
	1		1
1	1		
1			
	1		
1	1		
	1		
		1	1
1	1		1
	1		
1			
			1
1	1		
1	1		
1	1		
1	1		
1			
1		1	
1	1		
			1
	1		1

For peer review only

1	1		1
1	1	1	
	1		
1		1	
1			
1			
1			
		1	
1			

For peer review only

Communication and trust: *Enabling CYP and caregivers to disclose their concerns*	Communication and trust: *Confidentiality and parental involvement*	Communication and trust: *HCP knowledge and competence*	Design
1			Qualitative; interviews
1		1	Qualitative; interviews
1		1	Qualitative; interviews
1			Qualitative; focus groups and interviews
			Qualitative; focus groups
			Qualitative; interviews
			Qualitative; focus groups
1	1	1	Qualitative; focus groups
		1	Quantitative; survey
			Mixed methods; survey and structured interviews
1			Qualitative; interviews
			Qualitative; interviews
1	1		Qualitative; interviews
			Qualitative; interviews
1		1	Qualitative; interviews
		1	Qualitative; interviews and focus groups
			Qualitative; interviews
1	1	1	Mixed methods; online survey and interviews
		1	Qualitative; interviews
			Qualitative; focus groups and interviews
		1	Qualitative; focus groups and interviews
	1	1	Qualitative; interviews
1			Qualitative; interviews
		1	Qualitative; interviews
1			Qualitative; interviews
1	1		Qualitative; interviews
			Qualitative; focus groups
1		1	Qualitative; focus groups and interviews
1			Qualitative; focus groups and interviews
			Quantitative; cross-sectional survey
		1	Qualitative; interviews

For peer review only

		1	Qualitative; focus groups
		1	Qualitative; interviews
1			Mixed; survey with closed and open questions
1		1	Qualitative; interviews
		1	Mixed; survey with closed and open questions
1	1	1	Qualitative; interviews
			Qualitative; interviews
			Mixed; survey with closed and open questions
1			Qualitative; interviews
1			Qualitative; interviews
1		1	Qualitative; interviews
			Quantitative; survey
1			Qualitative; interviews and routinely collected data
		1	Qualitative; focus groups
			Quantitative; telephone survey
1			Quantitative; cross-sectional survey

For peer review only

Who	Quality rating
YP	High
YP	High
YP and caregivers	High
Caregivers	Medium
HCPs	Low
YP	High
Caregivers	High
YP	High
HCPs	Low
Caregivers	Low
Caregivers	High
Caregivers	Low
YP	Medium
Caregivers	Low
Caregivers	Medium
HCPs	High
Caregivers	High
HCPs	Medium
HCPs, adults with ADHD,	Medium
HCPs, school staff, caregivers	Low
Caregivers	Medium
HCPs	Low
YP	High
HCPs	High
YP	Medium
YP	Medium
Caregivers	Medium
Caregivers	High
Caregivers	High
HCPs	High
HCPs	High

For peer review only

Caregivers and HCPs	High
Caregivers and HCPs	High
Caregivers and YP	Low
HCPs	Low
Caregivers	Medium
HCPs	Low
Caregivers	Low
YP	Low
Caregivers	High
YP	Medium
Caregivers	Low
Caregivers	Medium
HCPs, social workers, CYP,	Low
Caregivers	Medium
Optometric practices	Low
YP	High

For peer review only

Supplementary tables

Table 1: Characteristics of studies included

**Author (year)** ***sample location***	***Primary healthcare setting:*** **main focus of study**	**Design**	**Sample population**	**Sample size**	**Focused on CYP in the following age bands:**			**Quality rating**	**Relevance rating**
					**<5**	**5–15**	**16–25**	**High/med/ low**	**High/med/ low**
Ahmaro *et al.* (2021) *England*	*Pharmacies*: perceptions of YP about sexual health and chlamydia testing and chlamydia treatment.	Qualitative; interviews	YP	26			✓	High	High
Alexakis *et al.* (2015) *England*	*General practice:* understanding the specific issues and service needs of YP with inflammatory bowel disease from black and ethnic minority communities.	Qualitative; interviews	YP	20			✓	High	High
Appleton *et al.* (2022) *England*	*General practice:* exploring the experiences and views of CYP and caregivers of CYP receiving primary care support after child and adolescent mental health services.	Qualitative; interviews	YP and caregivers	14 YP and 13 parents			✓	High	High
Bosley *et al.* (2021) *England*	*General practice and health visiting*: mothers' views on the accessibility and expertise of healthcare professionals caring for their child's health.	Qualitative; focus groups and interviews	Caregivers	6 focus groups (16 parents) and 14 interviews	✓			Medium	Medium
Brigham *et al.* (2012) *England*	*Health visiting*: health visitors' (HVs) perceptions of their role and skills, how they share expertise, and work with other agencies.	Qualitative; focus groups	HCPs	4 focus groups (32 HVs)	✓			Low	Low
Coleman-Fountain *et al.* (2020) *n/k*	*General practice:* exploring how autistic young adults understand and manage mental health problems.	Qualitative; interviews	YP	19			✓	High	Low
Condon *et al.* (2020) *England*	*General practice and health visiting*: parents' experiences of using child health services for their children post-migration from Romania, Poland, Pakistan or Somalia.	Qualitative; focus groups	Caregivers	Five focus groups (28 parents)	✓			High	Low

For peer review only

Corry and Leavey (2017) *Northern Ireland*	*General practice*: adolescents' attitudes to consulting their GP about psychological problems.	Qualitative; focus groups	YP	Nine focus groups (54 YP)		✓	✓	High	High
Coyle *et al.* (2013) *Northern Ireland and Scotland*	*Dental care*: investigated practitioners' willingness to treat adolescents with learning disabilities (LD) in primary dental care.	Quantitative; survey	HCPs	300		✓	✓	Low	Low
Crocker *et al.* (2013) *Wales*	*General practice:* identifying differences between children who consulted a GP and those who did not before the day of hospital presentation with pneumonia or empyema.	Mixed methods; survey and structured interviews	Caregivers	151 survey participants of whom 79 were interviewed	✓	✓		Low	Low
Crouch *et al.* (2019) *England*	*General practice:* understanding families' experiences of seeking help and accessing specialist treatment for childhood anxiety	Qualitative; interviews	Caregivers	16		✓		High	Medium
Dando *et al.* (2019) *England*	*General practice:* understanding the healthcare experiences of Albanian survivors of modern slavery and sexual exploitation	Qualitative; interviews	Caregivers	7 participants of whom 6 were caregivers	✓	n/k		Low	Low
Davey *et al.* (2013) *England*	*General practice and walk-in centres*: explored the needs and experiences of young adults of primary healthcare services.	Qualitative; interviews	YP	20			✓	Medium	High
Dickson (2015) *Northern Ireland*	*Dental care*: parents' perceptions of factors influencing dental registrations of children living within a Sure Start area.	Qualitative; interviews	Caregivers	8	✓			Low	Medium
Diwakar *et al.* (2019) *England*	*General practice:* understanding parent experiences with paediatric allergy pathways.	Qualitative; interviews	Caregivers	18	✓	✓		Medium	Medium
Eskytė *et al.* (2021) *England*	*Health visiting*: organisational factors that obstruct HVs from speaking to parents of babies about oral health	Qualitative; interviews and focus groups	HCPs	3 focus groups (15 HVs) 3 interviews	✓			High	Low

For peer review only

Fox *et al.* (2017) *England*	*General practice and health visiting*: assessed what families affected by autism need and how health, education, and social care services can support them.	Qualitative; interviews	Caregivers	15	✓	✓		High	High
Fox *et al.* (2015) *England*	*General practice*: GP's capabilities, motivations and opportunities for discussing self-harm and to identify barriers to and enablers for discussing self-harm with YP.	Mixed methods; online survey and interviews	HCPs	28 (online survey) 10 (interviews)		✓	✓	Medium	Medium
French *et al.* (2020) *UK*	*General practice:* exploring the primary care experiences of referral and management of ADHD	Qualitative; interviews	HCPs, adults with ADHD, caregivers	5 primary HCP, 5 adults with ADHD, 5 caregivers, 5 secondary HCP	n/k	n/k	n/k	Medium	High
Henderson and Rubin (2014) *England*	*Dental care*: dental, school and family perspectives of an oral health promotion initiative to improve access for pre-school children in deprived communities.	Qualitative; focus groups and interviews	HCPs, school staff, caregivers and CYP	6 focus groups (24 dental practitioners), 9 interviews (school staff), 4 interviews (caregivers and their child)	✓	✓		Low	Low
Ingram *et al.* (2013) *n/k*	*General practice:* to explore parents' views on support and information needs prior to consulting when children have respiratory tract infections with a cough	Qualitative; focus groups and interviews	Caregivers	60	✓	✓		Medium	High
Jobanputra and Singh (2020) *England*	*General practice:* exploring GPs' views on the management of adolescents with mental health disorders	Qualitative; interviews	HCPs	8		✓	✓	Low	Medium
Jones *et al.* (2017) *England*	*General practice*: young adults' opinions of receiving chlamydia testing with condom provision, contraceptive information, and HIV testing.	Qualitative; interviews	YP	30			✓	High	High

For peer review only

Lewney *et al.* (2019) *England*	*Health visiting:* exploring how HVs feel about providing oral health advice and dealing with dental issues	Qualitative; interviews	HCPs	17	✓			High	Medium
McDonagh *et al.* (2020) *UK*	*General practice*: YPs' perspectives on barriers to chlamydia testing and potential intervention functions and implementation strategies to overcome identified barriers.	Qualitative; interviews	YP	28			✓	Medium	High
Mughal *et al.* (2021) *England*	*General practice*: the help-seeking behaviours, experiences of GP care, and access to the general practice of YP who self-harm.	Qualitative; interviews	YP	13			✓	Medium	High
Muirhead *et al.* (2017) *England*	*Dental care:* to understand foster carers' oral health knowledge, attitudes, and experiences of managing foster children's oral health.	Qualitative; focus groups	Caregivers	12		✓	✓	Medium	Low
Neill *et al.* (2016)***** *England*	*Primary care (all except dental and optometry)*: how parents from different socio-economic groups use the information to make decisions during acute childhood illness at home.	Qualitative; focus groups and interviews	Caregivers	Five focus groups (24 parents) and 3 interviews	✓			High	High
Neill *et al.* (2015)***** *England*	*Primary care (all except dental and optometry)*: parents' use of information resources during decision-making in acute childhood illness at home.	Qualitative; focus groups and interviews	Caregivers	Five focus groups (24 parents) and 3 interviews	✓			High	High
O'Brien *et al.* (2019) *England*	*General practice*: GPs experiences of barriers to and facilitators of identifying, managing, and accessing specialist services for anxiety disorders.	Quantitative; cross-sectional survey	HCPs	971	✓	✓		High	Low
O'Brien *et al.* (2017) *England*	*General practice:* explore the experiences of GPs in identification, management, and access to specialist services for anxiety disorders.	Qualitative; interviews	HCPs	20	✓	✓		High	High
Ochieng (2020) *England*	*Health visiting*: the sociocultural, family, and environmental factors that either influence healthy weight in black African children.	Qualitative; focus groups	Caregivers and HCPs	4 focus groups (30 parents) and 3 focus groups (32 HVs)	✓	✓		High	Low

For peer review only

Rapley *et al.* (2021) *England*	*Primary care (all except optometry):* exploring the experiences of care, from initial symptoms to initial referral to paediatric rheumatology.	Qualitative; interviews	Caregivers and HCPs	51 interviews with caregivers (related to 36 CYP), 11 interviews with HCPs	✓	✓	✓	High	High
Rashed *et al.* (2022) *England*	*Pharmacy and general practice:* exploring the experiences, barriers and recommendations of caregivers and YP regarding the use of community pharmacies for children.	Mixed; survey with closed and open questions	Caregivers and YP	213 caregivers and 20 YP	✓	✓	✓	Low	Medium
Redsell *et al.* (2013) *England*	*Health visiting*: investigated the beliefs and current practices of UK HVs concerning recognising and intervening with infants at risk of developing obesity.	Qualitative; interviews	HCPs	30	✓			Low	Low
Rickett *et al.* (2021) *Scotland, Wales, and England*	*General practice:* to understand the healthcare expectations and experiences of caregivers seeking support for their gender diverse children	Mixed; survey with closed and open questions	Caregivers	75	✓	✓		Medium	High
Roberts *et al.* (2014) *England*	*General practice*: GPs' experiences and perceptions of consulting with adolescents who present with psychological difficulties.	Qualitative; interviews	HCPs	19		✓	✓	Low	High
Roberts and Condon (2014) *England*	*Dental care:* exploring parental attitudes to pre-school oral health.	Qualitative; interviews	Caregivers	12	✓			Low	Low
Salaheddin and Mason (2016) *UK*	*General practice:* exploring the barriers to accessing mental health support among young adults.	Mixed; survey with closed and open questions	YP	203			✓	Low	Low
Satherley *et al.* (2021) *England*	*General practice*: how mothers living in deprived neighbourhoods support their children with health conditions.	Qualitative; interviews	Caregivers	8	✓	✓		High	High
Turnbull *et al.* (2021) *England*	*Pharmacy and sexual health clinic:* young women's experiences of accessing emergency contraception pills from pharmacies and sexual health clinics.	Qualitative; interviews	YP	21			✓	Medium	High

For peer review only

Turner *et al.* (2012) *England*	*General practice*: exploring parents' views and experiences of primary care as a treatment setting for childhood obesity.	Qualitative; interviews	Caregivers	15		✓		Low	High
Usher-Smith *et al.* (2015) *England*	*General practice and secondary care*: Explored the pathway to diagnosis of type 1 diabetes.	Quantitative; survey	Caregivers	87	✓	✓		Medium	Medium
Williams *et al.* (2014) *England and Wales*	*Dental care*: the impact of a community-based dental care pathway on children's dental care entering residential or foster care.	Qualitative; interviews and routinely collected data	HCPs, social workers, CYP, and caregivers	Routinely collected data on 89 CYP<br>Dental health professionals (n=6)<br>Social workers (n=2)<br>CYP (n=3)<br>Caregivers (n=5)	✓	✓	✓	Low	Low
Williams *et al.* (2012) *England*	*Not specified (preventative primary care services)*: Described African and African-Caribbean fathers' beliefs about fatherhood, health and preventive primary care services.	Qualitative; focus groups	Caregivers	9 focus groups (46 parents)	n/k	n/k	n/k	Medium	Medium
Wilson *et al.* (2021) *England*	*Optometric practices*: accessibility of eye care for children with typical development and those with autism.	Quantitative; telephone survey	Optometric practices	400	✓	✓		Low	Low
Yassaee *et al.* (2017) *England*	*General practice*: adolescents' experiences of their GP, whether poor reported GP experience was associated with worse physical and mental health measures and whether poor previous GP experience was linked to lower service utilisation.	Quantitative; cross-sectional survey	YP	5,335		✓		High	Low

For peer review only

Table 2: Health topic and CYP population studied

Health topic (e.g., dental, sexual)	Specific population	Primary healthcare setting	Citations
**Non-specific health condition (n=13)**	• Non-specific (n=5)<br>• African and African-Caribbean fathers (n=1)<br>• CYP from different socio-economic and ethnic groups (n=1)<br>• CYP living in deprived areas (n=1)<br>• CYP of Albanian survivors of modern slavery and sexual exploitation (n=1)<br>• CYP migrants from Romania, Poland, Pakistan, or Somalia (n=1)<br>• CYP with ADHD (n=1)<br>• CYP with autism from an ethnic minority/migrant community (n=1)<br>• South Asian and Gypsy/Travelling communities (n=1)	• General practice (n=4)<br>• General practice and health visiting (n=3)<br>• Primary care (all except dentist, n=2)<br>• General practice and walk-in centres (n=1)<br>• Health visiting (n=1)<br>• General practice and pharmacy (n=1)<br>• Not specified (preventative primary care services, n=1)	Condon et al (2020); Brigham et al (2012); Bosley et al. (2021); Dando et al. (2019); Davey et al. (2013); French et al. (2020); Fox et al. (2017); Neill et al. (2015); Neill et al. (2016); Rashed et al. (2022); Satherley et al. (2021); Williams et al. (2012); Yassaee et al. (2017)
**Mental health (n=11)**	• Non-specific (n=9)<br>• CYP living in deprived areas (n=1)<br>• CYP with autism (n=1)	• General practice (n=11)	Appleton et al. (2022); Coleman-Fountain et al. (2020); Corry and Leavey (2017); Crouch et al. (2019); Fox et al. (2015); Jobanputra and Singh (2020); Mughal et al. (2021); O'Brien et al. (2017); O'Brien et al. (2019); Roberts et al. (2014); Salaheddin and Mason (2016)
**Gender diversity (n=1)**	• Non-specific (n=1)	• General practice (n=1)	Rickett et al. (2021)
**Chronic conditions (n=4) (allergies, n=1; inflammatory bowel disease, n=1; juvenile idiopathic arthritis, n=1; type 1 diabetes, n=1)**	• Non-specific (n=3)<br>• Black and minority ethnic CYP (n=1)	• General practice (n=3)<br>• Primary care (all except optometry) (n=1)	Alexakis et al. (2015); Diwakar et al. (2019); Rapley et al. (2021); Usher-Smith et al. (2015)

For peer review only

**Physical health (n=4) (obesity, n=3; pneumonia or empyema, n=1; respiratory tract infections, n=1)**	• Non-specific (n=3)<br>• Black African CYP (n=1)	• General practice (n=2)<br>• Health visiting (n=2)	Crocker et al. (2013); Ingram et al. (2013); Ochieng (2020); Redsell et al. (2013); Turner et al. (2012)
**Oral health (n=8)**	• Non-specific (n=2)<br>• CYP in care (n=2)<br>• CYP living in deprived areas (n=3)<br>• CYP with learning disabilities (n=1)	• Dental care (n=6)<br>• Health visiting (n=2)	Coyle et al. (2013); Dickson (2015); Eskyte et al. (2021); Henderson and Rubin (2014); Lewney et al. (2019); Muirhead et al. (2017); Roberts and Condon (2014); Williams et al. (2014)
**Sexual health (n=4)**	• Non-specific (n=4)	• General practice (n=2)<br>• Pharmacy (n=1)<br>• Pharmacy and sexual health clinic (n=1)	Ahmaro et al. (2021); Jones et al. (2017); McDonagh et al. (2020); Turnbull et al. (2021)
**Optometry (n=1)**	• Younger CYP and CYP with autism (n=1)	• Optometric practices (n=1)	Wilson et al. (2021)

For peer review only
